# Direct Catalytic Asymmetric Synthesis of Pyrazolidine Derivatives

**DOI:** 10.1002/open.201200015

**Published:** 2012-06-21

**Authors:** Luca Deiana, Gui-Ling Zhao, Hans Leijonmarck, Junliang Sun, Christian W Lehmann, Armando Córdova

**Affiliations:** a Department of Natural Sciences, Engineering and Mathematics, Mid Sweden University85170 Sundsvall (Sweden) E-mail: acordova@organ.su.searmando.cordova@miun.se; b Department of Organic Chemistry, Arrhenius Laboratory, Stockholm University10691 Stockholm (Sweden); c Department of Structural Chemistry, Arrhenius Laboratory, Stockholm University10691 Stockholm (Sweden); d Berzelii Center EXSELENT on Porous Materials, Arrhenius Laboratory, Stockholm University10691 Stockholm (Sweden); e Max-Planck-Institut für KohlenforschungKaiser-Wilhelm-Platz 1, 45470 Mülheim an der Ruhr (Germany)

**Keywords:** 1,4-selectivity, asymmetric catalysis, cascade reactions, hemiaminals, metal-free catalysis, pyrazolidines

The importance and increased demand of pharmaceutically active azaheterocycles has urged the development of inexpensive and environmentally benign catalytic asymmetric technologies.[1, 2] In this context, the pyrazolidine and pyrazoline structural motif is present in several compounds with significant bioactivities, such as anti-inflammatory, antidepressant, anticancer, antibacterial and antiviral activities.[3, 4] These types of compounds are also important starting materials for the syntheses of azaprolines and diamines.[5]

In their seminal 1887 work, Fisher and Knövenagel reported that the reaction between acrolein and phenylhydrazine gave the corresponding pyrazoline under acidic conditions (Scheme 1).[6, 7] However, it was not until 2000 that the first enantioselective synthesis of pyrazolines from acrylamides by means of metal-catalyzed enantioselective [1,3]-dipolar cycloaddition was disclosed.[8] The subsequent asymmetric syntheses were also predominantly based on metal-catalyzed [1,3]-dipolar cycloadditions using dipoles and dipole precursors such as diazoalkanes and nitrile imines, respectively, as starting materials.[9] The synthesis of 3-pyridyl-4-aryl pyrazolines was also accomplished by a metal-mediated aza-Michael cyclocondensation cascade transformation with moderate enantioselectivity.[10] Simultaneously, Sibi and coworkers reported an elegant pyrazilidinone synthesis using a metal-catalyzed enantioselective aza-Michael/cyclization cascade transformation.[11] In the realms of metal-free catalysis, List and Müller recently reported the first catalytic asymmetric Fischer synthesis of pyrazolines through a chiral phosphoric acid-catalyzed 6π-electrocyclization of α,β-unsaturated hydrazones.[12] Shortly after, Briere and coworkers reported an elegant enantioselective synthesis of pyrazolines using β-aryl enones as starting materials by means of phase-transfer catalysis.[13] This was recently expanded by Deng and coworkers to aliphatic-substituted enones.13b Chiral substituted pyrazolidines can also be synthesized by metal and metal-free catalysis.[14] Based on the importance of diazaheterocycles and our research interest in asymmetric synthesis,[15] we decided to embark on the development of a direct enantioselective route to pyrazolidines by metal-free catalysis. The retrocatalytic analysis suggested that a possible asymmetric synthesis of these compounds would be through a chiral amine-catalyzed[16] Michael/hemiaminal cascade reaction between a suitable hydrazine compound and an α,β-unsaturated aldehyde that would favor 1,4-addition over 1,2-addition (Scheme 2). Moreover, we envisioned that the subsequent hemiaminal formation would push the equilibrium of the reversible azaconjugate addition step towards product formation.[17] During our studies one elegant report appeared on the direct catalytic synthesis of pyrazolidines derivatives based on this strategy.[18] Interestingly, this reaction did not work for β-arylsubstituted enals.

**Scheme 1:**
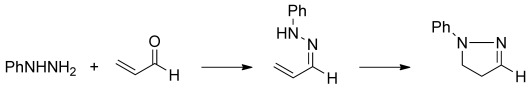
Acrolein and phenylhydrazine give the corresponding pyrazoline under acidic conditions.[6, 7]

**Scheme 2:**
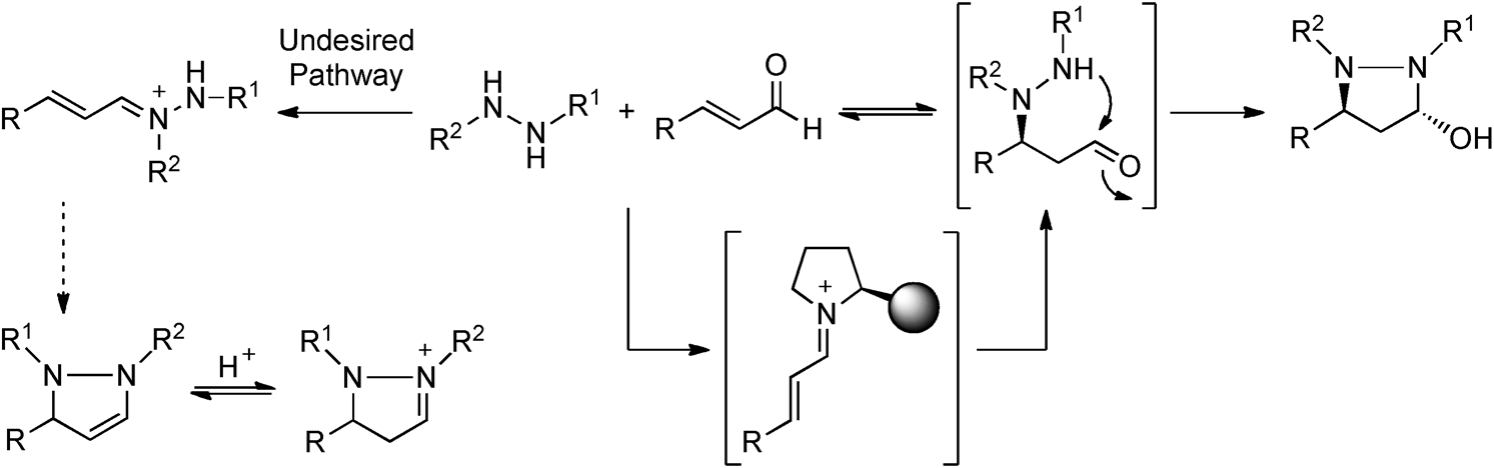
Michael/hemiaminal cascade reaction between a suitable hydrazine derivative and an α,β-unsaturated aldehyde that would favor 1,4-addition over 1,2-addition.

Herein, we present a highly enantioselective entry to pyrazolidine derivatives with 98–99 % *ee*, which proceeds via a metal-free, catalytic 1,4-specific cascade transformation between di-1,2-*N*-protected hydrazine and α,β-unsaturated aldehydes.

We began our studies by investigating the reaction between cinnamic aldehyde **1 a** and di-1,2-*N*-*tert*-butoxycarbonyl (Boc)-protected hydrazine **2 a** using different catalysts and conditions (Table 1). To our delight, the cascade reaction gave the corresponding 3-hydroxypyrazolidine **3 a** as the only product with high enantioselectivity when bulky, chiral pyrrolidine derivative **4** was used as the catalyst. Notably, the employment of chiral amines **4 a**–**c**[19] as catalysts delivered **3 a** with high to excellent enantioselectivities in toluene, trifluoromethyl benzene (PhCF_3_) and tetrahydrofuran (THF), respectively (Entries 2, 4, 7–17).[20] For example, protected prolinol **4 a** catalyzed the assembly of **3 a** in an asymmetric fashion in 54 % yield with 98 % *ee* at room temperature (Entry 7). In all cases, product **4 a** was formed exclusively as its α-anomer as determined by ^1^H NMR analysis of the crude reaction mixture. Moreover, our results indicate that the conversion did not significantly increase after prolonged reaction times. The addition of acid or base did not significantly effect the reaction (Entries 8 and 9). However, decreasing the temperature to 4 °C increased the yield and *ee* of **3 a** (64 % yield, >99 % *ee*, Entry 15). Thus, nearly enantiomerically pure **3 a** can be synthesized under these reaction conditions, however, the reaction rate decreases.

**Table 1:** Conditions used for screening[a]

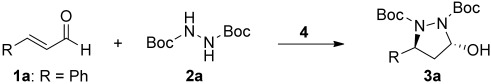							
Entry	Catalyst		Solvent	Time [h]	T [°C]	Yield [%][b]	*ee* [%][c]
1	**4 a**	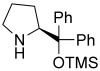	CHCl_3_	113	RT	33	76
2	**4 a**		THF	113	RT	23	99
3	**4 a**		CH_3_CN	113	RT	24	63
4	**4 a**		PhCF_3_	48	RT	46	95
5	**4 a**		DMF	94	RT	18	83
6	**4 a**		MeOH	93	RT	33	18
7	**4 a**		toluene	42	RT	54	98
8	**4 a**		toluene	42	RT	54[d]	98[d]
9	**4 a**		toluene	42	RT	42[e]	98[e]
10	**4 a**		toluene	20	RT	43[f] (53)[g]	99[f]
11	**4 a**		toluene	52	RT	48[f] (59)[g]	98[f]
12	**4 a**		toluene	53	40	27	86
13	**4 a**		toluene	119	4	56[f]	98[f]
14	**4 a**		toluene	72	4	57 (60)[g]	>99
15	**4 a**		toluene	144	4	64 (68)[g]	>99
16	**4 b**	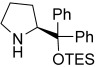	toluene	66	RT	44	98
17	**4 c**	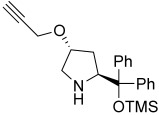	toluene	67	RT	26	99
18	**4 d**	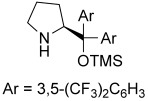	toluene	66	RT	traces	n.d.

[a] *Reagents and conditions:* hydrazine **2 a** (0.3 mmol), aldehyde **1 a** (0.25 mmol), catalyst **4** (20 mol %), solvent (0.5 mL). The reaction was stirred for the given time and temperature.

[b] Isolated yield after silica-gel column chromatography.

[c] Determined by chiral HPLC analysis. The α:β ratio of **3 a** was always >20:1 as determined by ^1^H NMR analysis of the crude reaction mixture.

[d] 20 mol % AcOH was added.

[e] NaOAc (1.1 equiv) was added.

[f] toluene (0.3 mL).

[g] Conversion as determined by ^1^H NMR analysis of the crude reaction mixture.

With these results in hand, we decided to probe the metal-free catalytic 1,3-diaminations of enals **1** (0.25 mmol) with **2 a** (0.3 mmol) as the amine source, **4 a** (20 mol %) as the amine catalyst and toluene (0.5 mL) as the solvent at 4 °C. The catalytic cascade reactions were highly chemoselective and gave the corresponding 3-hydroxypyrazolidines **3 a**–**k** as the only products in moderate to high yields with excellent *ee* values (98–99 %; Table 2). Thus, the aza-addition step was 1,4-specific. Moreover, all 3-hydroxypyrazolidines were formed exclusively as their α-anomers as determined by ^1^H NMR analysis of the crude reaction mixture. In comparison, the α-anomer is also the most stable conformer in the formation of other hydroxy-substituted, heterocyclic five-membered hemiaminals and hemiacetals, such as 5-hydroxypyrrolidines and 5-hydroxyoxazolidines, respectively.17a–e We next decided to investigate the effect of the N-protective group. This was accomplished by selecting di-1-*N*-Boc-2-*N*-benzyloxycarbonyl (Cbz)-protected hydrazine **2 b** as the dinitrogen source for the reaction with cinnamic aldehyde **1 a** (Scheme 3). The **4 a**-catalyzed cascade reaction gave corresponding 3-hydroxypyrazolidines **5 a** and **5′ a** in a 58:42 ratio and 66 % combined yield with 94 % and 98 % *ee*, respectively. Moreover, if a highly regioselective reaction is desired, a di-1,2-*N*-Boc-*N*-*para*-toluenesulfonyl (Tosyl)-protected hydrazine derivative should be employed as the nucleophile, since only the Boc-protected nitrogen will attack the β-aryl-substituted enal, as demonstrated above.

**Table 2:** Metal-free catalytic 1,3-diamination of enals 1[a]

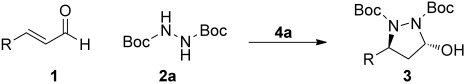				
Entry	Pyrazolidine product **3**		Yield [%][b]	*ee* [%][c]
1	**3 a**	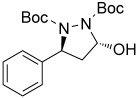	64	>99
2	**3 b**	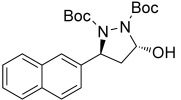	48	98
3	**3 c**	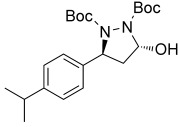	47	>99
4	**3 d**	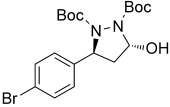	62	99
5	**3 e**	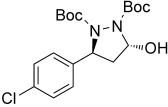	59	>99
6	**3 f**	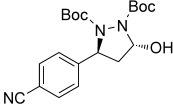	59	99
7	**3 g**	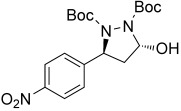	77	>99
8	**3 h**	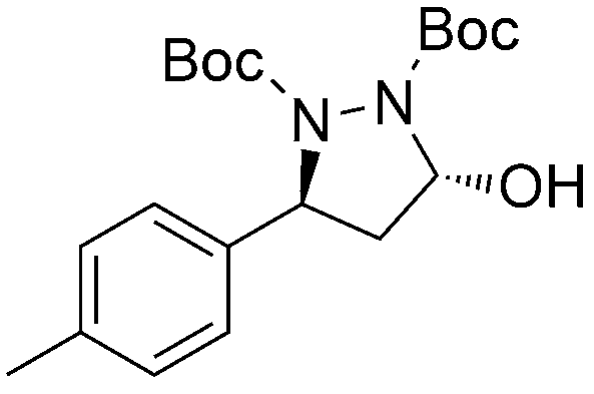	45	>99
9	**3 i**	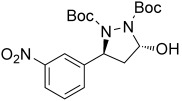	68	99[d]
10	**3 j**	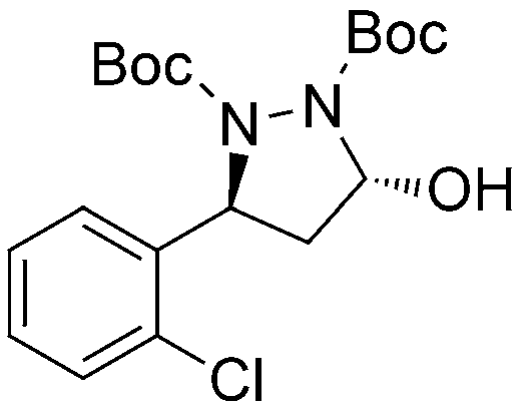	58	99[d]
11	**3 k**	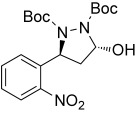	66	99[d]

[a] *Reagents and conditions:* **2 a** (0.3 mmol), **1** (0.25 mmol), **4 a** (20 mol %), toluene (0.5 mL), 4 °C, 144 h.

[b] Isolated yield after silica-gel column chromatography.

[c] Determined by chiral HPLC analysis. The α:β ratio of **3** was always >20:1 as determined by ^1^H NMR analysis of the crude reaction mixture.

[d] Reaction time was 92 h.

**Scheme 3:**
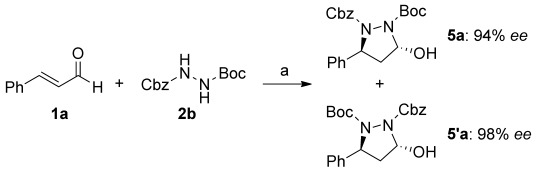
*Reagents and conditions:* a) 4 a (20 mol %), toluene, 4 °C, 88 h, 66 %, 58:42 ratio 5 a:5′ a.

The 3-hydroxypyrazolidines **3** were also versatile synthons for the asymmetric synthesis of other pyrazolidine derivatives. This was exemplified by the syntheses of pyrazolidines **6 a** and **7 a** (Scheme 4). Thus, highly diastereoselective Lewis acid-mediated allylation of **3 a** with allyltrimethylsilane gave pyrazolidine **6 a** with >19:1 *d.r*.

**Scheme 4:**
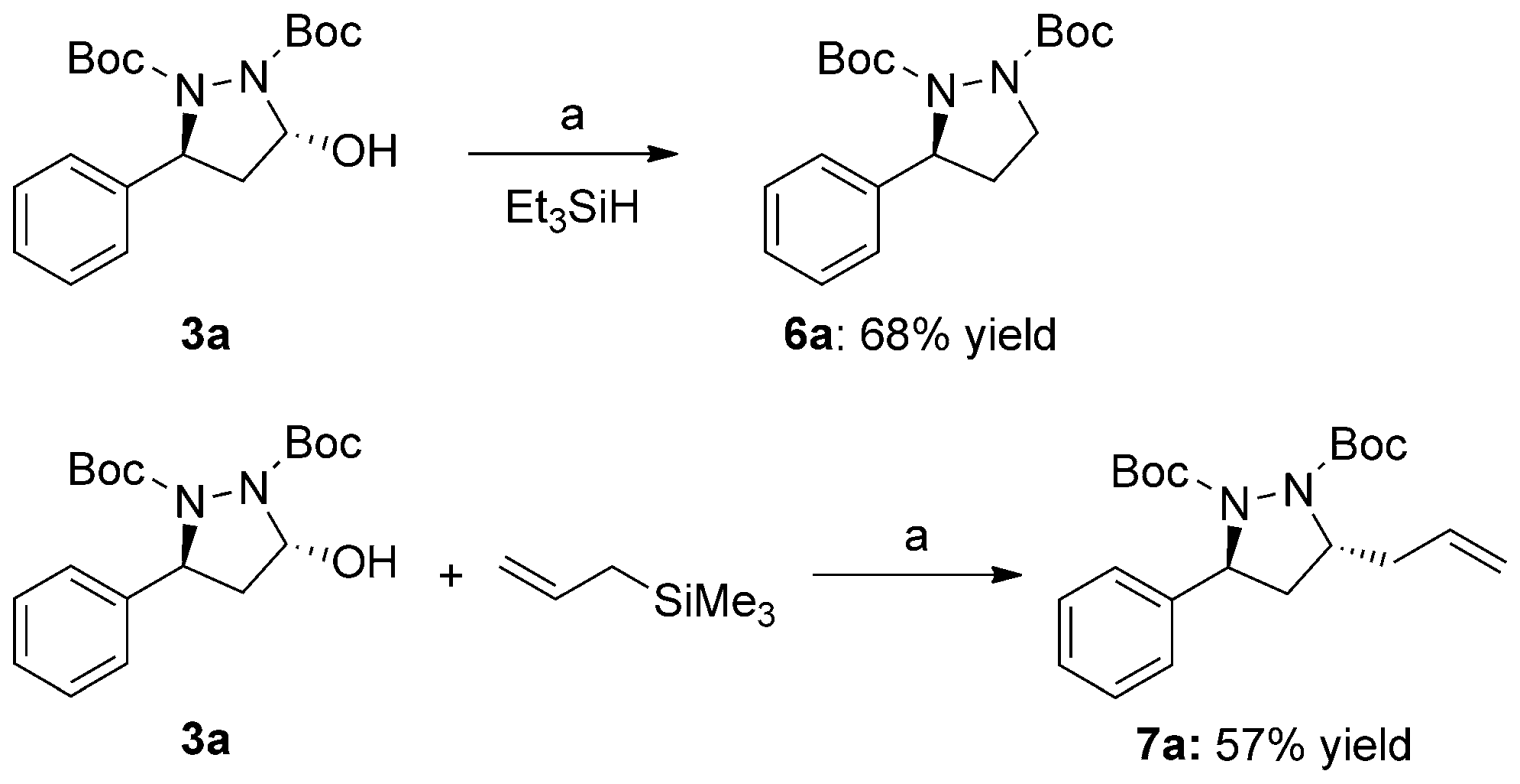
*Reagents and conditions:* a) BF_3_•Et_2_O, N_2_, CH_2_Cl_2_, −48 °C.

We also investigated the Fischer-type reaction between enal **1 a** and *N*-Boc-hydrazine **2 c** in the presence of chiral amine **4 a** (Scheme 5). After 18 h, the reaction was quenched and hydrazone **8 a** and dimer **9 a** (>19:1 *d.r*.) were isolated in 73 and 13 % yield, respectively. Thus, the initial 1,2-addition of the unprotected nitrogen of **2 c** to the enal **1 a** was the predominant pathway (Scheme 2). The experiment also shows the importance of having a di-1,2-*N*-protected hydrazine derivative in order to achieve excellent 1,4-selectivity. The highly diastereoselective formation of dimer **9 a** might occur via an initial chiral amine-catalyzed stereoselective aza-Michael/cyclization sequence (Scheme 1) that would give intermediates **10 a** and **11 a**, respectively, which then could dimerize to form **9 a**. Although dimer **9 a** was optical active, we were not able to determine the *ee* by chiral HPLC analysis. Prolonged reaction times or heating did not significantly improve the yield of **9 a**.

**Scheme 5:**
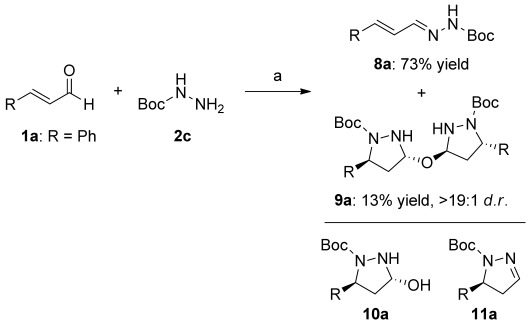
*Reagents and conditions:* a) 4 a (20 mol %), toluene, RT, 18 h.

The absolute and relative configuration of 3-hydroxypyrazoline derivatives **3** were determined by X-ray analysis of **3 i (**CCDC 855991),[21] which established that the (3*R*,5*S*) enantiomer had been formed (Figure 1). Thus, performing the enantioselective cascade transformation with (*S*)-**4 a** as the catalyst delivers the corresponding 3-hydroxypyrazoline derivatives (3*R*,5*S*)-**3**.

**Figure 1:**
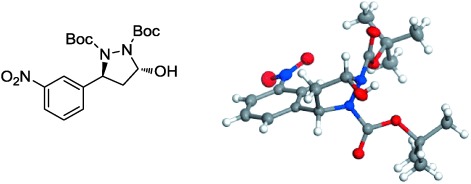
Chemical structure and ORTEP image of crystalline compound 3 i.

Based on the absolute configuration of pyrazolidine derivatives **3**, we propose the reaction mechanism shown in Scheme 6 to account for the observed stereochemistry. In accordance, iminium formation between chiral amine **4** and enal **1** delivers iminium intermediate **I**.[22] Next, a nucleophilic aza-Michael attack on the *si*-face of iminium intermediate **I** by hydrazine **2** delivers enamine intermediate **II**. Subsequent protonation and hydrolysis of iminium intermediate **III** regenerates chiral amine catalyst **6** and provides Michael-aldehyde intermediate **7**, which undergoes an intermolecular 5-*exo*-trig cyclization by its NHBoc group at the *re*-face of the aldehyde moiety to form the corresponding 3-hydroxypyrazolidine derivative **3**. The final hemiaminal formation pushes the equilibrium of the aza-Michael reaction towards product formation.

**Scheme 6:**
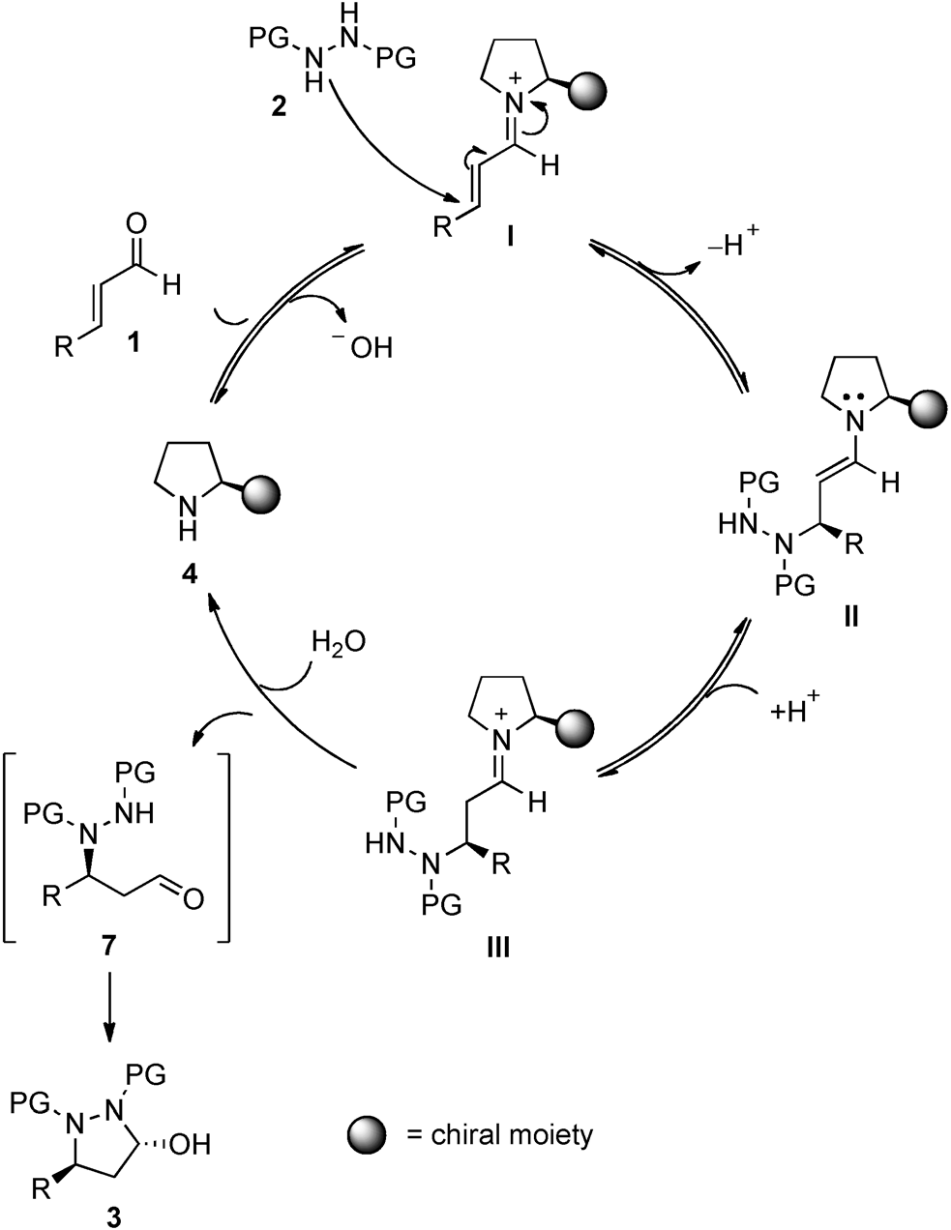
Proposed reaction mechanism.

In summary, we have developed a highly chemo- and enantioselective 1,3-diamination of α,β-unsaturated aldehydes with diprotected hydrazine derivatives as the dinitrogen source. The transformation was catalyzed by readily available chiral amines and proceeds via a direct catalytic metal-free aza-Michael/hemiaminal cascade sequence and delivers functional 3-hydroxypyrazolidine derivatives with 98–99 % *ee* in one step. Moreover, the transformation is a direct entry to other pyrazolidine derivatives in two steps. In this context, a subsequent Lewis acid-mediated allylation reaction gave access to 5-allyl-substituted pyrazolidines with excellent diastereoselectivity. It is noteworthy that the use of a monoprotected hydrazine as the dinitrogen source led predominantly to hydrazone formation (1,2-selective). Thus, the use of a di-1,2-*N*-protected hydrazine derivate was essential to switch the chemoselectivity and make the reaction 1,4-selective.

## Experimental Section

**Typical experimental procedure for the catalytic asymmetric synthesis of 3-hydroxypyrazolidine derivatives 3**: Nucleophile **2** (0.30 mmol) was added to a stirred solution of aldehyde **1** (0.25 mmol, 1.0 equiv) and catalyst **4 a** (0.05 mmol, 20 mol %) in toluene (0.5 mL) at 4 °C. The reaction was vigorously stirred at this temperature for the reported time. The crude reaction mixture was directly loaded on and purified by silica-gel chromatography (pentane/EtOAc or toluene/EtOAc) to afford the corresponding pyrazolidine derivative **3**.

CCDC 855991 http://www.ccdc.cam.ac.uk/cgi-bin/catreq.cgi(**3 i**) contains the supplementary crystallographic data for this paper. These data can be obtained free of charge from The Cambridge Crystallographic Data Centre via http://www.ccdc.cam.ac.uk/data_request/cif.

Full experimental procedures, NMR, HPLC and HRMS spectra for all newly described compounds can be found in the Supporting Information.

## Supplementary material

Detailed facts of importance to specialist readers are published as ”Supporting Information”. Such documents are peer-reviewed, but not copy-edited or typeset. They are made available as submitted by the authors.
