# Supplementary material for: Direct Catalytic Asymmetric Synthesis of Pyrazolidine Derivatives
Source: ChemistryOpen. 2012 Jun 21;1(3):134–9. doi: 10.1002/open.201200015 (PMC3922451; doi:10.1002/open.201200015)
Supplement: Supplementary file 1 [file open0001-0134-SD1.pdf]

Supporting Information

© Copyright Wiley-VCH Verlag GmbH & Co. KGaA, 69451 Weinheim, 2012

Direct Catalytic Asymmetric Synthesis of Pyrazolidine Derivatives

Luca Deiana,[b, d] Gui-Ling Zhao,[b, d] Hans Leijonmarck,[b, d] Junliang Sun,[c, d] Christian W. Lehmann,[e] and Armando Córdova*[a, b, d]

open_201200015_sm_miscellaneous_information.pdf

General methods: Chemicals and solvents were either purchased *puriss p. A.* from commercial suppliers or purified by standards techniques. For thin-layer chromatography (TLC), silica gel plates Merck 60 F254 were used and compounds were visualized by irradiation with UV light and/or by treatment with a solution of ammoniummolybdate (100 g), $Ce(SO_4)_2$ (2 g) and 10% H_2SO_4 (1 L) followed by heating or by treatment with a solution of potasium permanganate (3 g), K_2CO_3 (20 g), 5% aq. NaOH (5 mL) and water (300 mL) followed by heating. Flash chromatography was performed using silica gel Merck 60 (particle size 0.040-0.063 mm), ^{1}H NMR and ^{13}C NMR spectra were recorded on Bruker AM400. Chemical shifts are given in δ relative to tetramethylsilane (TMS), the coupling constants *J* are given in Hz. The spectra were recorded in $CDCl_3$ as solvent at room temperature, $CDCl_3$ served as internal standard (δ = 7.26 ppm for ^{1}H NMR and δ = 77.16 ppm for ^{13}C NMR). Peaks are labeled as singlet (s), doublet (d), triplet (t), quartet (q) and multiplet (m). HPLC was carried out using a Waters 2690 Millenium with photodiode array detector. Optical rotations were recorded on a Perkin Elmer 241 Polarimeter (d = 589 nm, 1 dm cell). High-resolution mass (ESI) were obtained with a Bruker MicroTOF spectrometer. The carbamate protected hydrazines 2 were made according to Litterature procedures.[1]

Typical experimental procedure for the catalytic asymmetric synthesis of 5-hydroxypyrazolidine derivatives 3: The desired nucleophile **2** (0.30 mmol) was added to a stirred solution of aldehyde **1** (0.25 mmol, 1.0 equiv) and catalyst **4a** (0.05 mmol, 20 mol%) in toluene (0.5 mL) at 4 °C. The reaction was vigorously stirred at this temperature for the reported time. Next the crude reaction mixture was directly loaded on and purified by silica gel chromatography (pentane/EtOAc or toluene/EtOAc mixtures) to afford the corresponding pyrazolidine derivative **3**.

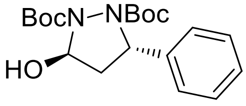

1. A. Bredihhin, U. Mäeorg, *Tetrahedron* **2008**, *64*, 6788.

(3*R*,5*S*)-di-tert-butyl 3-hydroxy-5-phenylpyrazolidine-1,2-dicarboxylate: oil. Rf = 0.23 (Pentane: EtOAc= 3:1); **^{1}H NMR (400MHz, $CDCl_3$):** δ 7.35-7.21 (m, 5H), 5.89 (d, *J* = 5.2 Hz, 1H), 5.39 (t, *J* = 7.6, Hz, 1H), 3.39 (s, 1H), 2.64-2.58 (m, 1H), 2.24-2.19 (m, 1H), 1.51 (s, 9H), 1.42 (s, 9H); **^{13}C NMR (100MHz, $CDCl_3$):** 154.5, 141.8, 128.6, 127.2, 125.9, 82.8, 82.0, 81.6, 62.1, 43.2, 28.3, 28.3; **HRMS (ESI)** : calcd for [M+Na] ($C_{19}H_{28}N_2O_5$) requires m/z 387.1890, found 387.1903; $[\alpha]_D^{25}$ = -6.2 (*c* = 7.5, $CHCl_3$). The enantiomeric excess was determined by HPLC analysis in comparison with authentic racemic material (ODH-column, *n*-hexane/*i*-PrOH = 95/5, λ = 210 nm, 1.0 ml/min) t_r (major enantiomer) = 9.8 min, t_r (minor enantiomer) =5.2 min.

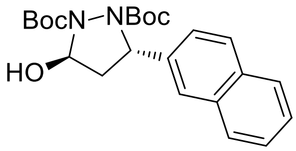

(3*R*,5*S*)-di-tert-butyl 3-hydroxy-5-(naphthalen-2-yl)pyrazolidine-1,2-dicarboxylate: oil. Rf = 0.41 (Pentane: EtOAc= 3:1); **^{1}H NMR (400MHz, $CDCl_3$):** δ 7.85-7.78 (m, 4H), 7.45-7.40 (m, 3H), 5.92 (d, *J* = 5.2 Hz, 1H), 5.57 (t, *J* = 7.6, Hz, 1H), 3.00 (s, 1H), 2.72-2.66 (m, 1H), 2.33-2.30 (m, 1H), 1.53 (s, 9H), 1.44 (s, 9H); **^{13}C NMR (100MHz, $CDCl_3$):** 154.4, 139.1, 133.5, 132.8, 128.6, 128.0, 127.8, 126.3, 125.9, 124.4, 124.3, 82.8, 82.1, 81.7, 62.3, 43.1, 28.4, 28.3; **HRMS (ESI)** : calcd for [M+Na] ($C_{23}H_{30}N_2O_5$) requires m/z 437.2047, found 437.2053; $[\alpha]_D^{25}$ = -0.5 (*c* = 2.0, $CHCl_3$). The enantiomeric excess was determined by HPLC analysis in comparison with authentic racemic material (ODH-column, *n*-hexane/*i*-PrOH = 95/5, λ = 210 nm, 1.0 ml/min) t_r (major enantiomer) = 12.3 min, t_r (minor enantiomer) =6.7 min.

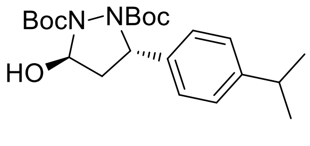

(3*R*,5*S*)-di-tert-butyl 3-hydroxy-5-(4-isopropylphenyl)pyrazolidine-1,2-dicarboxylate: oil. Rf = 0.38 (Pentane: EtOAc= 3:1); **^{1}H NMR (400MHz, $CDCl_3$):** δ 7.27 (d, *J* = 7.2 Hz, 2H), 7.19 (d, *J* = 7.2 Hz, 2H), 5.90 (d, *J* = 5.2 Hz, 1H), 5.41 (t, *J* = 7.2, Hz, 1H), 2.94-2.87 (m, 2H), 2.63-2.57 (m, 1H), 2.32-2.56 (m, 1H), 1.51 (s, 9H), 1.47 (s, 9H), 1.26 (s, 3H), 1.24 (s, 3H); **^{13}C NMR (100MHz, $CDCl_3$):** 156.7, 154.4, 147.9,

139.0, 126.6, 126.5, 125.9, 82.8, 81.9, 81.5, 61.9, 43.0, 33.9, 28.4, 28.3, 24.1, 24.1; **HRMS (ESI)** : calcd for [M+Na] ($C_{22}H_{34}N_2O_5$) requires m/z 429.2360, found 429.2377; $[\alpha]_D^{25}$ = -15.3 (*c* =1.0, $CHCl_3$). The enantiomeric excess was determined by HPLC analysis in comparison with authentic racemic material (ODH-column, *n*-hexane/*i*-PrOH = 95/5, λ = 210 nm, 1.0 ml/min) t_r (major enantiomer) = 7.5 min, t_r (minor enantiomer) = 5.2 min.

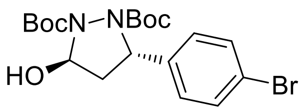

(3*S*,5*R*)-di-tert-butyl 3-(4-bromophenyl)-5-hydroxypyrazolidine-1,2-dicarboxylate: oil. Rf = 0.19 (Pentane: EtOAc= 3:1); **^{1}H NMR (400MHz, $CDCl_3$):** δ 7.43 (d, *J* = 7.2 Hz, 2H), 7.22 (d, *J* = 7.2 Hz, 2H), 5.87 (d, *J* = 5.2 Hz, 1H), 5.32 (t, *J* = 7.6, Hz, 1H), 3.36 (s, 1H), 2.63-2.58 (m, 1H), 2.16-2.11 (m, 1H), 1.52 (s, 9H), 1.42 (s, 9H); **^{13}C NMR (100MHz, $CDCl_3$):** 154.3, 141.0, 131.7, 127.7, 121.1, 82.7, 82.2, 81.8, 61.6, 43.2, 28.3, 28.3; **HRMS (ESI)** : calcd for [M+Na] ($C_{19}H_{27}BrN_2O_5$) requires m/z 465.0996, found 465.0986; $[\alpha]_D^{25}$ = -5.86 (*c* = 6.0, $CHCl_3$). The enantiomeric excess was determined by HPLC analysis in comparison with authentic racemic material (ODH-column, *n*-hexane/*i*-PrOH = 95/5, λ = 210 nm, 1.0 ml/min) t_r (major enantiomer) = 9.7 min, t_r (minor enantiomer) =5.9 min.

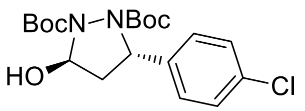

(3*S*,5*R*)-di-tert-butyl 3-(4-chlorophenyl)-5-hydroxypyrazolidine-1,2-dicarboxylate: oil. Rf = 0.20 (Pentane: EtOAc= 3:1); **^{1}H NMR (400MHz, $CDCl_3$):** δ 7.82 (s, 4H), 5.87 (d, *J* = 5.2 Hz, 1H), 5.34 (t, *J* = 7.6, Hz, 1H), 3.24 (s, 1H), 2.64-2.58 (m, 1H), 2.14-2.10 (m, 1H), 1.49 (s, 9H), 1.42 (s, 9H); **^{13}C NMR (100MHz, $CDCl_3$):** 154.3, 140.5, 133.0, 128.8, 127.3, 82.7, 82.2, 81.8, 61.6, 43.2, 28.3, 28.3; **HRMS (ESI)** : calcd for [M+Na] ($C_{19}H_{27}ClN_2O_5$) requires m/z 421.1501, found 421.1503; $[\alpha]_D^{25}$ = -5.87 (c= 3.8, $CHCl_3$). The enantiomeric excess was determined by HPLC analysis in comparison with authentic

racemic material (ODH-column, *n*-hexane/*i*-PrOH = 95/5, λ = 210 nm, 1.0 ml/min) t_r (major enantiomer) = 8.7 min, t_r (minor enantiomer) =5.6 min.

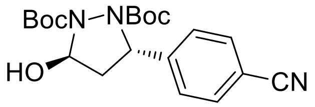

(3*S*,5*R*)-di-tert-butyl 3-(4-cyanophenyl)-5-hydroxypyrazolidine-1,2-dicarboxylate: oil. Rf = 0.54 (Pentane: EtOAc= 1:1); **^{1}H NMR (400MHz, $CDCl_3$):** δ 7.61 (d, *J* = 7.2 Hz, 2H), 7.48 (d, *J* = 7.2 Hz, 2H), 5.89 (d, *J* = 5.2 Hz, 1H), 5.39 (t, *J* = 7.6, Hz, 1H), 3.62 (s, 1H), 2.69-2.64 (m, 1H), 2.06-2.03 (m, 1H), 1.49 (s, 9H), 1.41 (s, 9H); **^{13}C NMR (100MHz, $CDCl_3$):** 154.2, 147.6, 132.6, 126.7, 118.8, 111.2, 82.6, 82.5, 82.1, 61.8, 43.3, 28.3, 28.2; **HRMS (ESI)** : calcd for [M+Na] ($C_{20}H_{27}N_3O_5$) requires m/z 412.1843, found 412.1842; $[\alpha]_D^{25}$ = -5.17 (c= 6.7, $CHCl_3$). The enantiomeric excess was determined by HPLC analysis in comparison with authentic racemic material (ODH-column, *n*-hexane/*i*-PrOH = 95/5, λ = 250 nm, 1.0 ml/min) t_r (major enantiomer) = 14.9 min, t_r (minor enantiomer) =11.1 min.

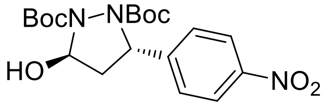

(3*R*,5*S*)-di-tert-butyl 3-hydroxy-5-(4-nitrophenyl)pyrazolidine-1,2-dicarboxylate: oil. Rf = 0.52 (Pentane: EtOAc= 1:1); **^{1}H NMR (400MHz, $CDCl_3$):** δ 8.21-8.18 (m, 2H), 7.50 (d, *J* = 7.2 Hz, 2H), 5.91 (d, *J* = 5.2 Hz, 1H), 5.45 (t, *J* = 7.6, Hz, 1H), 3.14 (s, 1H), 2.73-2.68 (m, 1H), 2.12-2.04 (m, 1H), 1.52 (s, 9H), 1.43 (s, 9H); **^{13}C NMR (100MHz, $CDCl_3$):** 154.2, 149.7, 147.3, 126.8, 124.0, 82.7, 82.6, 82.2, 61.7, 43.4, 28.4, 28.2; **HRMS (ESI)** : calcd for [M+Na] ($C_{19}H_{27}N_3O_7$) requires m/z 432.1741, found 432.1738; $[\alpha]_D^{25}$ = -5.37 (c= 7.5, $CHCl_3$). The enantiomeric excess was determined by HPLC analysis in comparison with authentic racemic material (ODH-column, *n*-hexane/*i*-PrOH = 95/5, λ = 210 nm, 1.0 ml/min) t_r (major enantiomer) = 13.9 min, t_r (minor enantiomer) =10.9 min.

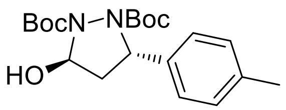

(3*R*,5*S*)-di-tert-butyl 3-hydroxy-5-(p-tolyl)pyrazolidine-1,2-dicarboxylate: oil. Rf = 0.19 (Pentane: EtOAc= 2:1); **^{1}H NMR (400MHz, $CDCl_3$):** δ 7.22 (d, *J* = 7.2 Hz, 2H), 7.12 (d, *J* = 7.2 Hz, 2H), 5.87 (d, *J* = 5.2 Hz, 1H), 5.36 (t, *J* = 7.6, Hz, 1H), 3.20 (s, 1H), 2.61-2.55 (m, 1H), 2.32 (s, 3H), 2.23-2.17 (m, 1H), 1.49 (s, 9H), 1.43 (s, 9H); **^{13}C NMR (100MHz, $CDCl_3$):** 154.4, 138.8, 136.8, 129.2, 125.9, 82.8, 81.9, 81.5, 61.9 43.2, 28.3, 28.3, 21.2; **HRMS (ESI)** : calcd for [M+Na] ($C_{20}H_{30}N_2O_5$) requires m/z 401.2047, found 401.2048; $[\alpha]_D^{25}$ = -10.2 (c=1.0, $CHCl_3$). The enantiomeric excess was determined by HPLC analysis in comparison with authentic racemic material (ODH-column, *n*-hexane/*i*-PrOH = 95/5, λ = 210 nm, 1.0 ml/min) t_r (major enantiomer) = 9.8 min, t_r (minor enantiomer) =5.2 min.

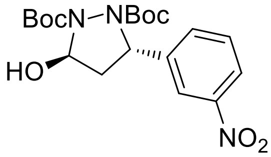

(3*R*,5*S*)-di-tert-butyl 3-hydroxy-5-(3-nitrophenyl)pyrazolidine-1,2-dicarboxylate: oil. Rf = 0.47 (Pentane: EtOAc= 1:1); **^{1}H NMR (400MHz, $CDCl_3$):** δ 8.30 (s, 1H), 8.13-8.11 (m, 1H), 7.65 (d, *J* = 7.6 Hz, 1H), 7.51 (t, *J* = 16 Hz, 1H), 5.93 (d, *J* = 5.2 Hz, 1H), 5.48 (t, *J* = 7.2, Hz, 1H), 3.13 (s, 1H), 2.74-2.68 (m, 1H), 2.16-2.09 (m, 1H), 1.55 (s, 9H), 1.44 (s, 9H); **^{13}C NMR (100MHz, $CDCl_3$):** 154.3, 148.8, 144.6, 132.3, 129.7, 122.5, 120.7, 82.8, 82.5, 82.2, 61.6, 43.5, 28.4, 28.2, 28.2; **HRMS (ESI)** : calcd for [M+Na] ($C_{19}H_{27}N_3O_7$) requires m/z 432.1741, found 432.1746; $[\alpha]_D^{25}$ = -9.8 (c 1.0, $CHCl_3$). The enantiomeric excess was determined by HPLC analysis in comparison with authentic racemic material (ODH-column, *n*-hexane/*i*-PrOH = 95/5, λ = 250 nm, 1.0 ml/min) t_r (major enantiomer) = 16.7 min, t_r (minor enantiomer) =9.5 min.

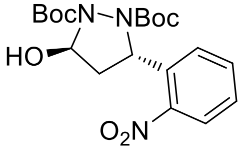

(3*R*,5*S*)-di-tert-butyl 3-hydroxy-5-(2-nitrophenyl)pyrazolidine-1,2-dicarboxylate: oil. Rf = 0.34 (Pentane: EtOAc= 3:1); **^{1}H NMR (400MHz, $CDCl_3$):** δ 8.01 (d, *J* = 6.0 Hz, 1H), 7.96 (d, *J* = 6.0 Hz, 1H), 7.61 (t, *J* = 5.6 Hz, 1H), 7.42 (t, *J* = 6.4 Hz, 1H), 5.94 (bs, 1H), 5.88 (d, *J* = 3.6, Hz, 1H), 3.00-2.96 (m, 2H), 2.02-1.97 (m, 1H), 1.55 (s, 9H), 1.55 (s, 9H); **^{13}C NMR (100MHz, $CDCl_3$):** 153.5, 147.7, 138.3, 134.0, 128.3, 128.1, 125.1, 82.5, 82.3, 82.2, 60.3, 43.3, 28.4, 28.2; **HRMS (ESI)** : calcd for [M+Na] ($C_{19}H_{27}N_3O_7$) requires m/z 432.1741, found 432.1750; $[\alpha]_D^{25}$ = 32.4 (*c*=1.0, $CHCl_3$). The enantiomeric excess was determined by HPLC analysis in comparison with authentic racemic material (AD-column, *n*-hexane/*i*-PrOH = 90/10, λ = 210 nm, 1.0 ml/min) t_r (major enantiomer) = 7.8 min, t_r (minor enantiomer) =5.3 min.

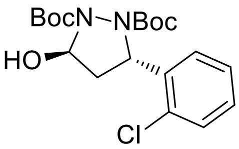

(3*S*,5*R*)-di-tert-butyl 3-(2-chlorophenyl)-5-hydroxypyrazolidine-1,2-dicarboxylate: oil. Rf = 0.5 (Pentane: EtOAc= 3:1); **^{1}H NMR (400MHz, $CDCl_3$):** δ 7.67 (d, *J* = 6.04Hz, 1H), 7.33 (d, *J* = 6.0 Hz, 1H), 7.23 (t, *J* = 6.0 Hz, 1H), 7.18 (t, *J* = 6.4 Hz, 1H), 5.90 (d, *J* = 4, Hz, 1H), 5.66 (t, *J* = 6.4 Hz, 1H), 3.40 (bs, 1H), 2.88-2.84 (m, 1H), 1.99- 1.94 (m, 1H), 1.54 (s, 9H), 1.41 (s, 9H); **^{13}C NMR (100MHz, $CDCl_3$):** 154.0, 140.0, 132.0, 129.6, 128.5, 127.1, 126.8, 82.4, 82.3, 81.8, 60.5, 42.2, 28.4, 28.2; **HRMS (ESI)** : calcd for [M+Na] ($C_{19}H_{27}N_2ClO_5$) requires m/z 421.1501, found 421.1511; $[\alpha]_D^{25}$ = -8.9 (*c*=1.0, $CHCl_3$). The enantiomeric excess was determined by HPLC analysis in comparison with authentic racemic material after reduction to the corresponding alcohol (AD-column, *n*-hexane/*i*-PrOH = 90/10, λ = 210 nm, 1.0 ml/min) t_r (major enantiomer) = 23.2 min, t_r (minor enantiomer) =29.9 min.

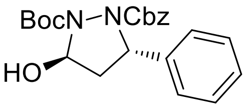

A

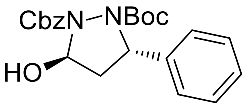

B

(3*R*,5*S*)-1-benzyl 2-tert-butyl 3-hydroxy-5-phenylpyrazolidine-1,2-dicarboxylate and **(3*S*,5*R*)-1-benzyl 2-tert-butyl 5-hydroxy-3-phenylpyrazolidine-1,2-dicarboxylate**: oil. **^{1}H NMR (400MHz, $CDCl_3$): (**A:B = 58:42 ratio**)** δ 7.39-7.25 (m, 18H, A+B), 5.98 (d, *J* = 4.4 Hz, 0.7H, B), 5.94 (d, *J* = 4.0 Hz, 1H, A), 5.52 (t, *J*= 7.2 Hz, 1H, A), 5.43 (t, *J*= 7.2 Hz, 0.7H, B), 5.30-5.15 (m, 3.7H, A+B), 3.46 (bs, 1H, A), 3.37 (bs, 0.6H, B), 2.70-2.62 (m, 1.7H, A+B), 2.32-2.19 (m, 1.7H, A+B), 1.90 (bs, 1H, A), 1.43 (s, 9H, A), 1.41 (s, 7H, B); **^{13}C NMR (100MHz, $CDCl_3$):** 157.7, 156.9, 155.1, 154.4, 141.5, 141.1, 136.2, 135.9, 128.7, 128.6, 128.4, 128.2, 128.1, 127.8, 127.5, 127.3, 126.0, 125.9, 83.1, 82.9, 82.4, 82.1, 68.1, 68.0, 62.4, 43.2, 43.0, 28.2; **HRMS (ESI)** : calcd for [M+Na] ($C_{22}H_{26}N2O_5$) requires m/z 421.1734, found 421.1740; The enantiomeric excess was determined by HPLC analysis in comparison with authentic racemic material (ODH-column, *n*-hexane/*i*-PrOH = 95/5, λ = 210 nm, 1.0 ml/min) t_{rB} (minor enantiomer) = 10.2 min, t_{rB} (major enantiomer) =15.4 min, t_{rA} (major enantiomer) =17.6 min, t_{rA} (minor enantiomer) = 24.8min.

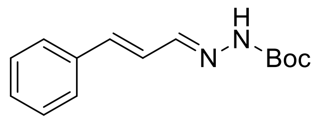

(*E*)-tert-butyl 2-((*E*)-3-phenylallylidene)hydrazinecarboxylate: oil.; **^{1}H NMR (400MHz, $CDCl_3$):** δ 8.26 (bs, 1H), 7.70 (d, *J* = 8.8 Hz, 1H), 7.42 (d, *J* = 7.2 Hz, 2H), 7.34-7.28 (m, 3H), 7.04-6.98 (m, 1H), 6.79 (d, *J* = 16.0 Hz, 1H), 1.55 (s, 9H); **^{13}C NMR (100MHz, $CDCl_3$):** 145.6, 138.1, 136.0, 128.8, 128.8, 126.9, 125.4, 28.4; **HRMS (ESI)** : calcd for [M+Na] ($C_{14}H_{18}N_2O_2$) requires m/z 269.1260, found 269.1263.

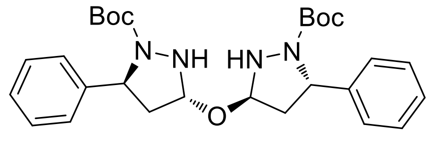

(3*R*,3'*R*,5*S*,5'*S*)-di-tert-butyl 3,3'-oxybis(5-phenylpyrazolidine-1-carboxylate): oil. **^{1}H NMR (400MHz, $CDCl_3$):** δ 7.44-7.40 (m, 4H), 7.35 (t, *J* = 6.0 Hz, 2H), 7.26-7.24 (m, 4H), 5.83 (bs, 1H), 5.53-5.14 (m, 1H), 4.90 (bs, 1H), 4.73 (bs, 1H), 4.59-4.57 (m, 1H),

4.31 (bs, 1H), 2.83-2.78 (m, 1H), 2.73-2.67 (m, 1H), 2.54-2.49 (m, 1H), 2.19-2.15 (m, 1H),1.62 (bs, 2H), 1.54 (s, 9H), 1.45 (s, 9H); **^{13}C NMR (100MHz, $CDCl_3$):** 155.8, 155.3, 142.7, 139.1, 128.7, 128.7, 128.2, 128.2, 127.3, 126.4, 84.3, 82.0, 81.5, 79.5, 65.4, 64.8, 44.4, 41.7, 29.8, 28.6, 28.5; **HRMS (ESI)** : calcd for [M+Na] ($C_{28}H_{38}N4O_5$) requires m/z 533.2734, found 533.2723; $[\alpha]_D^{25}$ = -15.5 (c = 0.2, $CHCl_3$).

Synthesis of (*S*)-di-tert-butyl 3-phenylpyrazolidine-1,2-dicarboxylate:

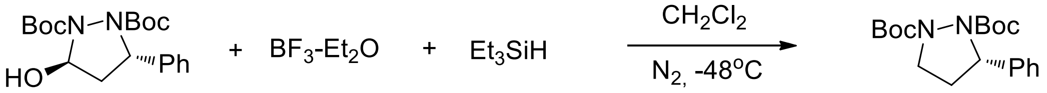

To a solution of (3*R*,5*S*)-di-tert-butyl 3-hydroxy-5-phenylpyrazolidine-1,2-dicarboxylate (58 mg, 0.16 mmol) and triethyl silane (24 μL, 0.16 mmol) in anhydrous DCM (4.8 mL) was added $BF_3 * Et_2O$ (22 μL, 0.17 mmol) at -48 °C. After stirred at -48 °C for 1 hour the reaction was quenched with some drops of aqueous $NaHCO_3$, extracted with CH_2Cl_2 and dried over Na_2SO_4 . The residue was purified by flash chromatography (Pentane:EtOAc-6:1 to 3:1) on silica gel to give the pure product (68% yield).

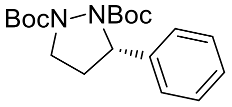

(*S*)-di-tert-butyl 3-phenylpyrazolidine-1,2-dicarboxylate: oil. Rf = 0.52 (Pentane: EtOAc= 3:1); **^{1}H NMR (400MHz, $CDCl_3$):** δ 7.37-7.21 (m, 5H), 5.21 (t, *J* = 7.2, Hz, 1H), 4.00 (t, *J* = 7.2, Hz, 1H), 3.32-3.25 (m, 1H), 2.56-2.48 (m, 1H), 2.13-2.04 (m, 1H), 1.49 (s, 9H), 1.44 (s, 9H); **^{13}C NMR (100MHz, $CDCl_3$):** 156.4, 141.8, 128.6, 127.2, 126.0, 81.3, 81.3, 62.5, 46.8, 35.6, 28.4, 28.3; **HRMS (ESI)** : calcd for [M+Na] ($C_{19}H_{28}N_2O_4$) requires m/z 371.1941, found 371.1958; $[\alpha]_D^{25}$ = +2.2 (c= 1.0, $CHCl_3$).

(3*R*,5*S*)-di-tert-butyl 3-allyl-5-phenylpyrazolidine-1,2-dicarboxylate:

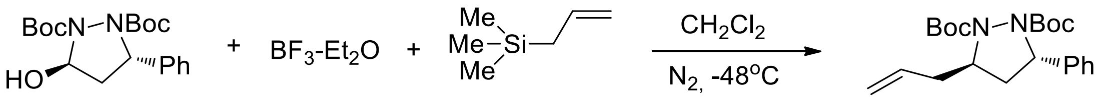

To a solution of (3*R*,5*S*)-di-tert-butyl 3-hydroxy-5-phenylpyrazolidine-1,2-dicarboxylate (58 mg, 0.16 mmol) and allyltrimethylsylane (24 µL, 0.16 mmol) in anhydrous CH_2Cl_2 (4.8 mL) was added $BF_3 * Et_2O$ (27 µL, 0.17 mmol) at -48 °C. After stirring at -48 °C for 1 hour the reaction was quenched with some drops of aqueous $NaHCO_3$, extracted with CH_2Cl_2 and dried over Na_2SO_4. The residue was purified by flash chromatography on silica gel ((Pentane:EtOAc-6:1)to give the pure product (57% yield).

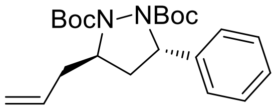

(3*R*,5*S*)-di-tert-butyl 3-allyl-5-phenylpyrazolidine-1,2-dicarboxylate: oil. Rf = 0.61 (Pentane: EtOAc= 5:1); **^{1}H NMR (400MHz, $CDCl_3$):** δ 7.36-7.20 (m, 5H), 5.86-5.79 (m, 1H), 5.30 (t, *J* = 7.2Hz, 1H), 5.16-5.09 (m, 2H), 4.31 (s, 1H), 2.37-2.30 (m, 2H), 2.26-2.20 (m, 2H), 1.46 (s, 18H),; **^{13}C NMR (100MHz, $CDCl_3$):** 141.6, 134.3, 128.6, 127.2, 126.0, 117.9, 81.2, 81.1, 61.8, 58.3, 39.6, 38.1, 28.4; **HRMS (ESI)** : calcd for [M+Na] ($C_{22}H_{32}N_2O_4$) requires m/z 411.2254, found 411.2238; $[\alpha]_D^{25}$ = -26.3 (*c*= 1.0, $CHCl_3$).

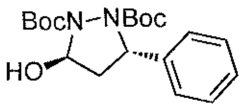
BocN–NBoc
HO

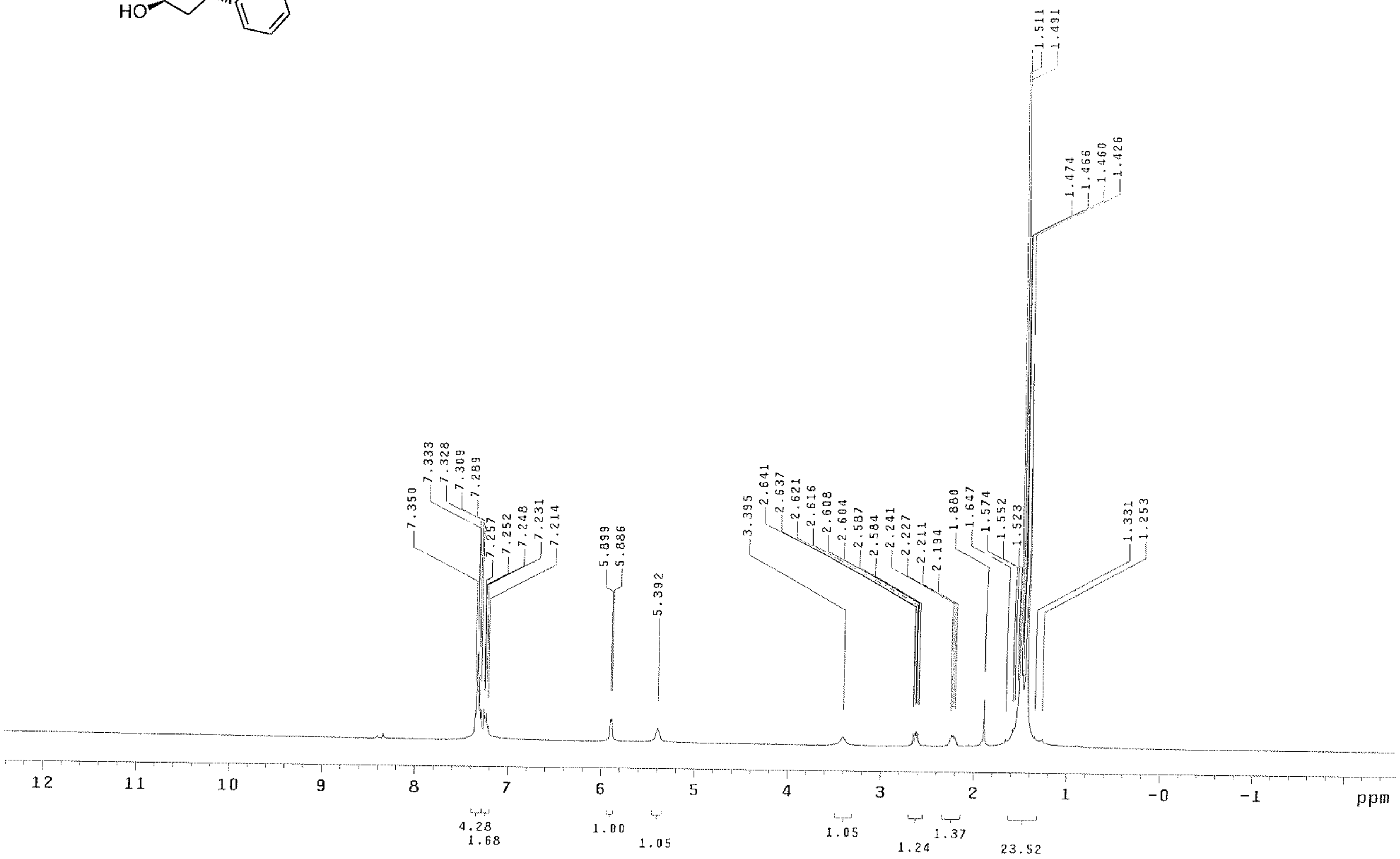
7.350
7.333
7.328
7.309
7.289
7.257
7.252
7.248
7.231
7.214
5.899
5.886
5.392
3.395
2.641
2.637
2.621
2.616
2.608
2.604
2.587
2.584
2.241
2.227
2.211
2.194
1.880
1.647
1.574
1.552
1.523
1.511
1.491
1.474
1.466
1.460
1.426
1.331
1.253
12
11
10
9
8
7
6
5
4
3
2
1
-0
-1
ppm
4.28
1.68
1.00
1.05
1.05
1.24
1.37
23.52

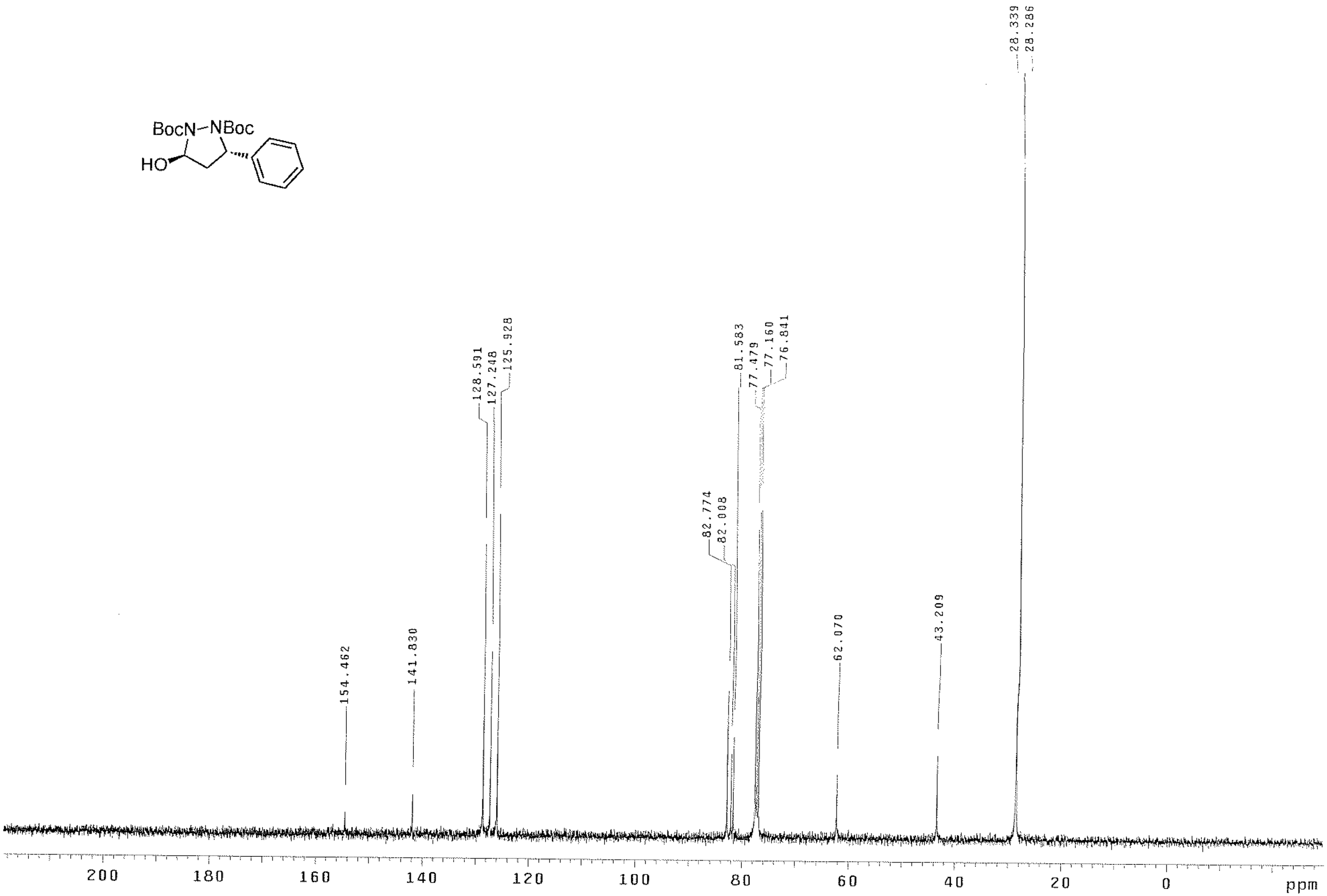

BocN–NBoc
HO
28.339
28.286
43.209
62.070
76.841
77.160
77.479
81.583
82.008
82.774
125.928
127.248
128.591
141.830
154.462
200
180
160
140
120
100
80
60
40
20
0
ppm

Display Report

Analysis Info

Analysis Name	H:\Data2\Luca\ld555000001.d	Acquisition Date	2011-03-01 14:19:40
Method	tune_low_dirk.m	Operator	pia
Sample Name	ld555	Instrument / Ser#	micrOTOF 125
Comment			

Acquisition Parameter

Source Type	ESI	Ion Polarity	Positive	Set Nebulizer	0.4 Bar
Focus	Not active			Set Dry Heater	170 °C
Scan Begin	50 m/z	Set Capillary	4500 V	Set Dry Gas	4.0 l/min
Scan End	3000 m/z	Set End Plate Offset	-500 V	Set Divert Valve	Source

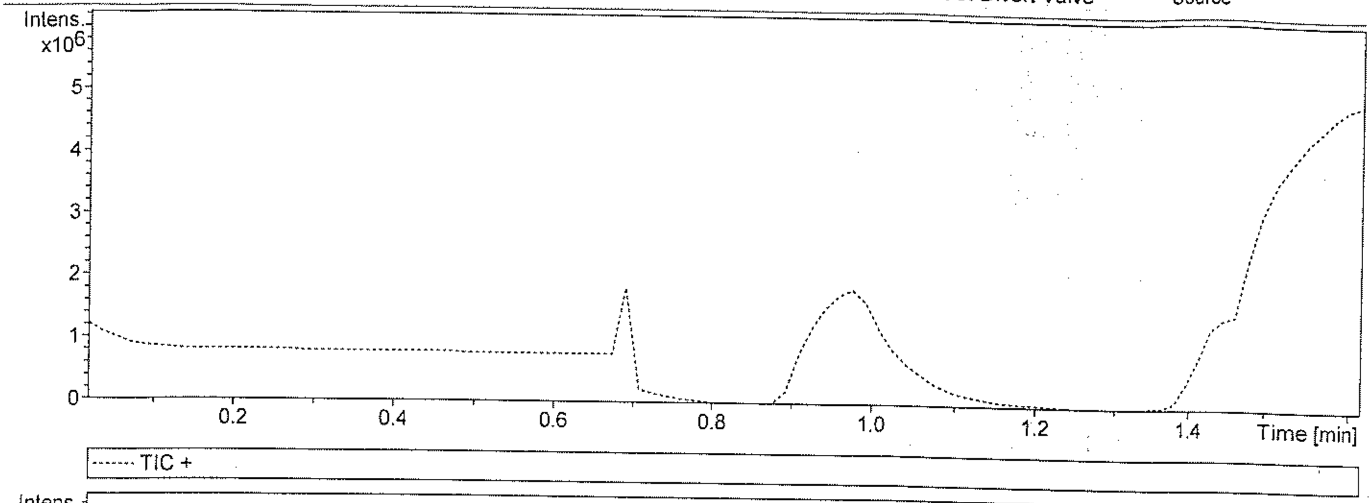

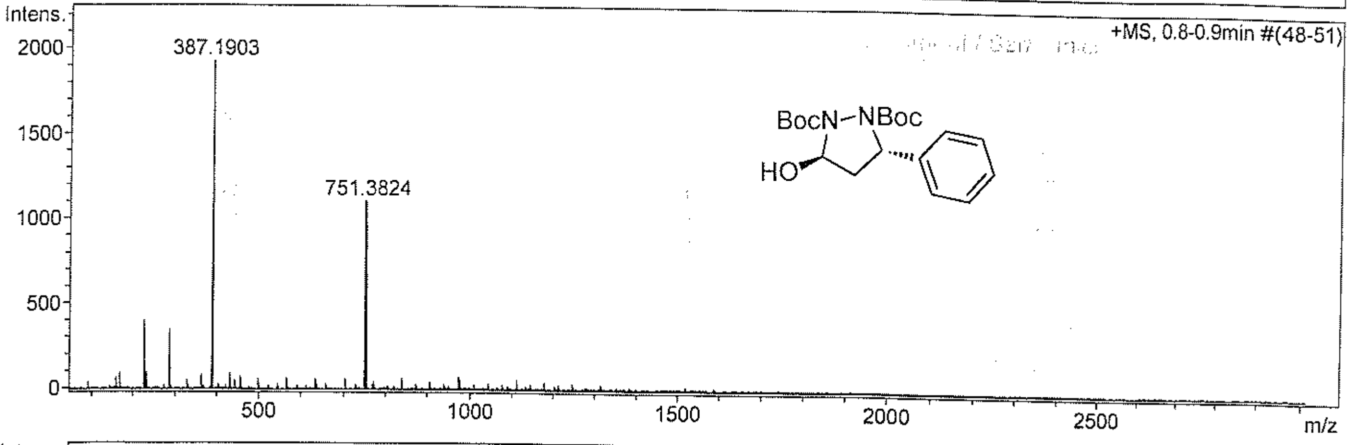

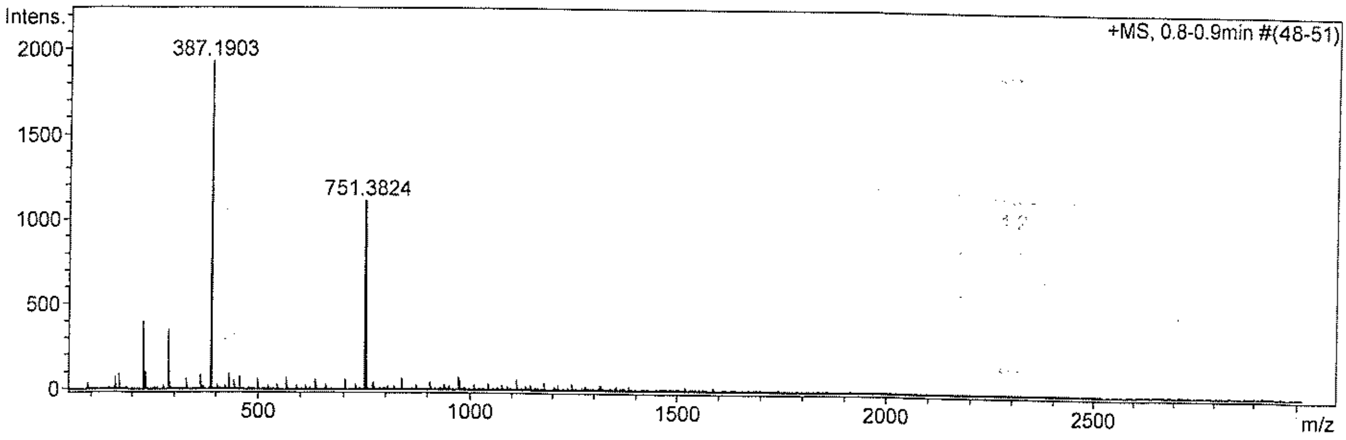

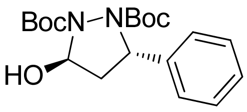
BocN
NBoc
HO

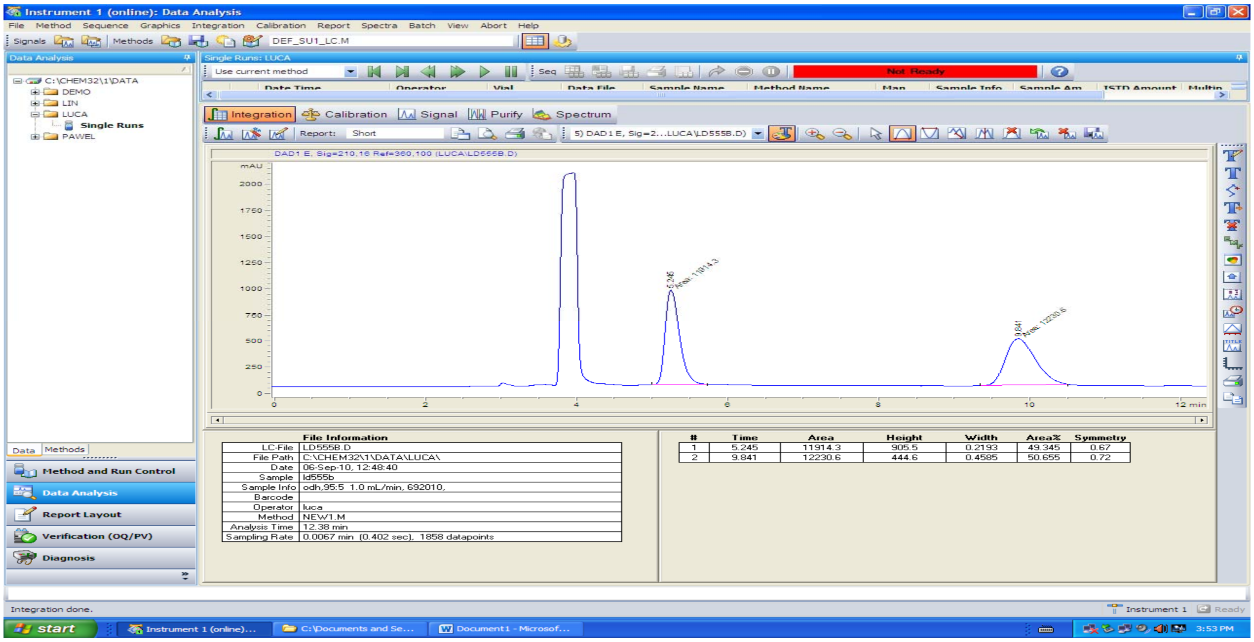
Instrument 1 (online): Data Analysis
DAD1 E, Sig=210,16 Ref=360,100 (LUCA\LD555B.D)
5.245 Area: 11914.3
9.841 Area: 12230.6
File Information
LC-File LD555B.D
File Path C:\CHEM32\1\DATA\LUCA\
Date 06-Sep-10, 12:48:40
Sample ld555b
Sample Info odh,95:5 1.0 mL/min, 692010,
Operator luca
Method NEW1.M
Analysis Time 12.38 min
Sampling Rate 0.0067 min (0.402 sec), 1858 datapoints
Time Area Height Width Area% Symmetry
1 5.245 11914.3 905.5 0.2193 49.345 0.67
2 9.841 12230.6 444.6 0.4585 50.655 0.72
Integration done.

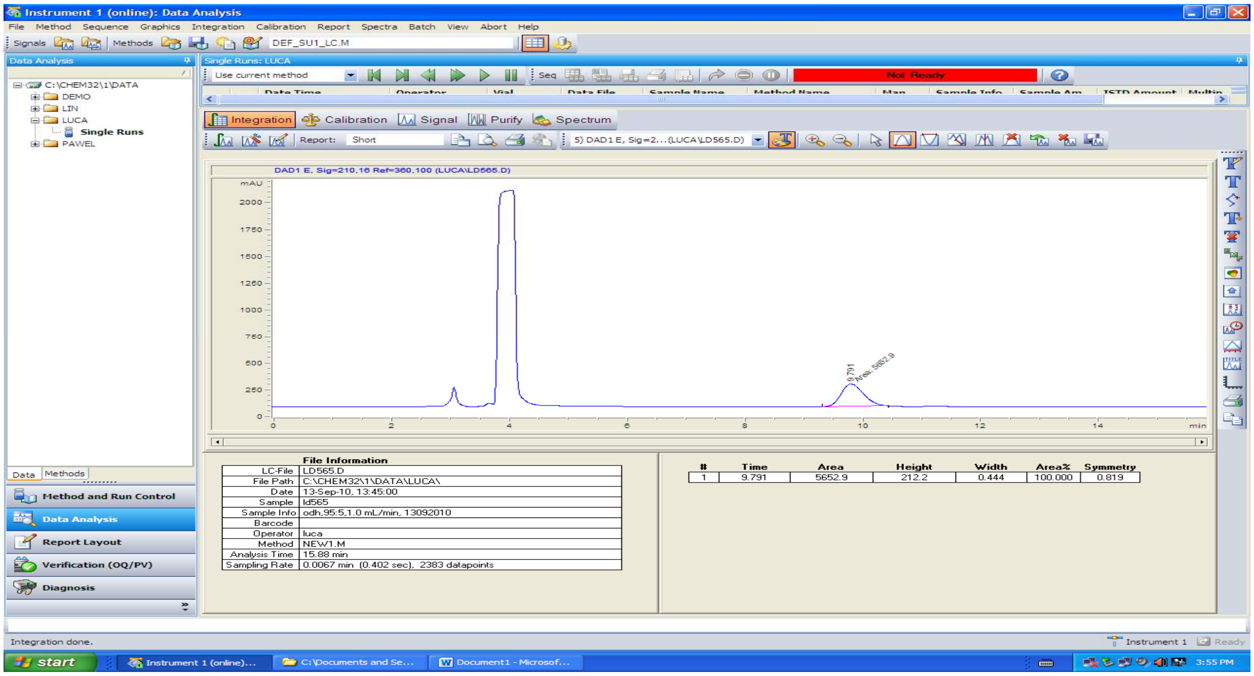
Instrument 1 (online): Data Analysis
DAD1 E, Sig=210,16 Ref=360,100 (LUCA\LD565.D)
9.791 Area: 5652.9
File Information
LC-File LD565.D
File Path C:\CHEM32\1\DATA\LUCA\
Date 13-Sep-10, 13:45:00
Sample ld565
Sample Info odh,95:5,1.0 mL/min, 13092010
Operator luca
Method NEW1.M
Analysis Time 15.88 min
Sampling Rate 0.0067 min (0.402 sec), 2383 datapoints
Time Area Height Width Area% Symmetry
1 9.791 5652.9 212.2 0.444 100.000 0.819
Integration done.

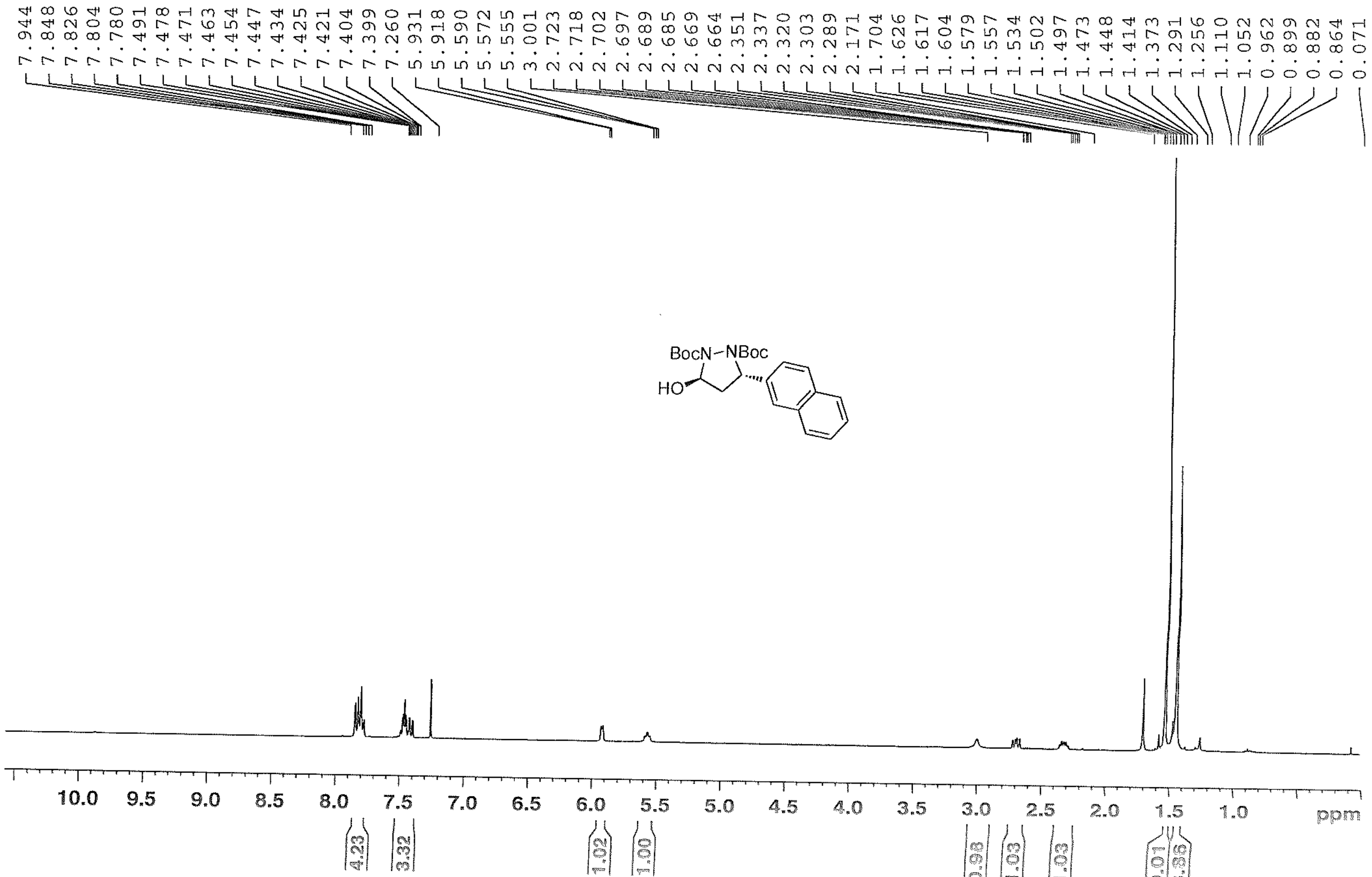
7.944
7.848
7.826
7.804
7.780
7.491
7.478
7.471
7.463
7.454
7.447
7.434
7.425
7.421
7.404
7.399
7.260
5.931
5.918
5.590
5.572
5.555
3.001
2.723
2.718
2.702
2.697
2.689
2.685
2.669
2.664
2.351
2.337
2.320
2.303
2.289
2.171
1.704
1.626
1.617
1.604
1.579
1.557
1.534
1.502
1.497
1.473
1.448
1.414
1.373
1.291
1.256
1.110
1.052
0.962
0.899
0.882
0.864
0.071
BocN–NBoc
HO
10.0
9.5
9.0
8.5
8.0
7.5
7.0
6.5
6.0
5.5
5.0
4.5
4.0
3.5
3.0
2.5
2.0
1.5
1.0
ppm
4.23
3.32
1.02
1.00
0.98
1.03
1.03
9.01
8.86

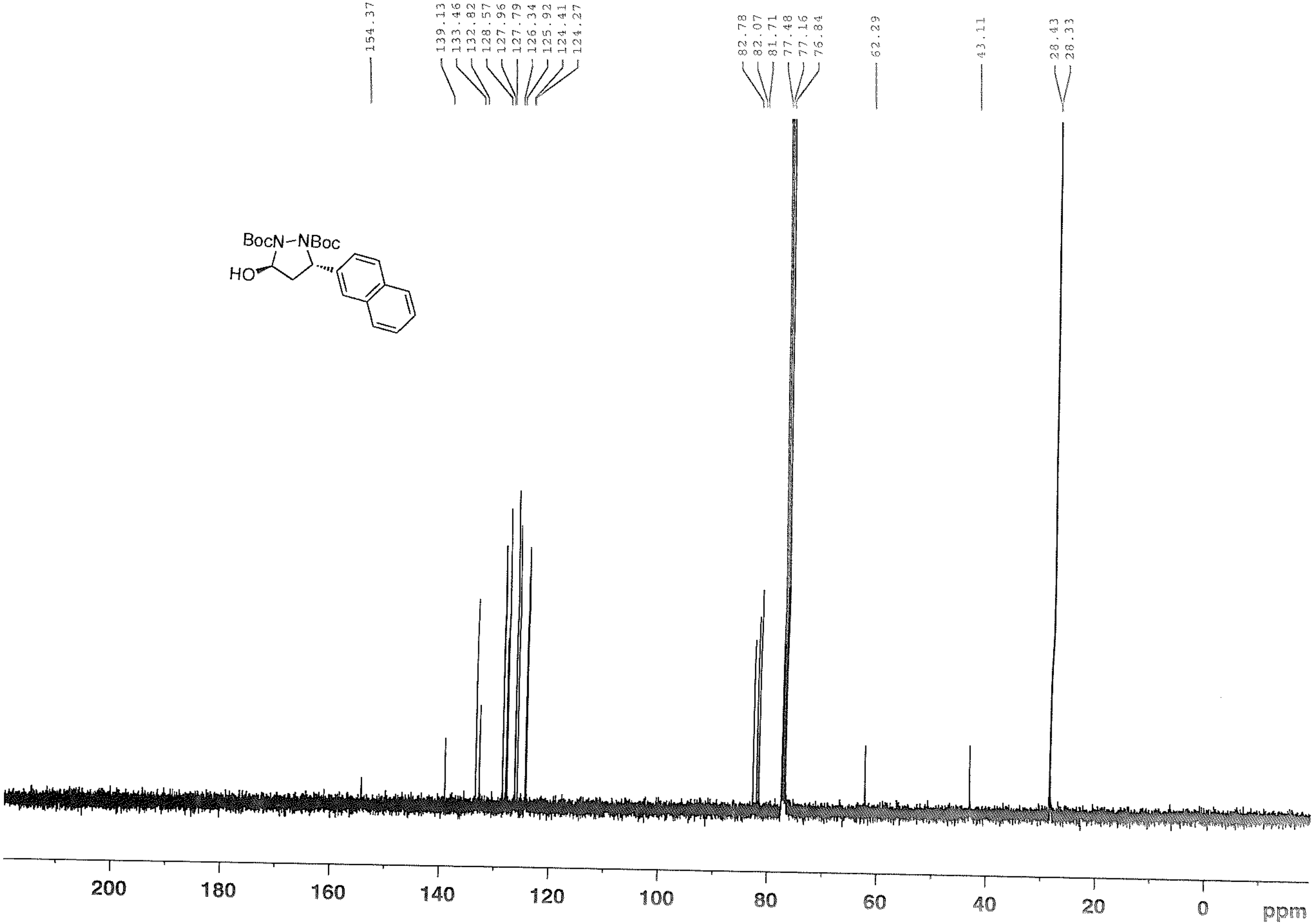
BocN–NBoc
HO
154.37
139.13
133.46
132.82
128.57
127.96
127.79
126.34
125.92
124.41
124.27
82.78
82.07
81.71
77.48
77.16
76.84
62.29
43.11
28.43
28.33
200
180
160
140
120
100
80
60
40
20
0
ppm

Display Report

Analysis Info

Analysis Name	H:\Data2\Luca\ld586000002.d	Acquisition Date	2011-03-03 15:12:57
Method	tune_low_dirk.m	Operator	pia
Sample Name	ld586	Instrument / Ser#	micrOTOF 125
Comment			

Acquisition Parameter

Source Type	ESI	Ion Polarity	Positive	Set Nebulizer	0.4 Bar
Focus	Not active			Set Dry Heater	170 °C
Scan Begin	50 m/z	Set Capillary	4500 V	Set Dry Gas	4.0 l/min
Scan End	3000 m/z	Set End Plate Offset	-500 V	Set Divert Valve	Source

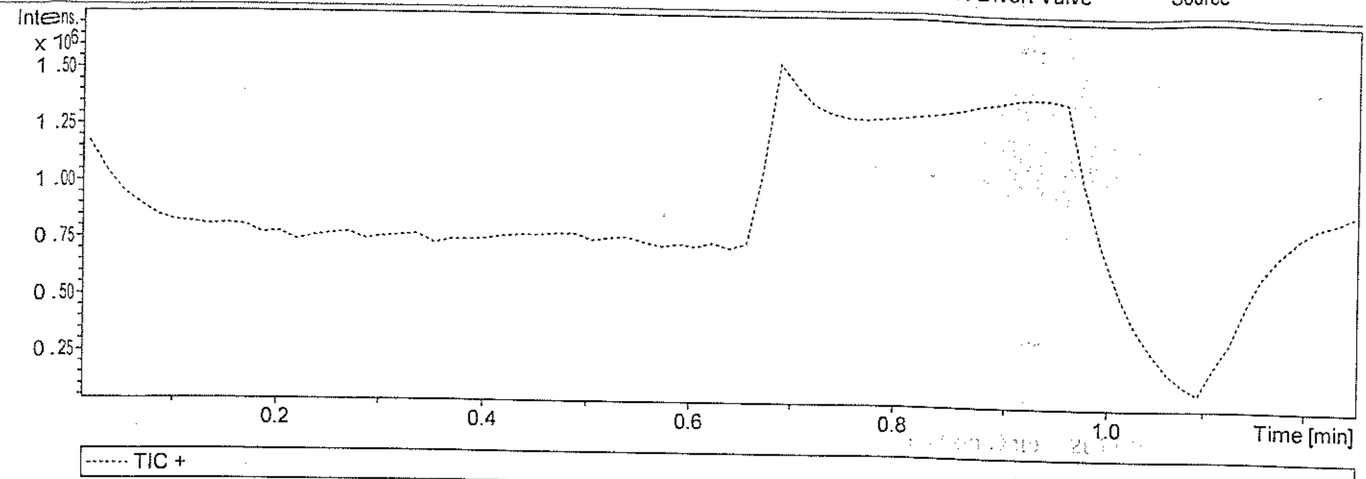

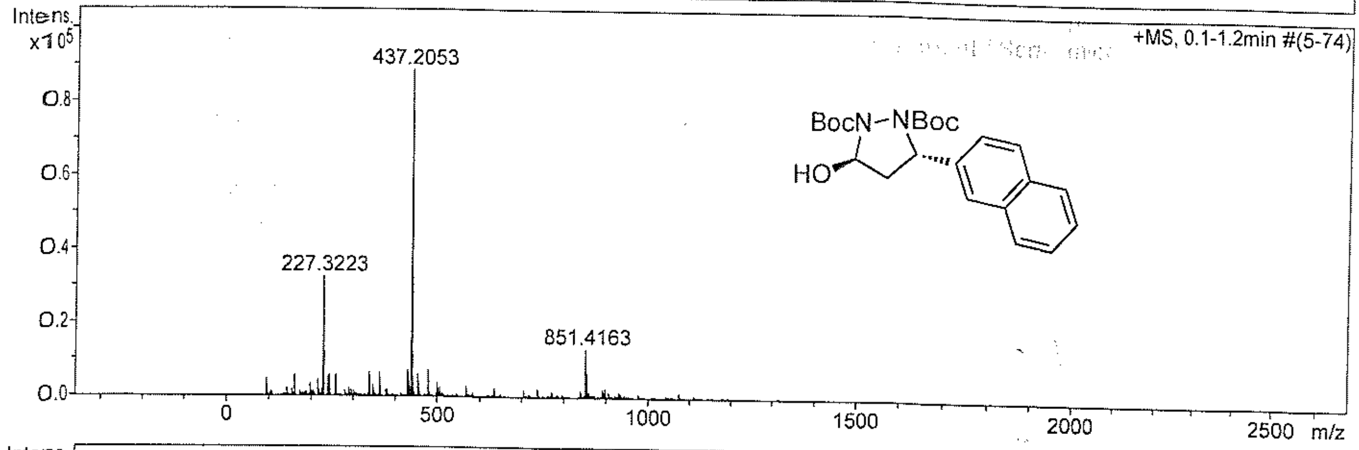

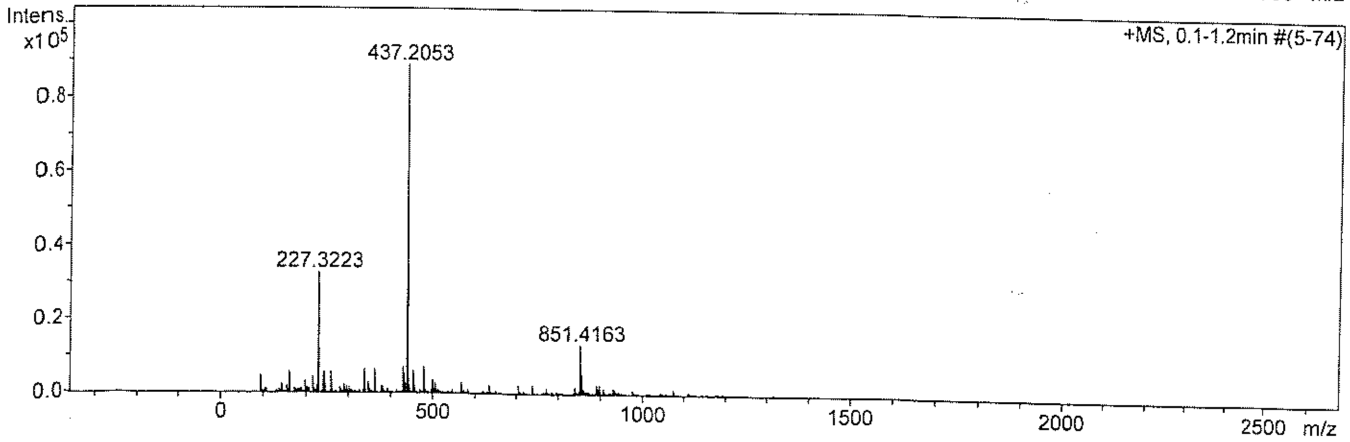

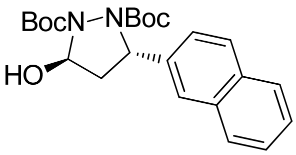
BocN
NBoc
HO

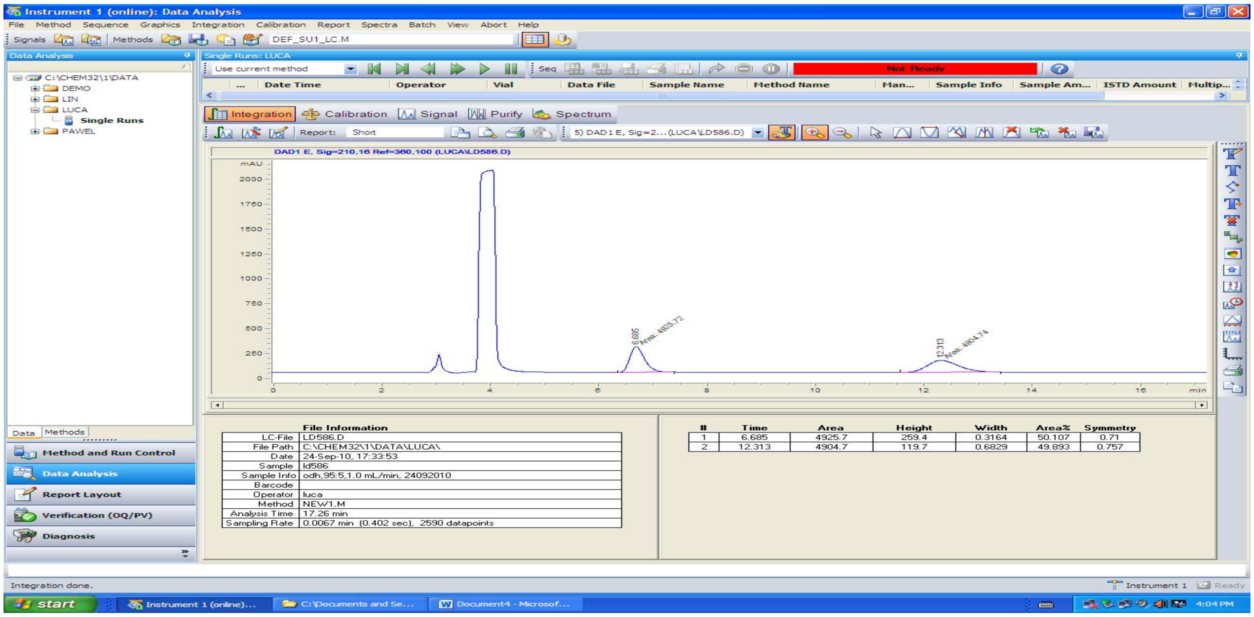
Instrument 1 (online): Data Analysis
DAD1 E, Sig=210,16 Ref=360,100 (LUCA\LD586.D)
mAU
min
6.685
Area: 4925.72
12.313
Area: 4904.74
File Information
LC-File LD586.D
File Path C:\CHEM32\1\DATA\LUCA\
Date 24-Sep-10, 17:33:53
Sample ld586
Sample Info odh,95:5,1.0 mL/min, 24092010
Barcode
Operator luca
Method NEW1.M
Analysis Time 17.26 min
Sampling Rate 0.0067 min (0.402 sec), 2590 datapoints
Time Area Height Width Area% Symmetry
1 6.685 4925.7 259.4 0.3164 50.107 0.71
2 12.313 4904.7 119.7 0.6829 49.893 0.757
Integration done.

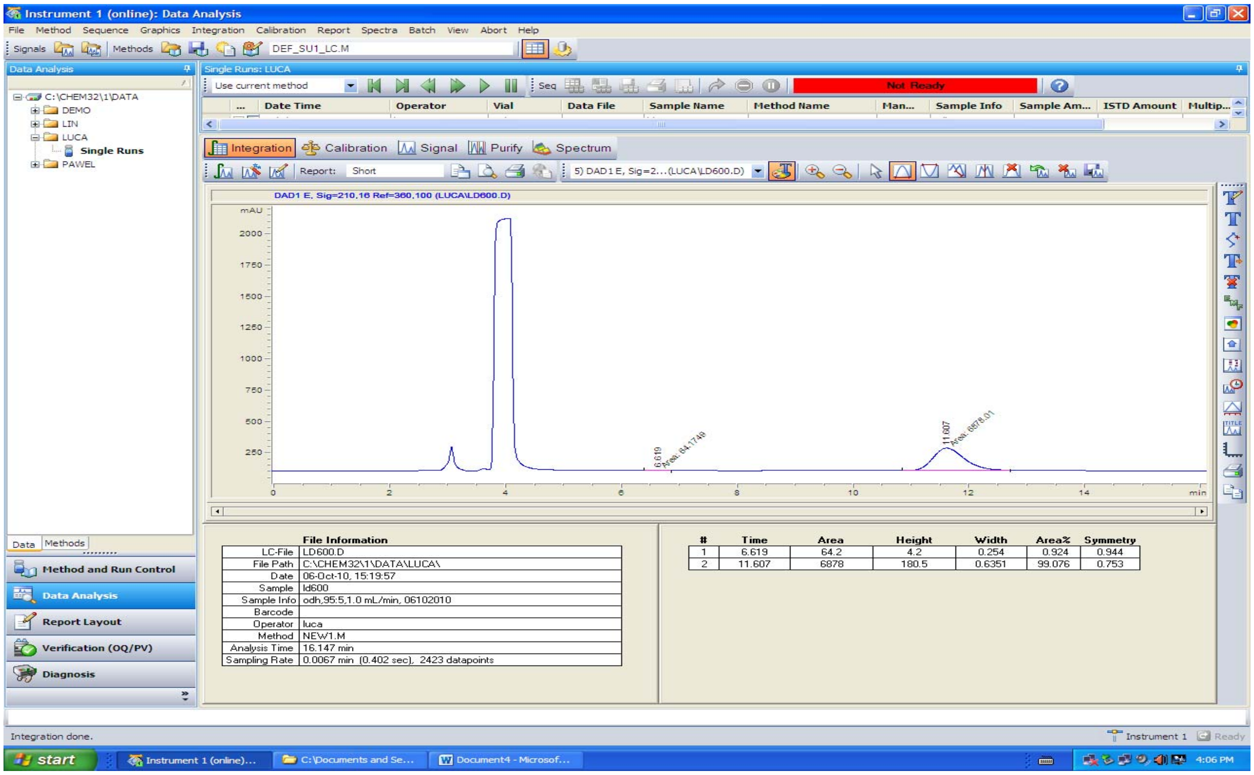
Instrument 1 (online): Data Analysis
DAD1 E, Sig=210,16 Ref=360,100 (LUCA\LD600.D)
mAU
min
6.619
Area: 64.1749
11.607
Area: 6878.01
File Information
LC-File LD600.D
File Path C:\CHEM32\1\DATA\LUCA\
Date 06-Oct-10, 15:19:57
Sample ld600
Sample Info odh,95:5,1.0 mL/min, 06102010
Barcode
Operator luca
Method NEW1.M
Analysis Time 16.147 min
Sampling Rate 0.0067 min (0.402 sec), 2423 datapoints
Time Area Height Width Area% Symmetry
1 6.619 64.2 4.2 0.254 0.924 0.944
2 11.607 6878 180.5 0.6351 99.076 0.753
Integration done.

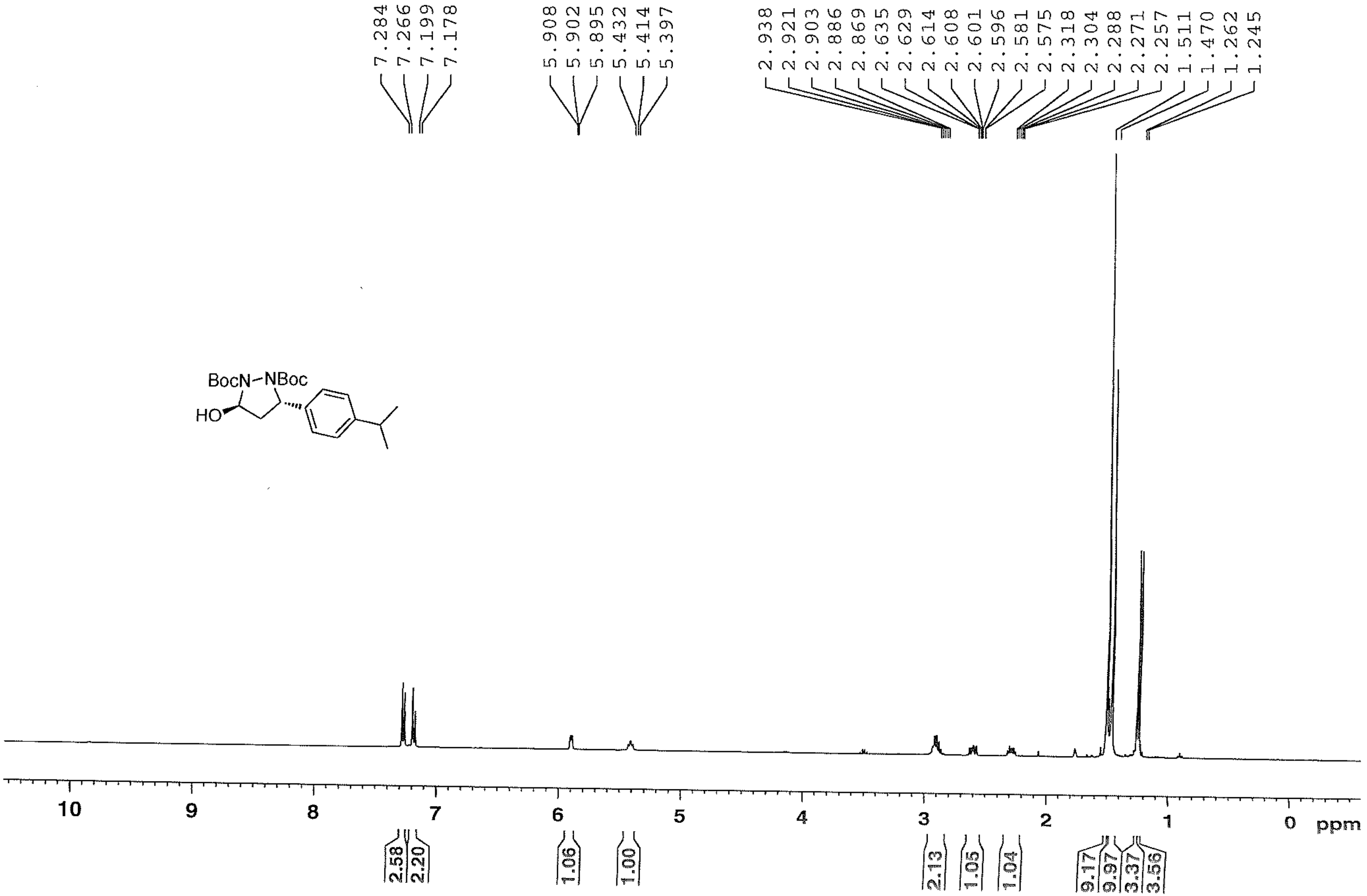
7.284
7.266
7.199
7.178
5.908
5.902
5.895
5.432
5.414
5.397
2.938
2.921
2.903
2.886
2.869
2.635
2.629
2.614
2.608
2.601
2.596
2.581
2.575
2.318
2.304
2.288
2.271
2.257
1.511
1.470
1.262
1.245
BocN
NBoc
HO
10
9
8
7
6
5
4
3
2
1
0
ppm
2.58
2.20
1.06
1.00
2.13
1.05
1.04
9.17
9.97
3.37
3.56

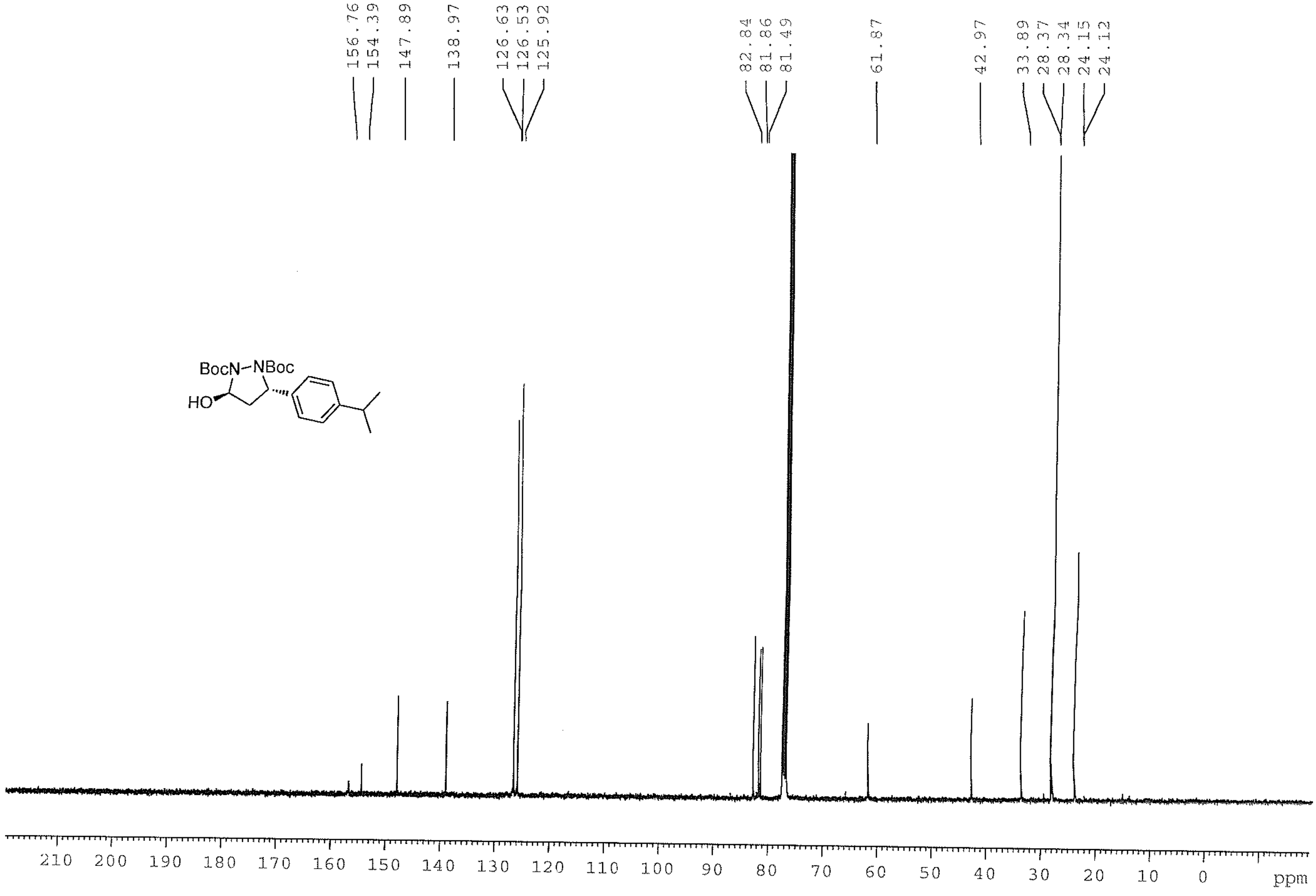
156.76
154.39
147.89
138.97
126.63
126.53
125.92
82.84
81.86
81.49
61.87
42.97
33.89
28.37
28.34
24.15
24.12
BocN–NBoc
HO
210 200 190 180 170 160 150 140 130 120 110 100 90 80 70 60 50 40 30 20 10 0
ppm

Display Report

Analysis Info

Analysis Name	H:\Data2\Luca\dobBocIsop000001.d	Acquisition Date	2011-03-07 12:34:47
Method	tune_low_dirk.m		
Sample Name	dobBocIsop	Operator	pia
Comment		Instrument / Ser#	micrOTOF 125

Acquisition Parameter

Source Type	ESI	Ion Polarity	Positive	Set Nebulizer	0.4 Bar
Focus	Not active			Set Dry Heater	170 °C
Scan Begin	50 m/z	Set Capillary	4500 V	Set Dry Gas	4.0 l/min
Scan End	3000 m/z	Set End Plate Offset	-500 V	Set Divert Valve	Source

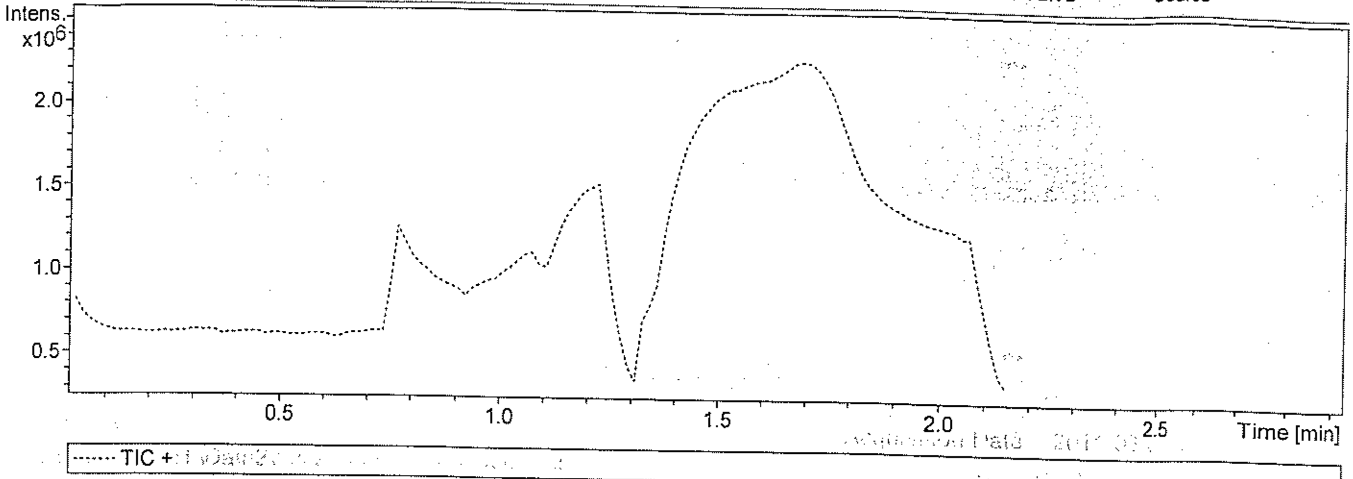

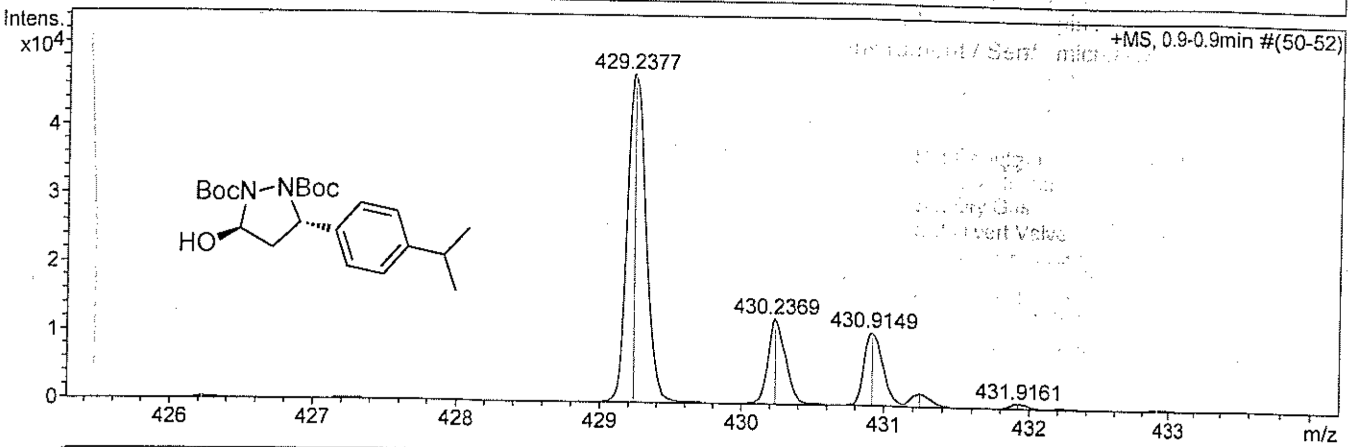

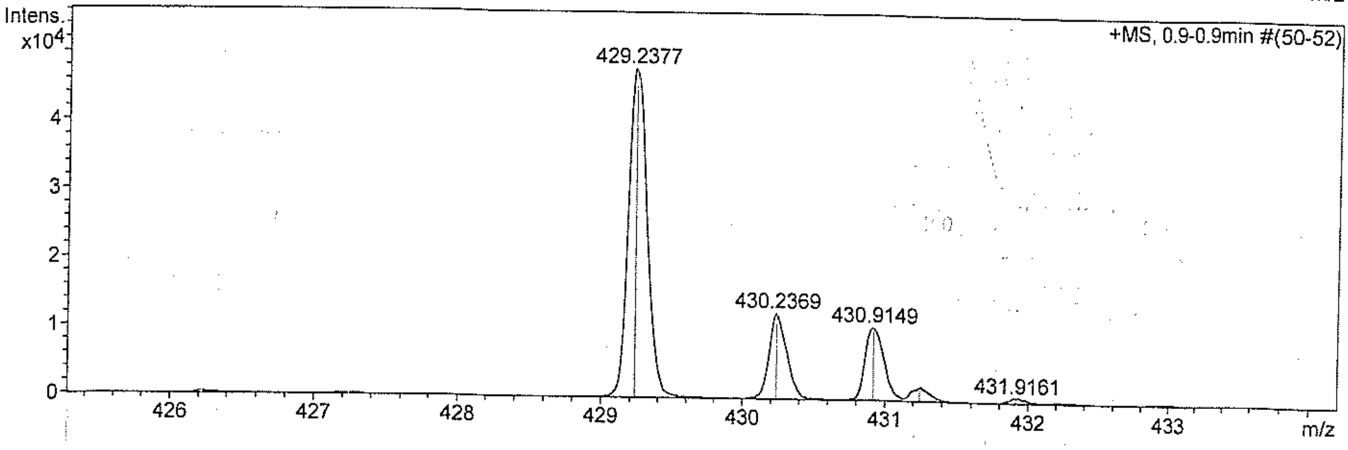

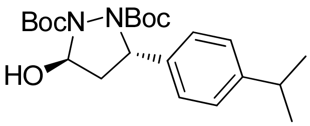
BocN
NBoc
HO

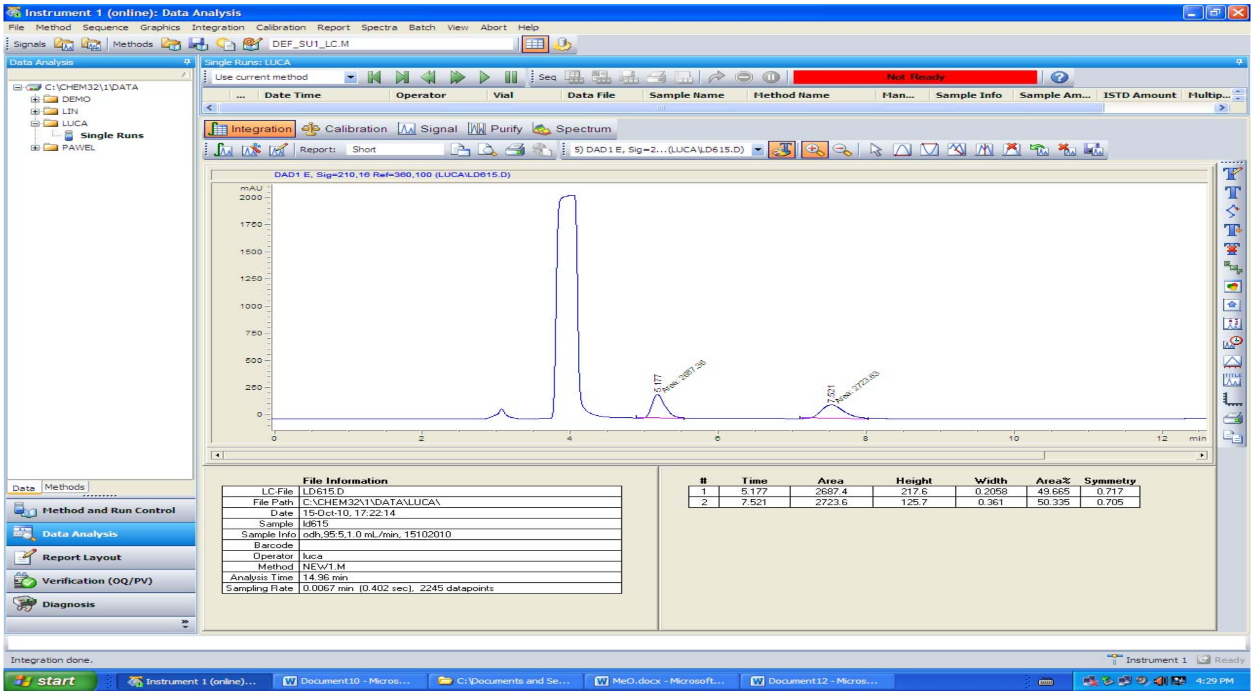
Instrument 1 (online): Data Analysis
DEF_SU1_LC.M
Single Runs: LUCA
Not Ready
Integration
Calibration
Signal
Purify
Spectrum
DAD1 E, Sig=210,16 Ref=360,100 (LUCA\LD615.D)
mAU
min
5.177
Area: 2687.38
7.521
Area: 2723.63
File Information
LC-File LD615.D
File Path C:\CHEM32\1\DATA\LUCA\
Date 15-Oct-10, 17:22:14
Sample ld615
Sample Info odh,95:5,1.0 mL/min, 15102010
Operator luca
Method NEW1.M
Analysis Time 14.96 min
Sampling Rate 0.0067 min (0.402 sec), 2245 datapoints
Time Area Height Width Area% Symmetry
1 5.177 2687.4 217.6 0.2058 49.665 0.717
2 7.521 2723.6 125.7 0.361 50.335 0.705
Integration done.

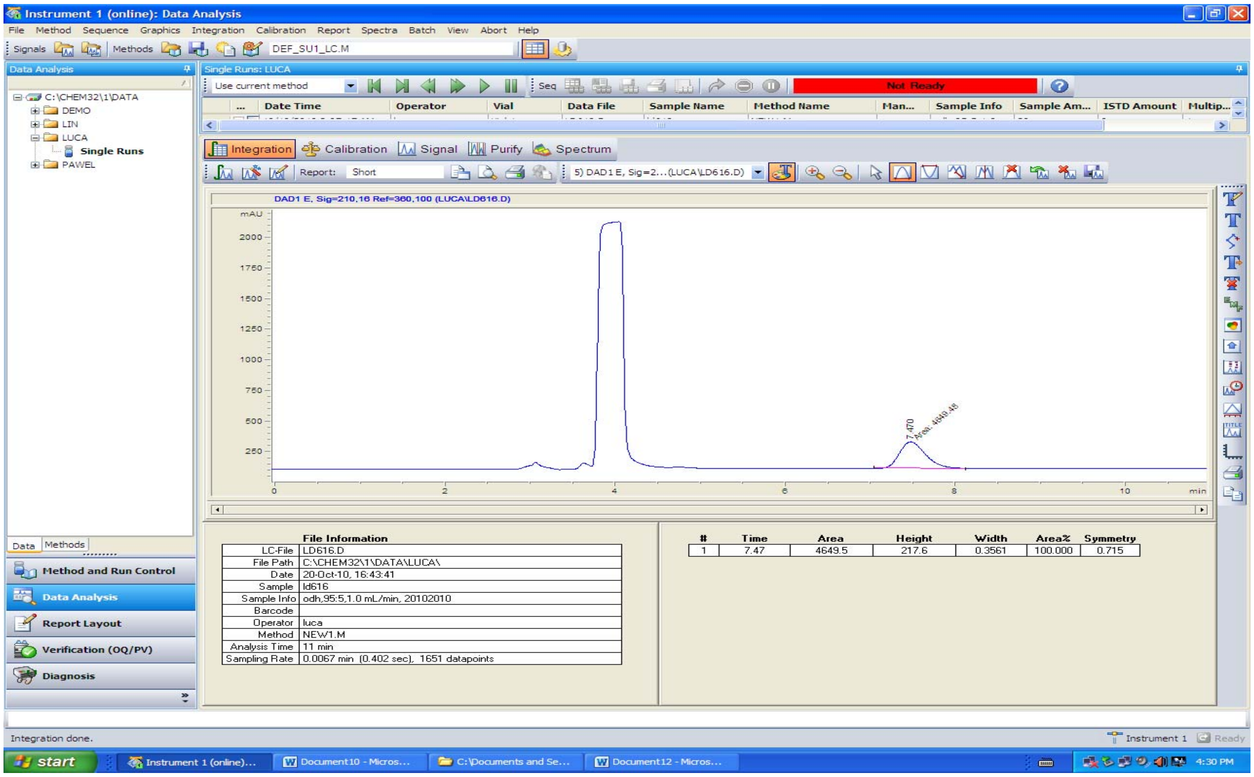
Instrument 1 (online): Data Analysis
DEF_SU1_LC.M
Single Runs: LUCA
Not Ready
Integration
Calibration
Signal
Purify
Spectrum
DAD1 E, Sig=210,16 Ref=360,100 (LUCA\LD616.D)
mAU
min
7.470
Area: 4649.46
File Information
LC-File LD616.D
File Path C:\CHEM32\1\DATA\LUCA\
Date 20-Oct-10, 16:43:41
Sample ld616
Sample Info odh,95:5,1.0 mL/min, 20102010
Operator luca
Method NEW1.M
Analysis Time 11 min
Sampling Rate 0.0067 min (0.402 sec), 1651 datapoints
Time Area Height Width Area% Symmetry
1 7.47 4649.5 217.6 0.3561 100.000 0.715
Integration done.

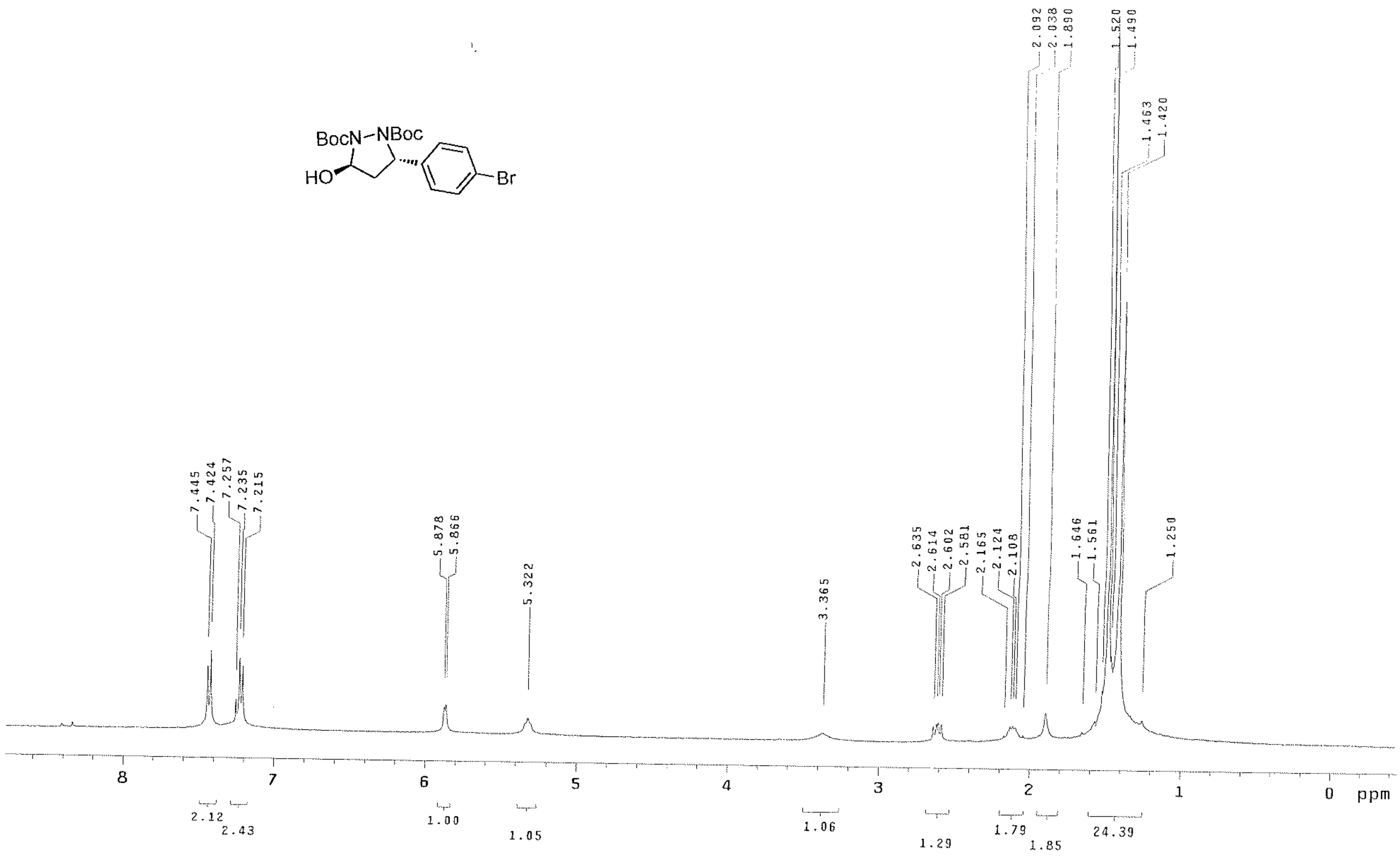

BocN–NBoc
HO
Br
7.445
7.424
7.257
7.235
7.215
5.878
5.866
5.322
3.365
2.635
2.614
2.602
2.581
2.165
2.124
2.108
2.092
2.038
1.890
1.646
1.561
1.520
1.490
1.463
1.420
1.250
8
7
6
5
4
3
2
1
0
ppm
2.12
2.43
1.00
1.05
1.06
1.29
1.79
1.85
24.39

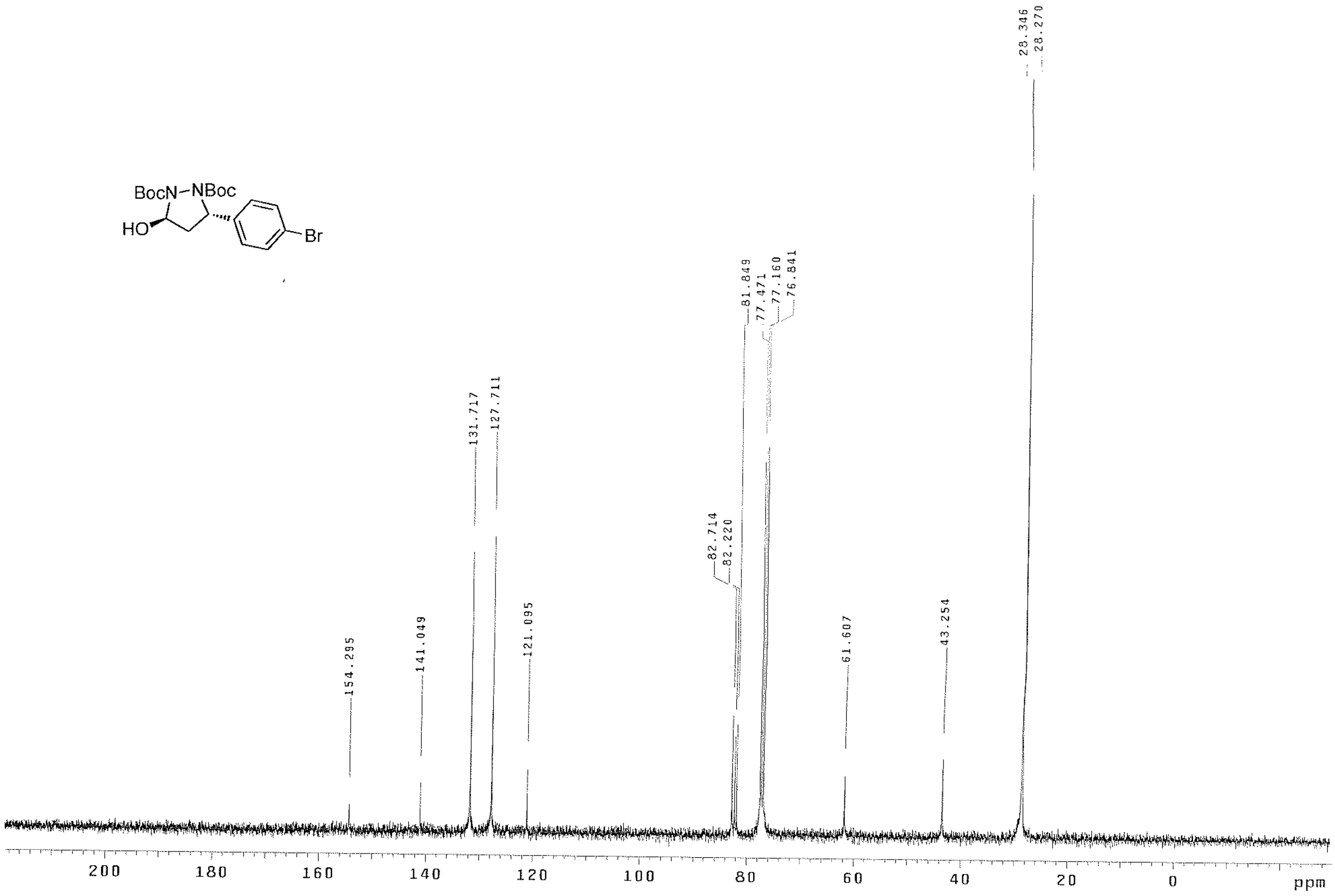

28.346
28.270
81.849
77.471
77.160
76.841
131.717
127.711
82.714
82.220
154.295
141.049
121.095
61.607
43.254
200
180
160
140
120
100
80
60
40
20
0
ppm

Display Report

Analysis Info

Analysis Name	H:\Data2\Luca\ld585000001.d	Acquisition Date	2011-03-01 14:38:17
Method	tune_low_dirk.m	Operator	pia
Sample Name	ld585	Instrument / Ser#	micrOTOF 125
Comment			

Acquisition Parameter

Source Type	ESI	Ion Polarity	Positive	Set Nebulizer	0.4 Bar
Focus	Not active			Set Dry Heater	170 °C
Scan Begin	50 m/z	Set Capillary	4500 V	Set Dry Gas	4.0 l/min
Scan End	3000 m/z	Set End Plate Offset	-500 V	Set Divert Valve	Source

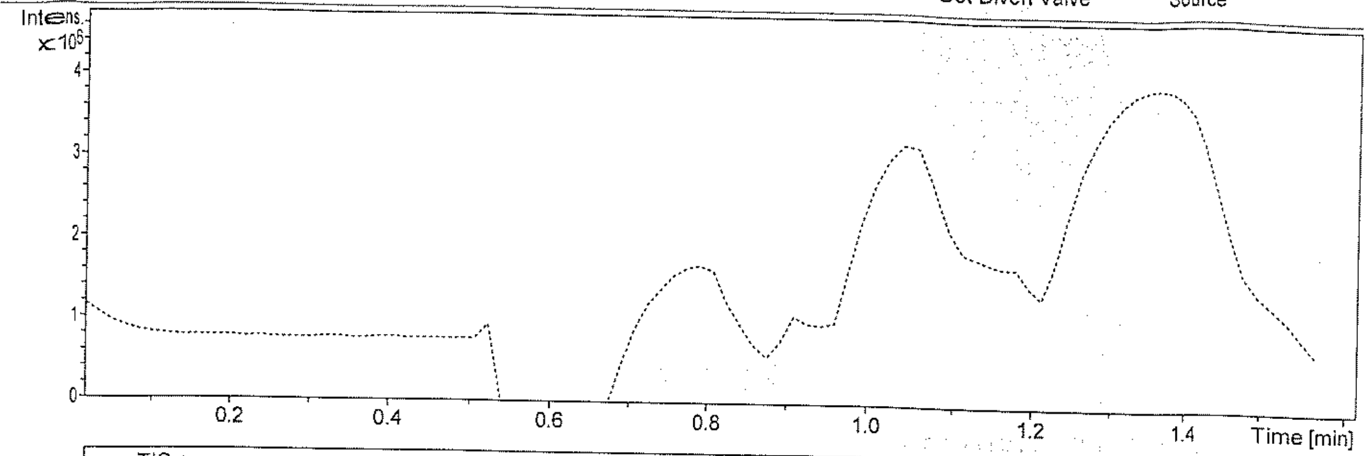

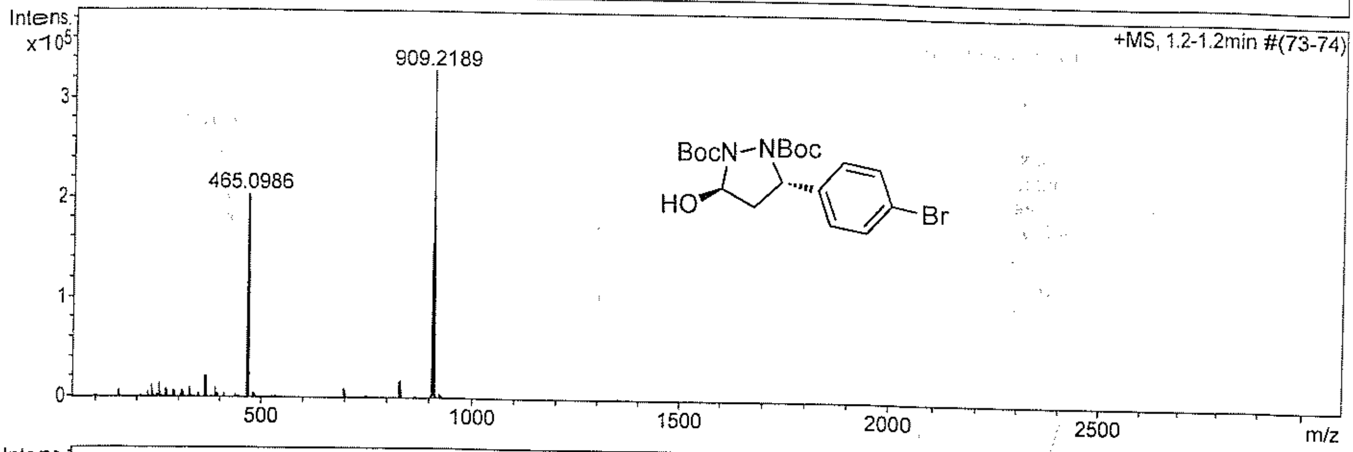

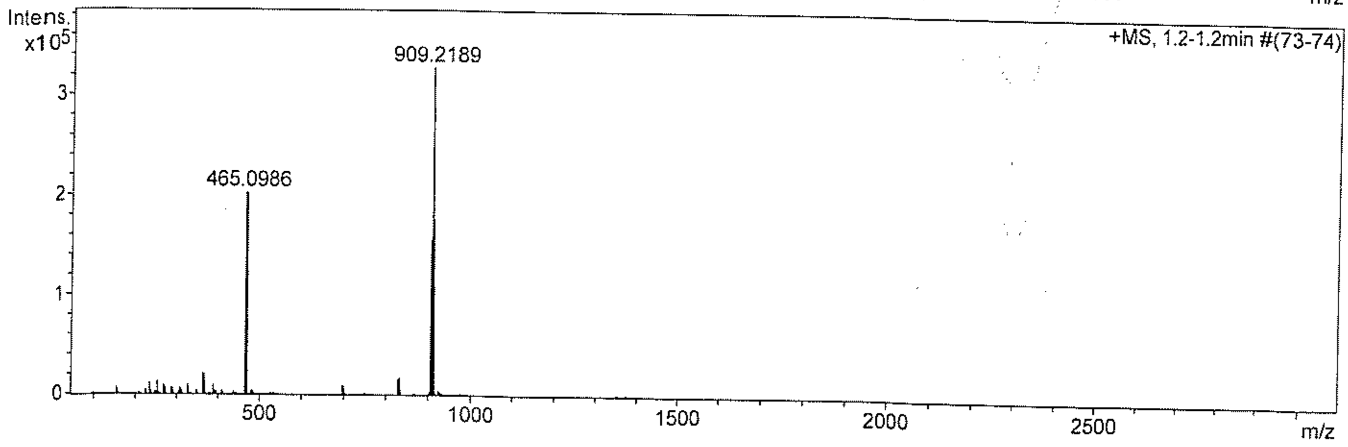

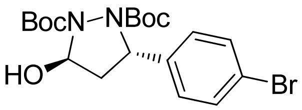
BocN
NBoc
HO
Br

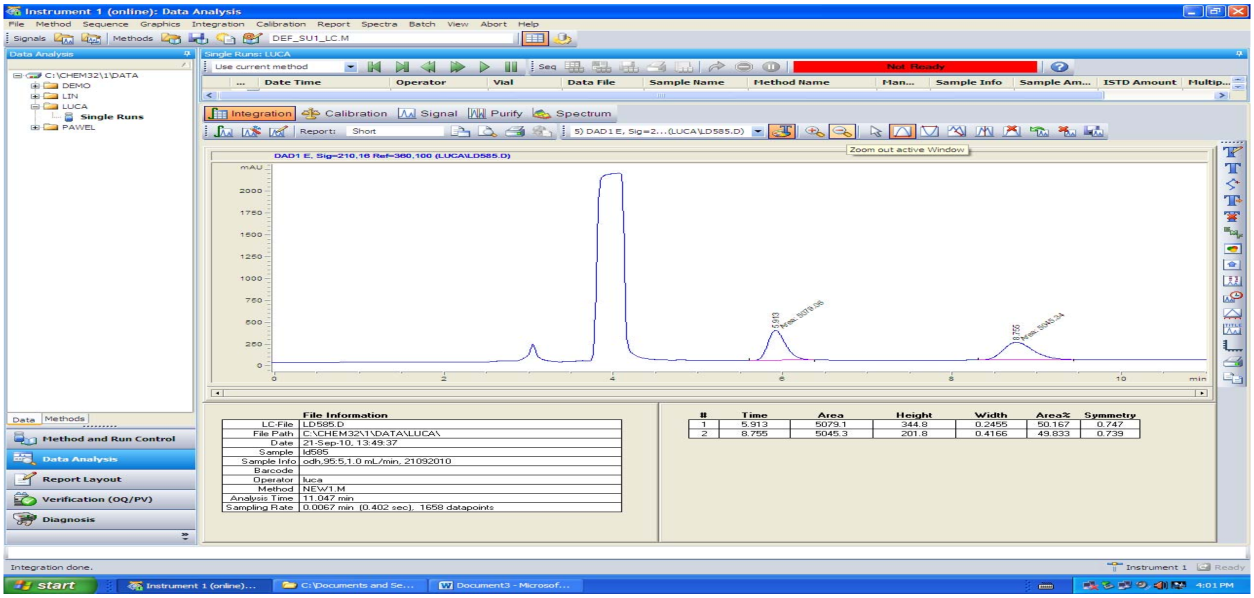
Instrument 1 (online): Data Analysis
DAD1 E, Sig=210,16 Ref=360,100 (LUCA\LD585.D)
File Information
LC-File LD585.D
File Path C:\CHEM32\1\DATA\LUCA\
Date 21-Sep-10, 13:49:37
Sample ld585
Sample Info odh,95:5,1.0 mL/min, 21092010
Operator luca
Method NEW1.M
Analysis Time 11.047 min
Sampling Rate 0.0067 min (0.402 sec), 1658 datapoints
Time Area Height Width Area% Symmetry
1 5.913 5079.1 344.8 0.2455 50.167 0.747
2 8.755 5045.3 201.8 0.4166 49.833 0.739

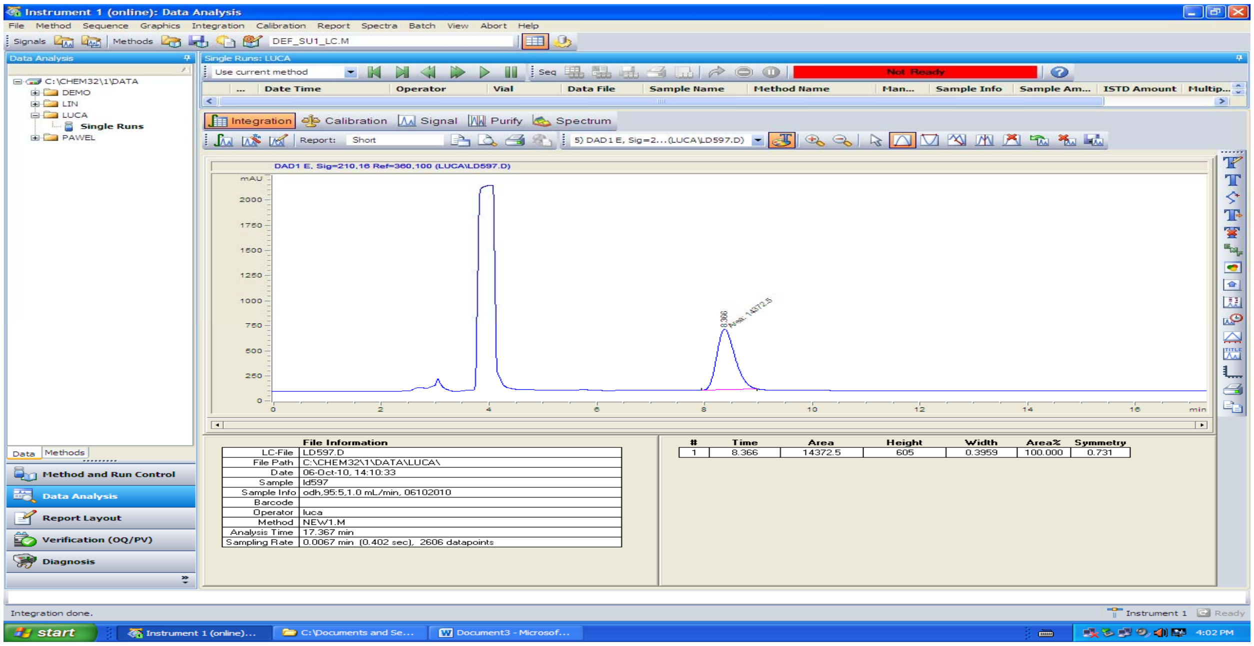
Instrument 1 (online): Data Analysis
DAD1 E, Sig=210,16 Ref=360,100 (LUCA\LD597.D)
File Information
LC-File LD597.D
File Path C:\CHEM32\1\DATA\LUCA\
Date 06-Oct-10, 14:10:33
Sample ld597
Sample Info odh,95:5,1.0 mL/min, 06102010
Operator luca
Method NEW1.M
Analysis Time 17.367 min
Sampling Rate 0.0067 min (0.402 sec), 2606 datapoints
Time Area Height Width Area% Symmetry
1 8.366 14372.5 605 0.3959 100.000 0.731

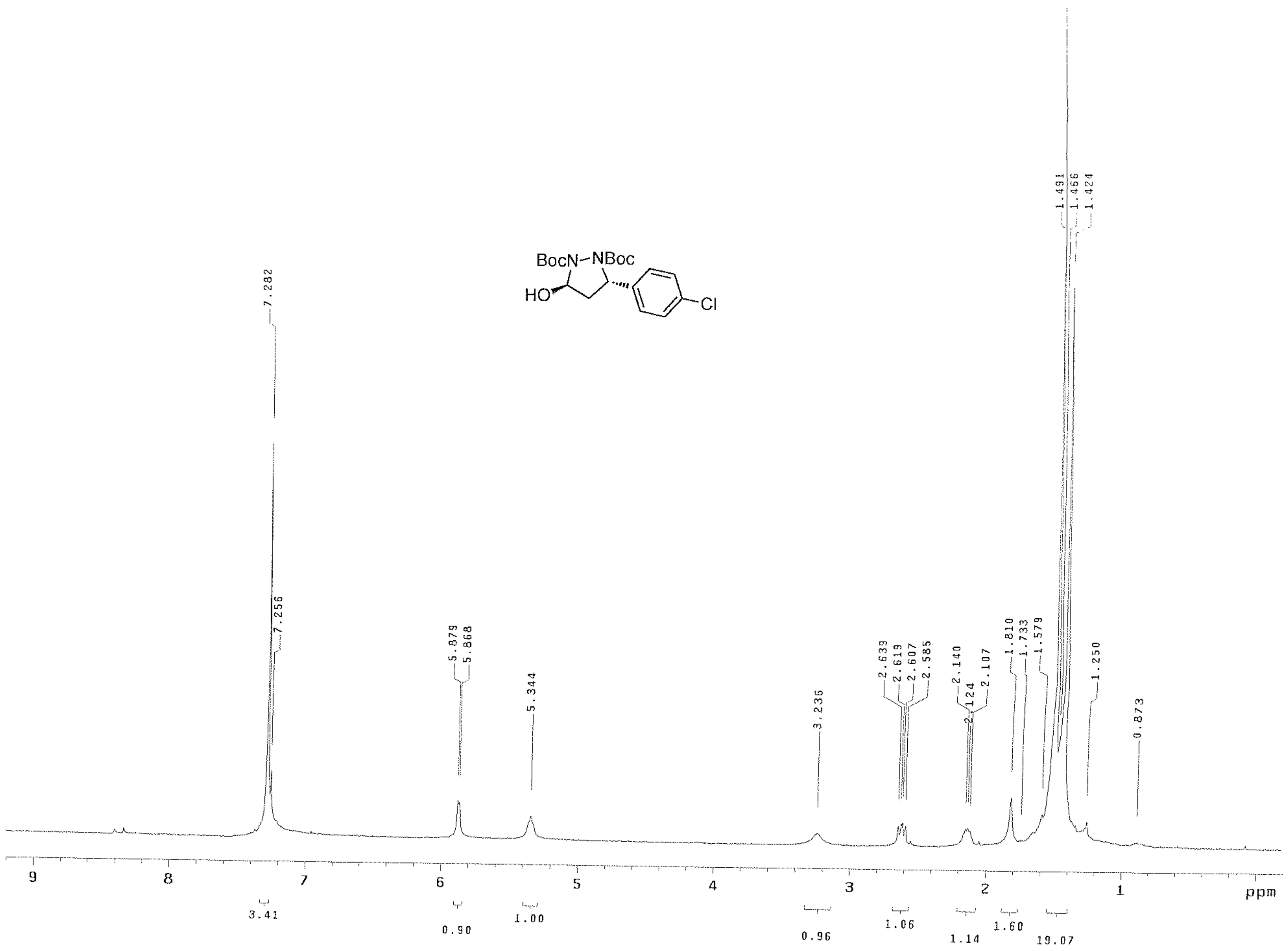
BocN–NBoc
HO
Cl
7.282
7.256
5.879
5.868
5.344
3.236
2.639
2.619
2.607
2.585
2.140
2.124
2.107
1.810
1.733
1.579
1.491
1.466
1.424
1.250
0.873
9
8
7
6
5
4
3
2
1
ppm
3.41
0.90
1.00
0.96
1.06
1.14
1.60
19.07

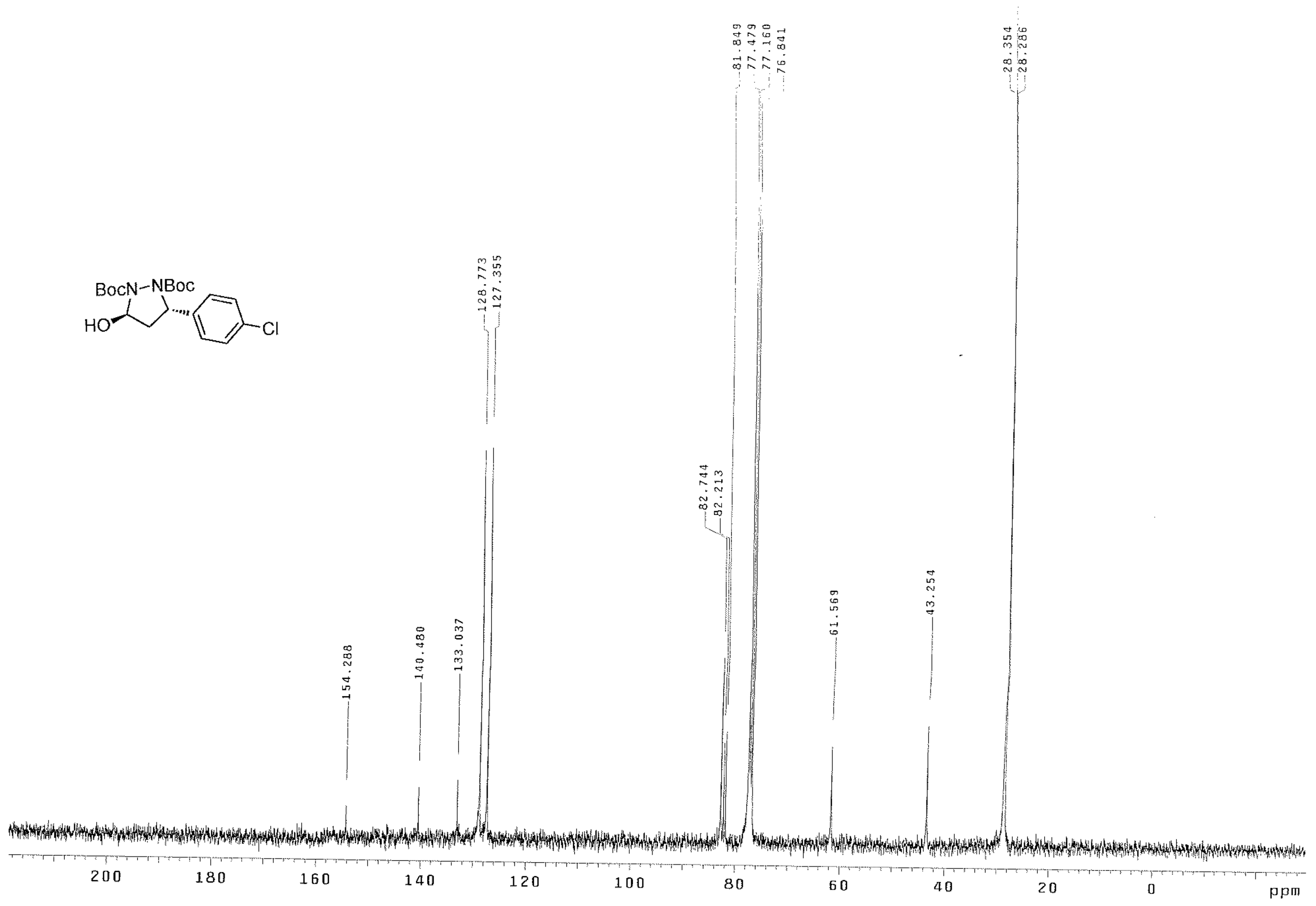
BocN–NBoc
HO
Cl
154.288
140.480
133.037
128.773
127.355
82.744
82.213
81.849
77.479
77.160
76.841
61.569
43.254
28.354
28.286
200
180
160
140
120
100
80
60
40
20
0
ppm

Display Report

Analysis Info

Analysis Name	H:\Data2\Luca\ld584000001.d	Acquisition Date	2011-03-01 13:53:33
Method	tune_low_dirk.m	Operator	pia
Sample Name	ld584	Instrument / Ser#	micrOTOF 125
Comment			

Acquisition Parameter

Source Type	ESI	Ion Polarity	Positive	Set Nebulizer	0.4 Bar
Focus	Not active			Set Dry Heater	170 °C
Scan Begin	50 m/z	Set Capillary	4500 V	Set Dry Gas	4.0 l/min
Scan End	3000 m/z	Set End Plate Offset	-500 V	Set Divert Valve	Source

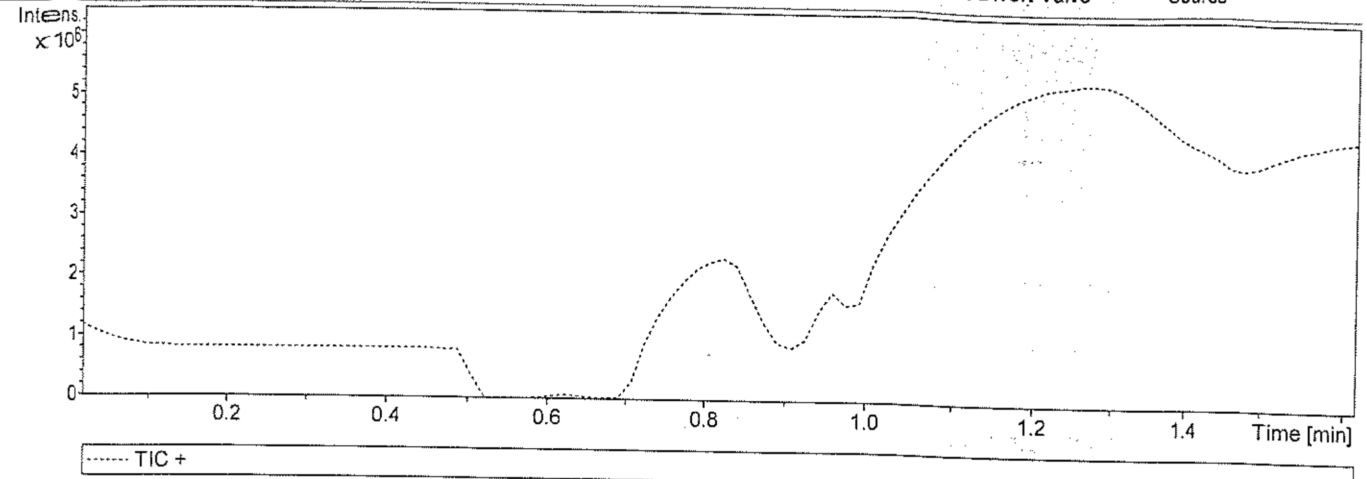

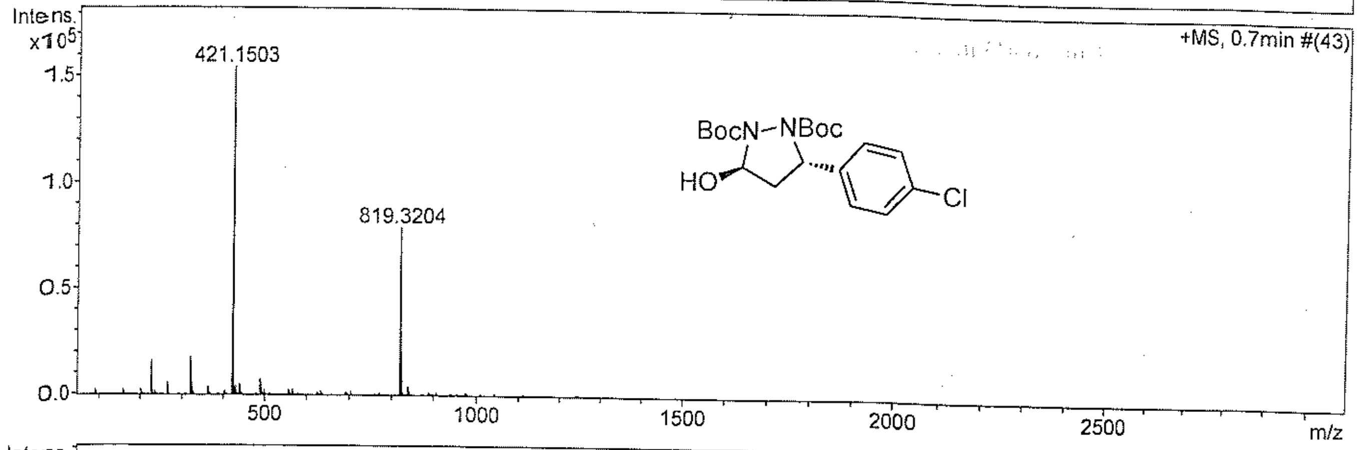

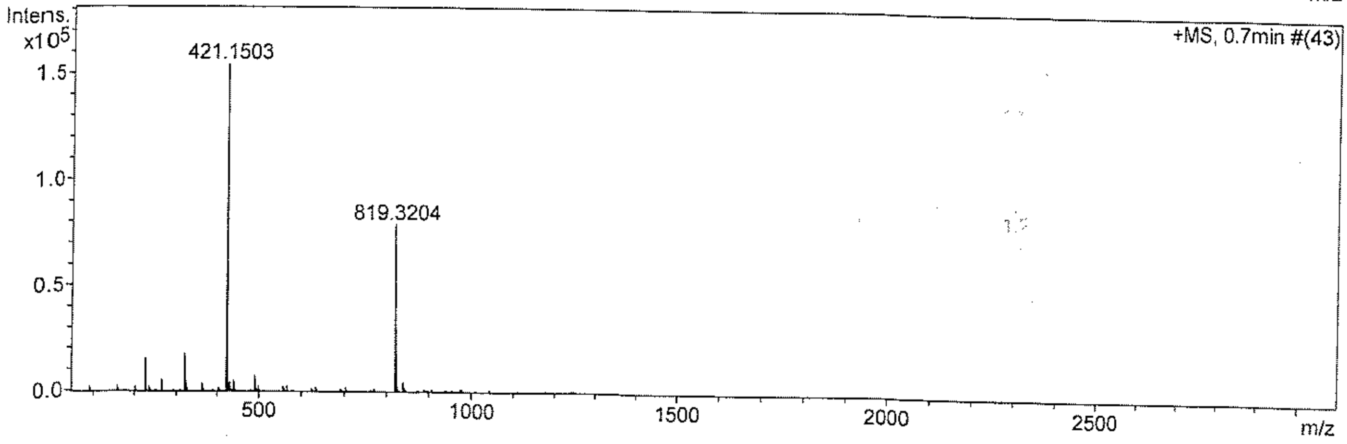

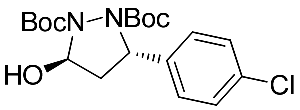
BocN
NBoc
HO
Cl

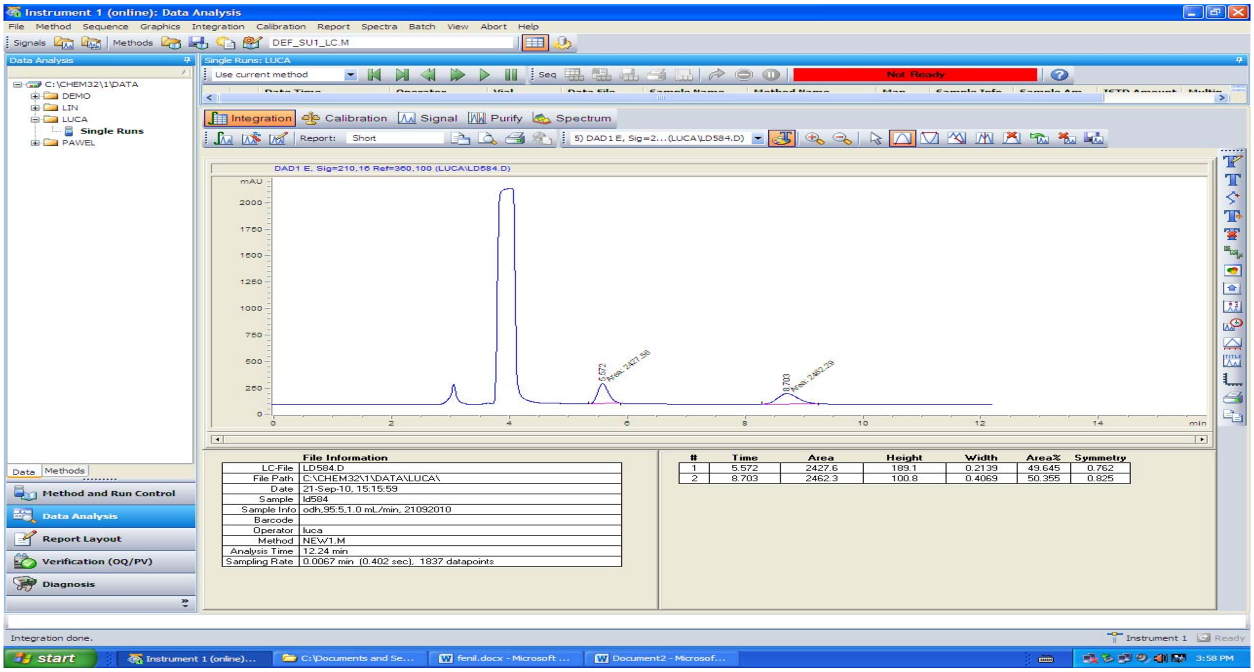
Instrument 1 (online): Data Analysis
DAD1 E, Sig=210,16 Ref=360,100 (LUCA\LD584.D)
File Information
LC-File LD584.D
File Path C:\CHEM32\1\DATA\LUCA\
Date 21-Sep-10, 15:15:59
Sample ld584
Sample Info odh,95:5,1.0 mL/min, 21092010
Operator luca
Method NEW1.M
Analysis Time 12.24 min
Sampling Rate 0.0067 min (0.402 sec), 1837 datapoints
Time Area Height Width Area% Symmetry
1 5.572 2427.6 189.1 0.2139 49.645 0.762
2 8.703 2462.3 100.8 0.4069 50.355 0.825

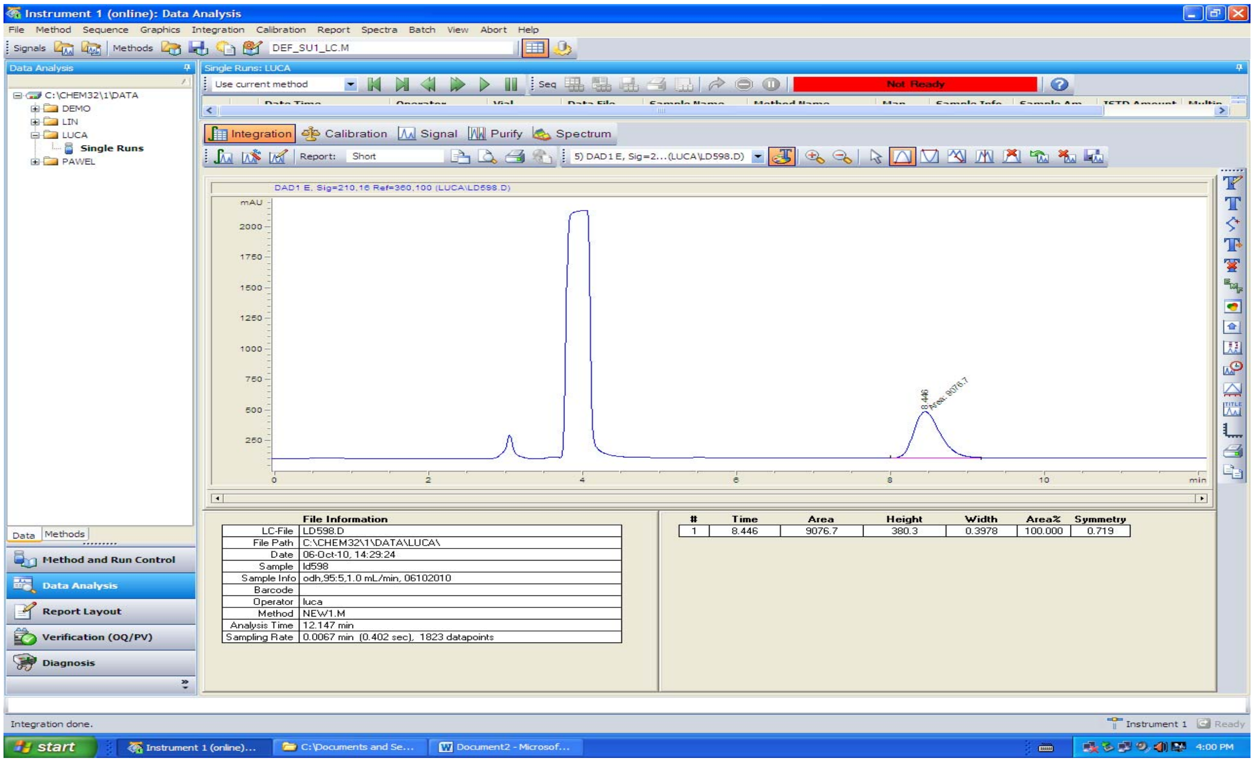
Instrument 1 (online): Data Analysis
DAD1 E, Sig=210,16 Ref=360,100 (LUCA\LD598.D)
File Information
LC-File LD598.D
File Path C:\CHEM32\1\DATA\LUCA\
Date 06-Oct-10, 14:29:24
Sample ld598
Sample Info odh,95:5,1.0 mL/min, 06102010
Operator luca
Method NEW1.M
Analysis Time 12.147 min
Sampling Rate 0.0067 min (0.402 sec), 1823 datapoints
Time Area Height Width Area% Symmetry
1 8.446 9076.7 380.3 0.3978 100.000 0.719

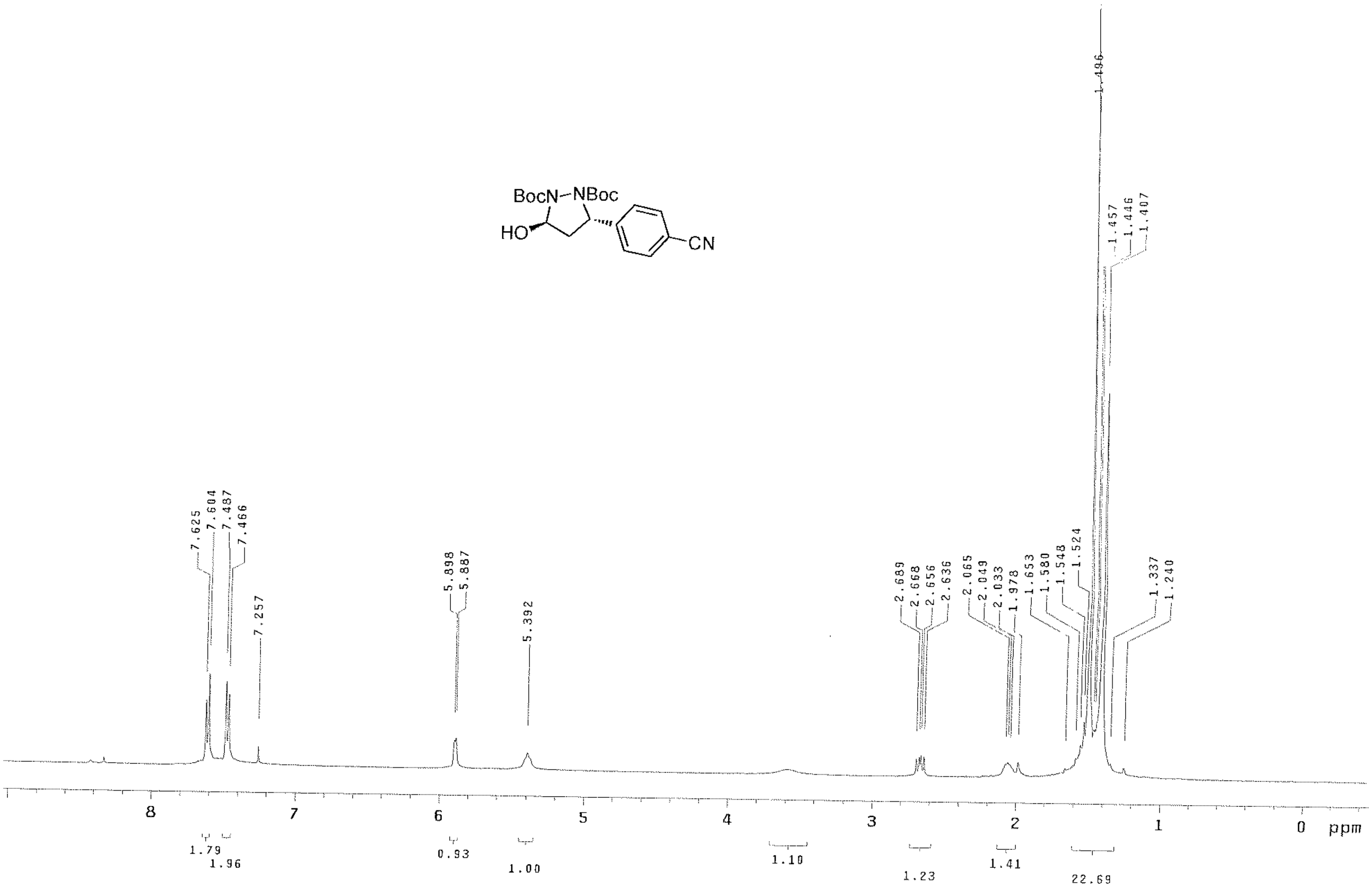
BocN-NBoc
HO
CN
7.625
7.604
7.487
7.466
7.257
5.898
5.887
5.392
2.689
2.668
2.656
2.636
2.065
2.049
2.033
1.978
1.653
1.580
1.548
1.524
1.496
1.457
1.446
1.407
1.337
1.240
8
7
6
5
4
3
2
1
0
ppm
1.79
1.96
0.93
1.00
1.10
1.23
1.41
22.69

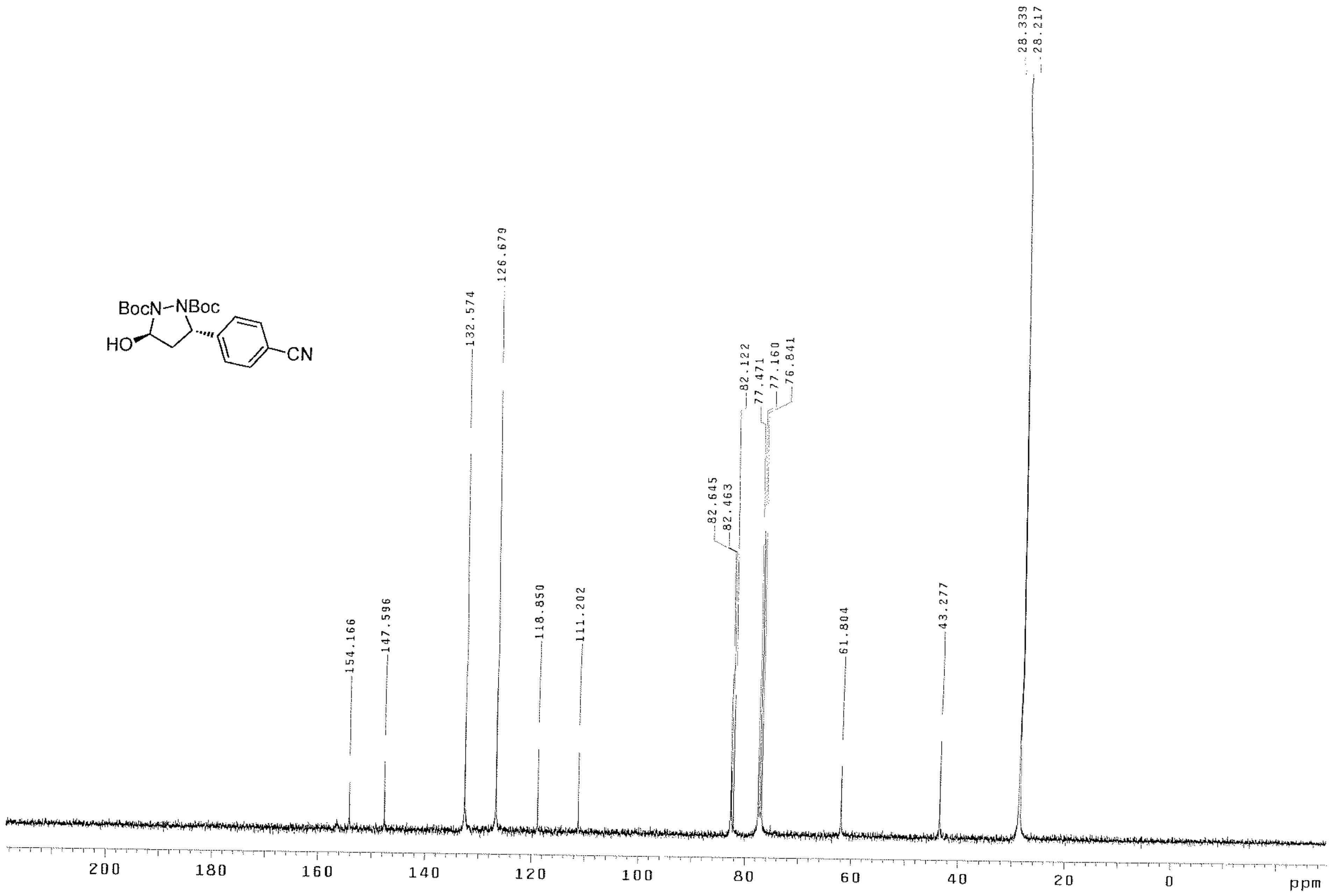

BocN-NBoc
HO
CN
154.166
147.596
132.574
126.679
118.850
111.202
82.645
82.463
82.122
77.471
77.160
76.841
61.804
43.277
28.339
28.217
200
180
160
140
120
100
80
60
40
20
0
ppm

Display Report

Analysis Info

Analysis Name	H:\Data2\Luca\ld588000001.d	Acquisition Date	2011-03-01 14:54:04
Method	tune_low_dirk.m	Operator	pia
Sample Name	ld588	Instrument / Ser#	micrOTOF 125
Comment			

Acquisition Parameter

Source Type	ESI	Ion Polarity	Positive	Set Nebulizer	0.4 Bar
Focus	Not active			Set Dry Heater	170 °C
Scan Begin	50 m/z	Set Capillary	4500 V	Set Dry Gas	4.0 l/min
Scan End	3000 m/z	Set End Plate Offset	-500 V	Set Divert Valve	Source

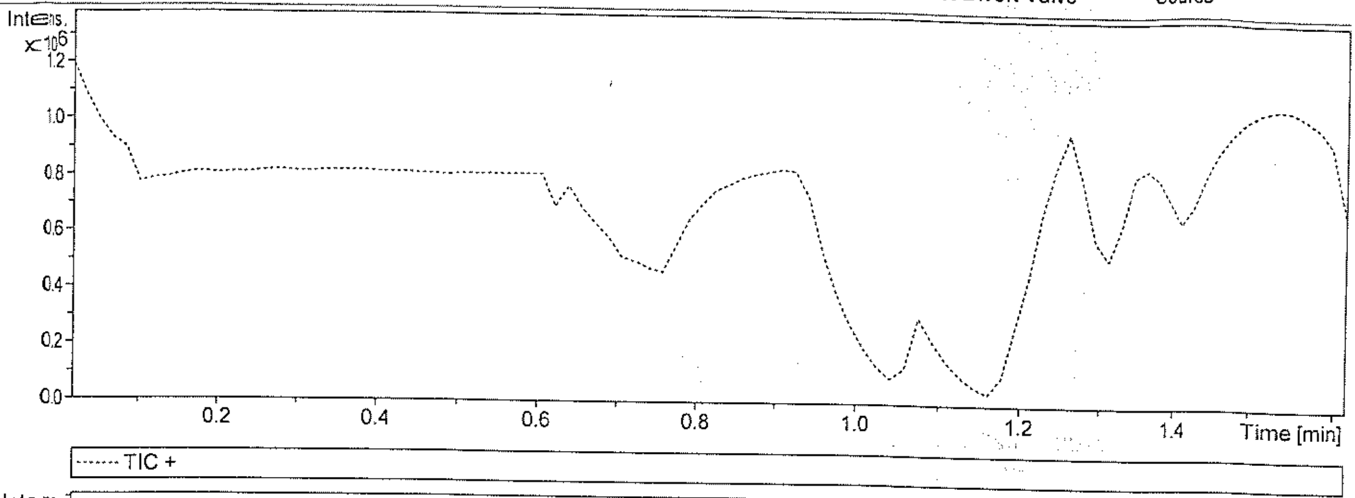

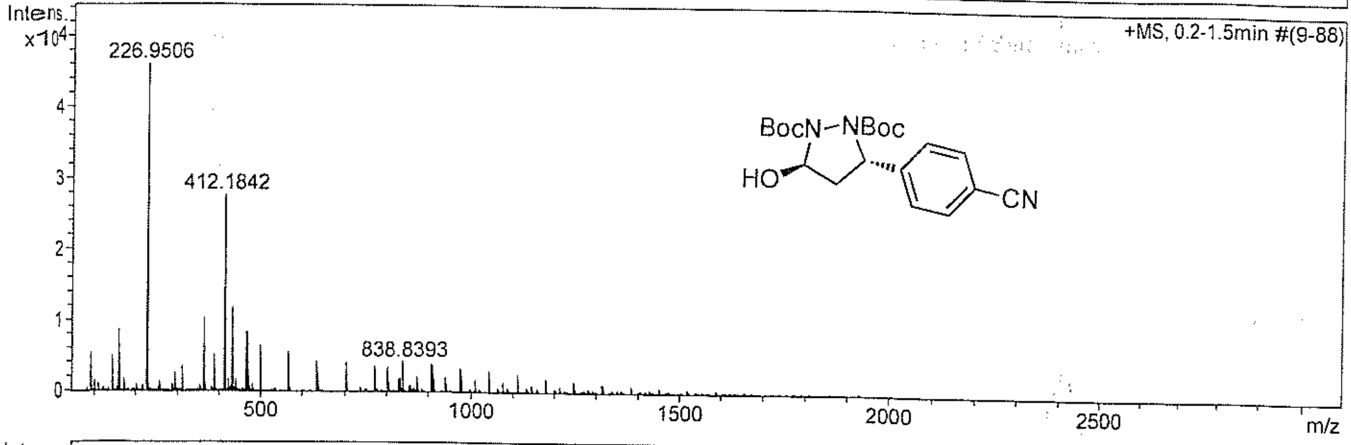

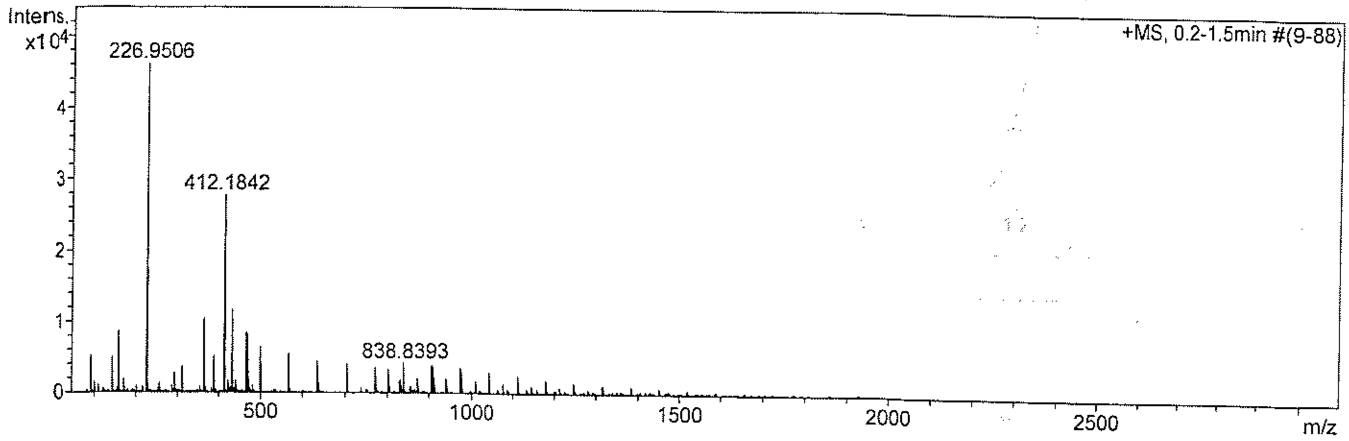

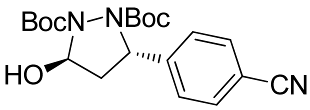
BocN
NBoc
HO
CN

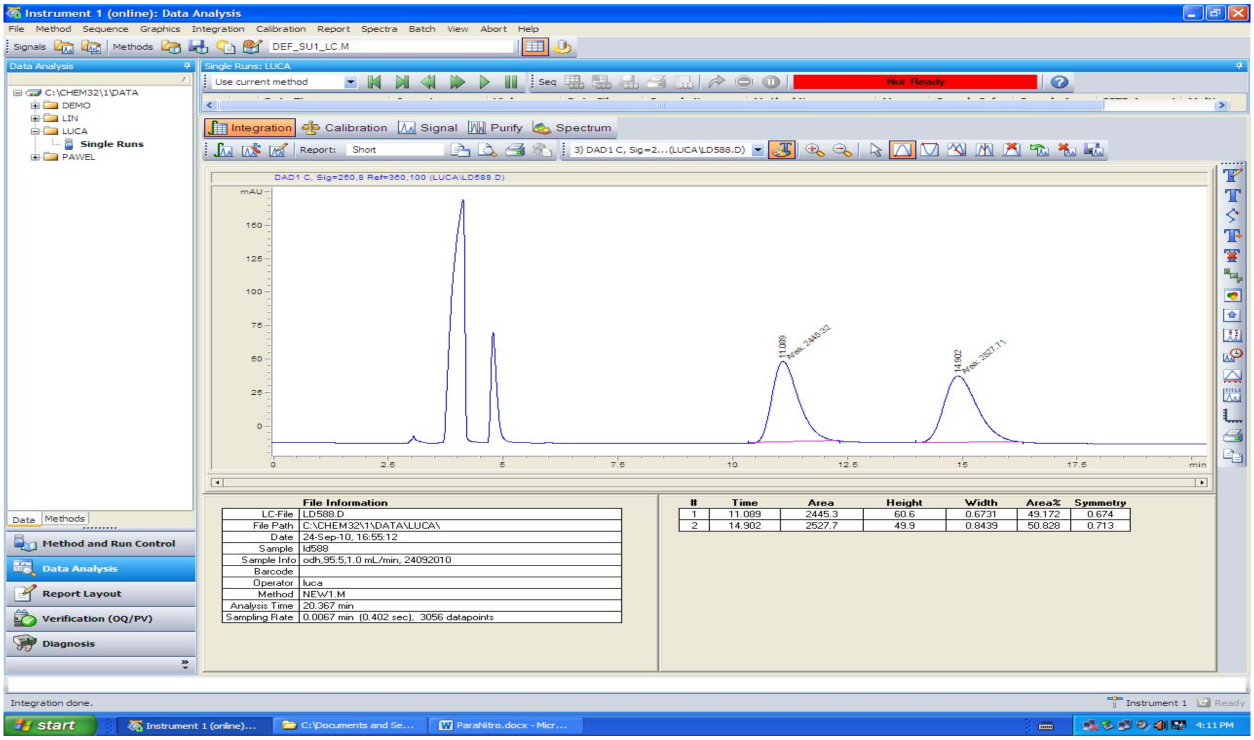
Instrument 1 (online): Data Analysis
DAD1 C, Sig=250,8 Ref=360,100 (LUCA\LD588.D)
11.089 Area: 2445.32
14.902 Area: 2527.71
File Information
LC-File LD588.D
File Path C:\CHEM32\1\DATA\LUCA\
Date 24-Sep-10, 16:55:12
Sample ld588
Sample Info odh,95:5,1.0 mL/min, 24092010
Operator luca
Method NEW1.M
Analysis Time 20.367 min
Sampling Rate 0.0067 min (0.402 sec), 3056 datapoints
Time Area Height Width Area% Symmetry
1 11.089 2445.3 60.6 0.6731 49.172 0.674
2 14.902 2527.7 49.9 0.8439 50.828 0.713
Integration done.

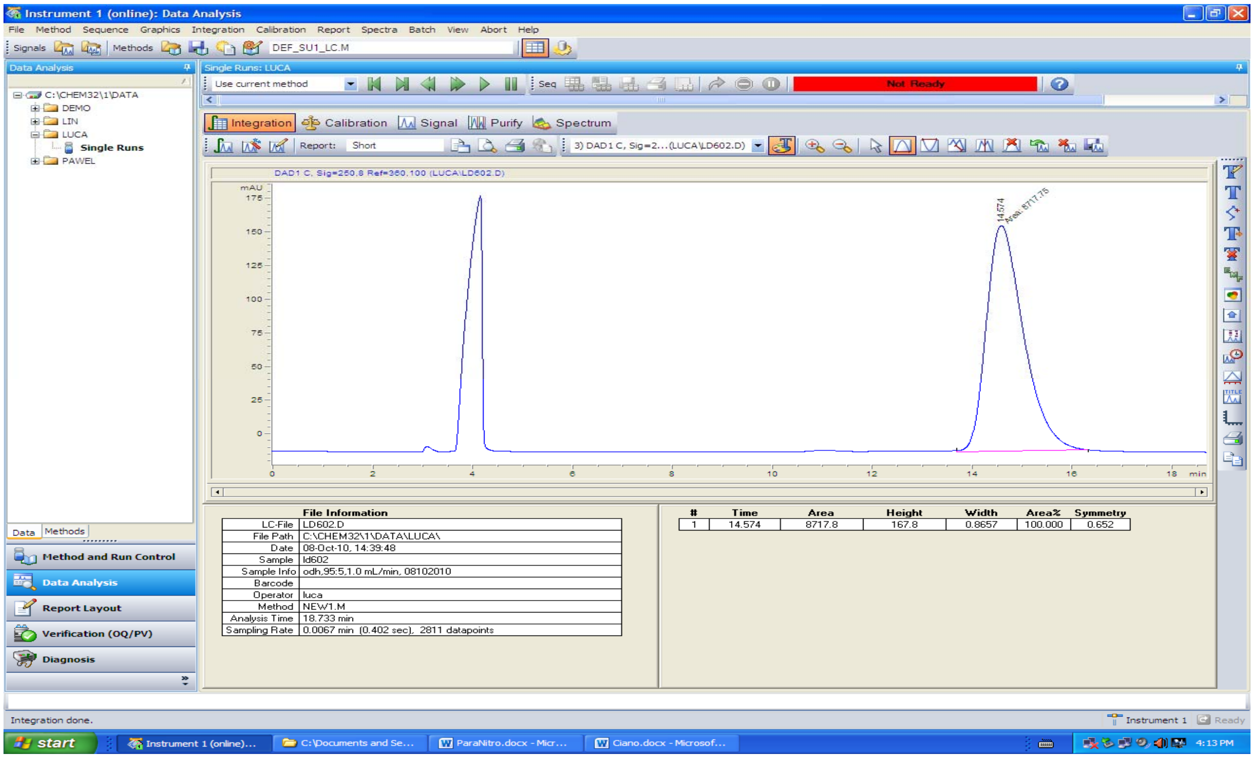
Instrument 1 (online): Data Analysis
DAD1 C, Sig=250,8 Ref=360,100 (LUCA\LD602.D)
14.574 Area: 8717.75
File Information
LC-File LD602.D
File Path C:\CHEM32\1\DATA\LUCA\
Date 08-Oct-10, 14:39:48
Sample ld602
Sample Info odh,95:5,1.0 mL/min, 08102010
Operator luca
Method NEW1.M
Analysis Time 18.733 min
Sampling Rate 0.0067 min (0.402 sec), 2811 datapoints
Time Area Height Width Area% Symmetry
1 14.574 8717.8 167.8 0.8657 100.000 0.652
Integration done.

587H

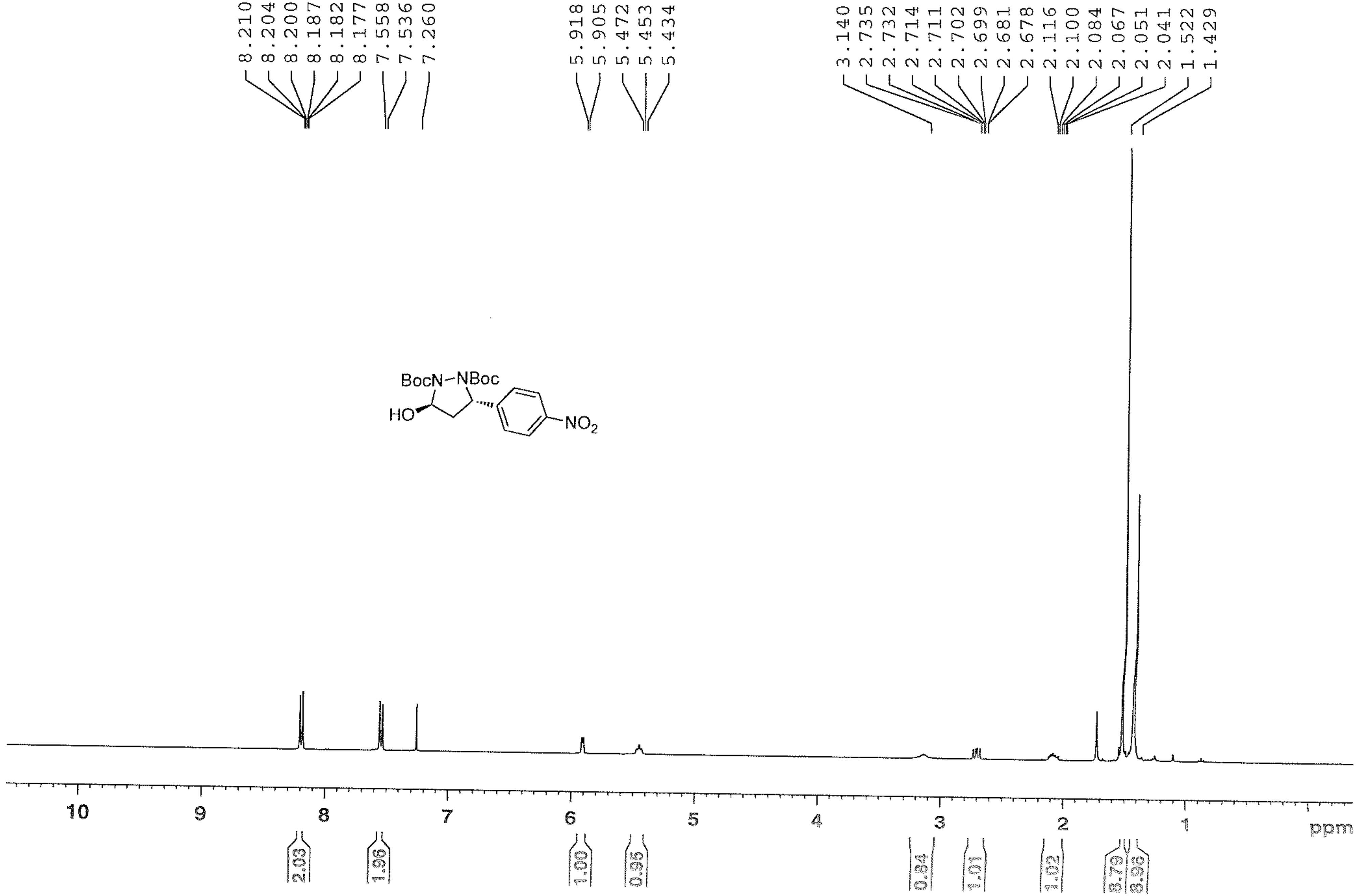
8.210
8.204
8.200
8.187
8.182
8.177
7.558
7.536
7.260
5.918
5.905
5.472
5.453
5.434
3.140
2.735
2.732
2.714
2.711
2.702
2.699
2.681
2.678
2.116
2.100
2.084
2.067
2.051
2.041
1.522
1.429
BocN
NBoc
HO
NO2
10
9
8
7
6
5
4
3
2
1
ppm
2.03
1.96
1.00
0.95
0.84
1.01
1.02
8.79
8.96

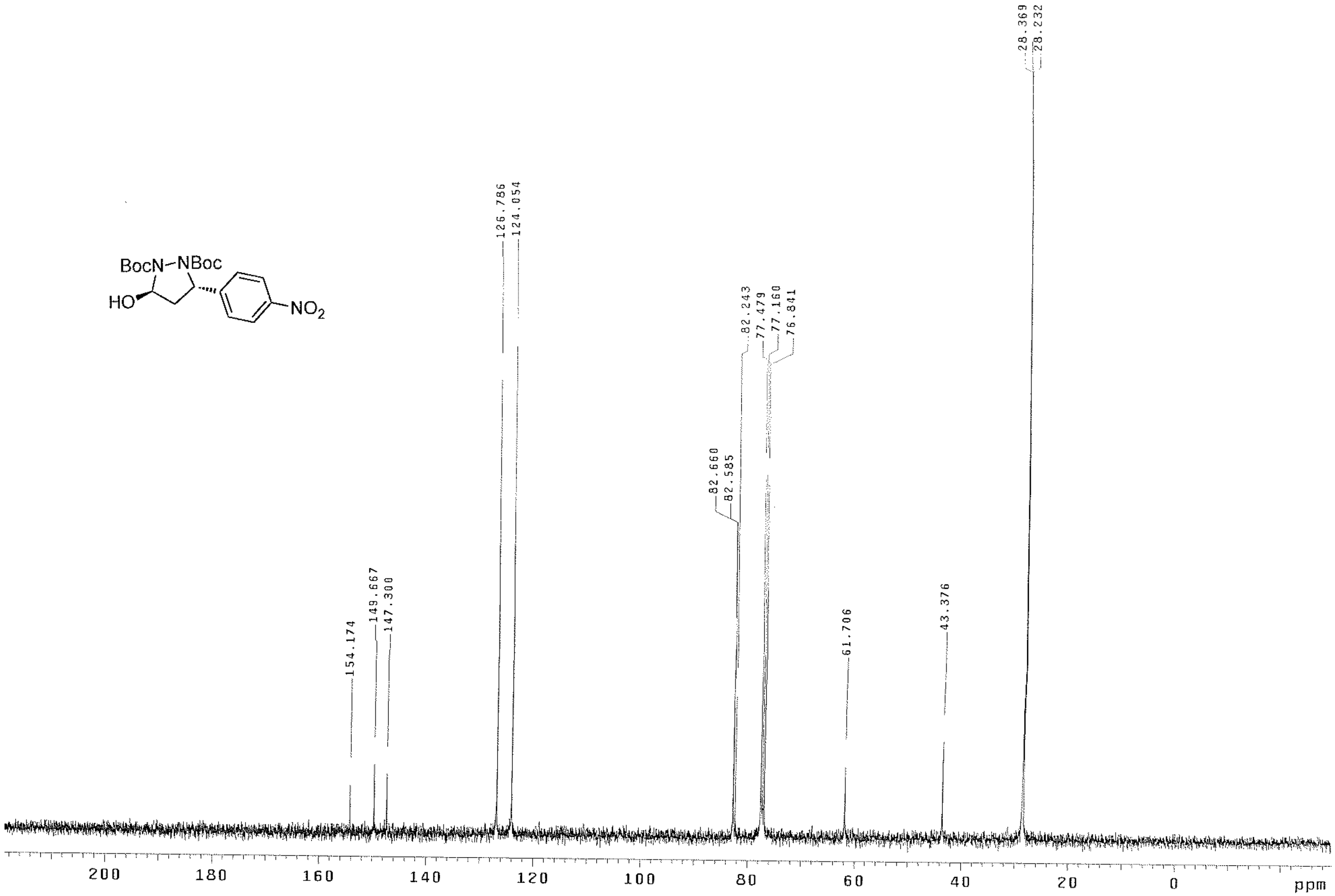
BocN–NBoc
HO
NO2
154.174
149.667
147.300
126.786
124.054
82.660
82.585
82.243
77.479
77.160
76.841
61.706
43.376
28.369
28.232
200
180
160
140
120
100
80
60
40
20
0
ppm

Display Report

Analysis Info

Analysis Name	H:\Data2\Luca\ld587000001.d
Method	tune_low_dirk.m
Sample Name	ld587
Comment	

Acquisition Date	2011-03-01 13:26:55
Operator	pia
Instrument / Ser#	micrOTOF 125

Acquisition Parameter

Source Type	ESI	Ion Polarity	Positive	Set Nebulizer	0.4 Bar
Focus	Not active			Set Dry Heater	170 °C
Scan Begin	50 m/z	Set Capillary	4500 V	Set Dry Gas	4.0 l/min
Scan End	3000 m/z	Set End Plate Offset	-500 V	Set Divert Valve	Source

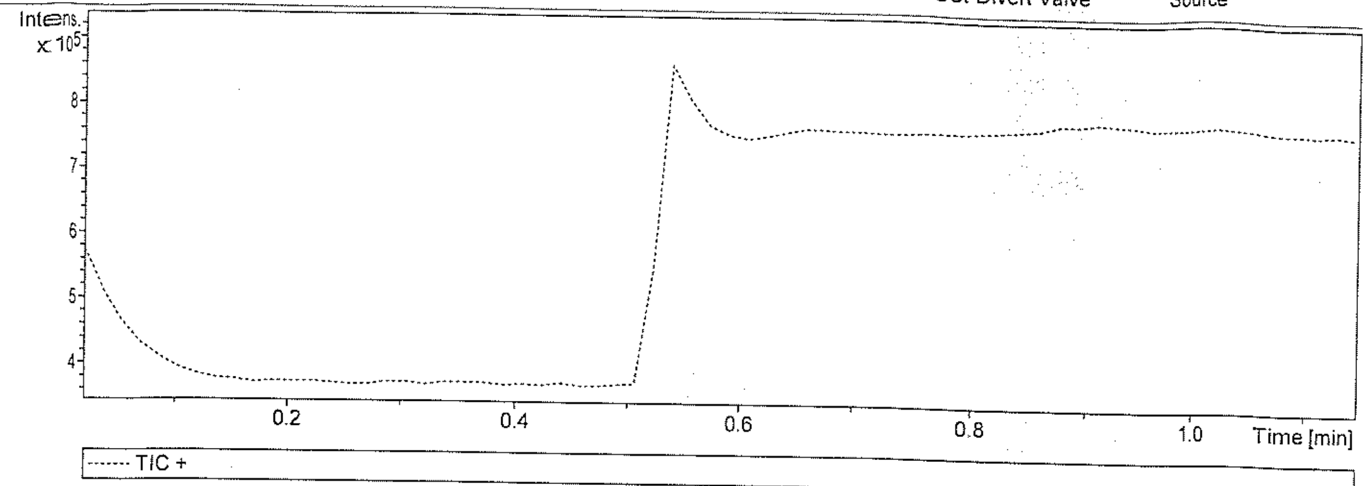

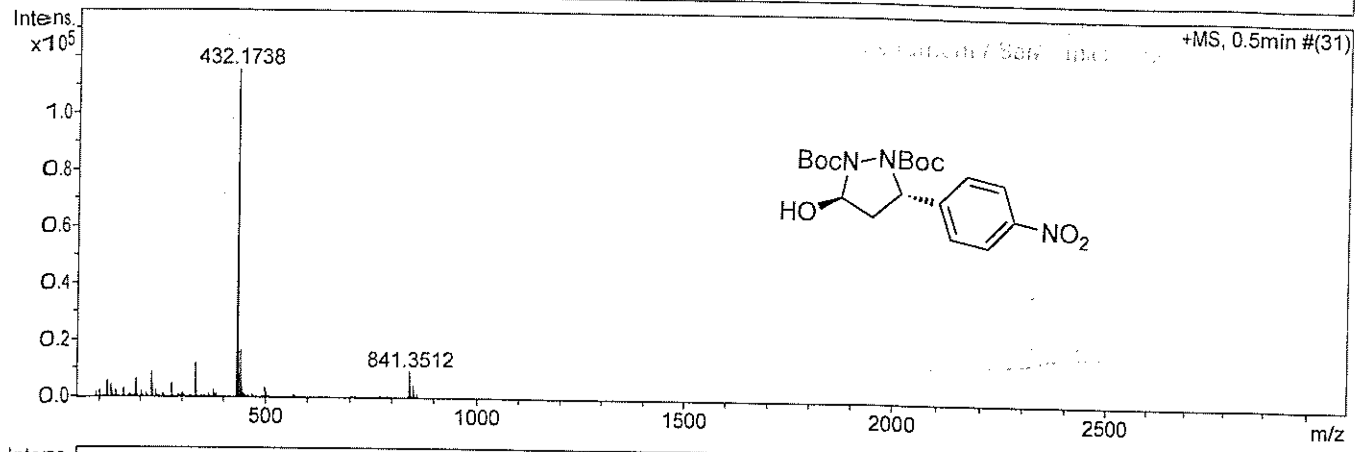

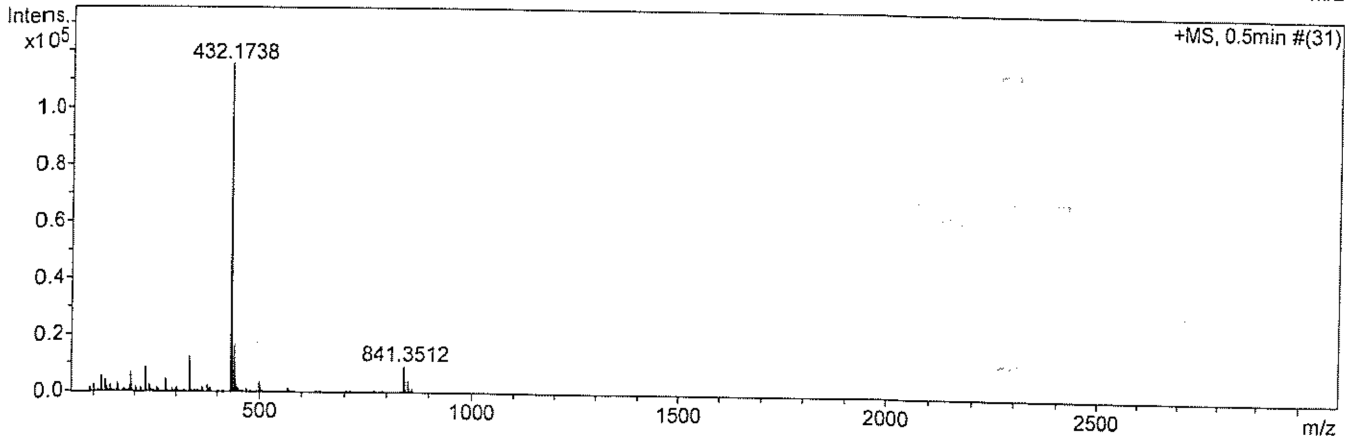

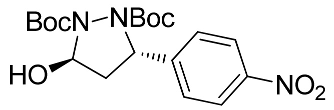
BocN–NBoc
HO
NO2

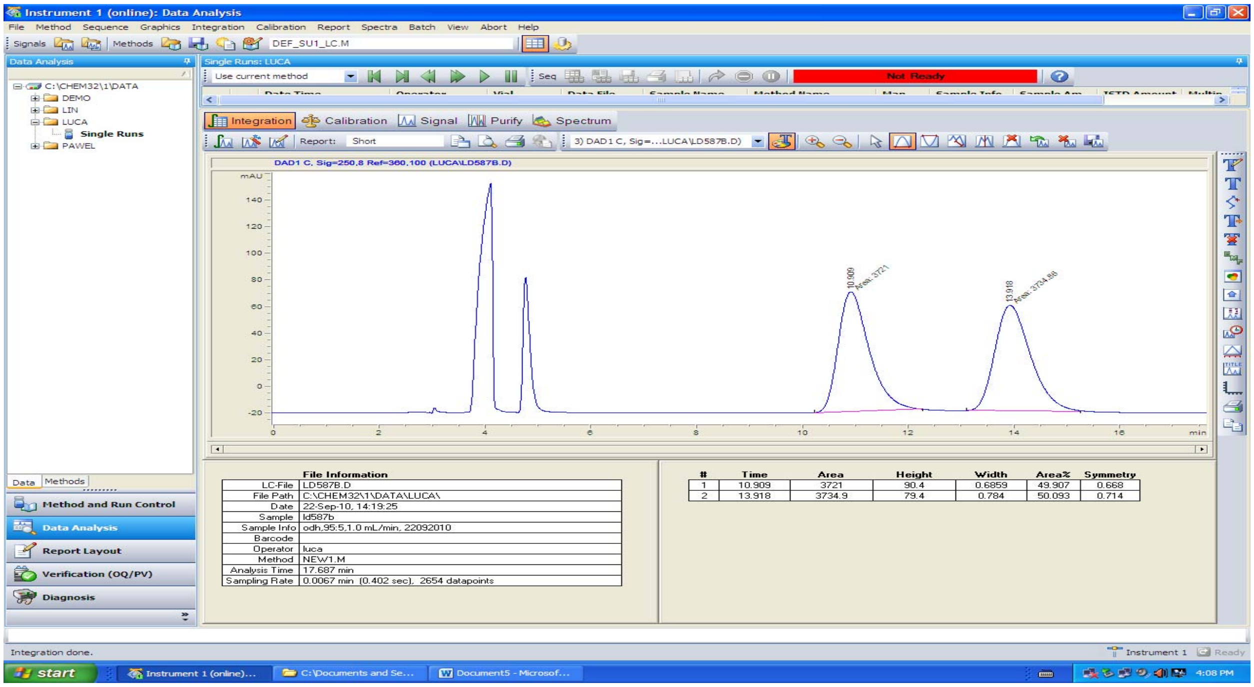
Instrument 1 (online): Data Analysis
DAD1 C, Sig=250,8 Ref=360,100 (LUCA\LD587B.D)
10.909 Area: 3721
13.918 Area: 3734.86
File Information
LC-File LD587B.D
File Path C:\CHEM32\1\DATA\LUCA\
Date 22-Sep-10, 14:19:25
Sample ld587b
Sample Info odh,95:5,1.0 mL/min, 22092010
Operator luca
Method NEW1.M
Analysis Time 17.687 min
Sampling Rate 0.0067 min (0.402 sec), 2654 datapoints
Time Area Height Width Area% Symmetry
1 10.909 3721 90.4 0.6859 49.907 0.668
2 13.918 3734.9 79.4 0.784 50.093 0.714

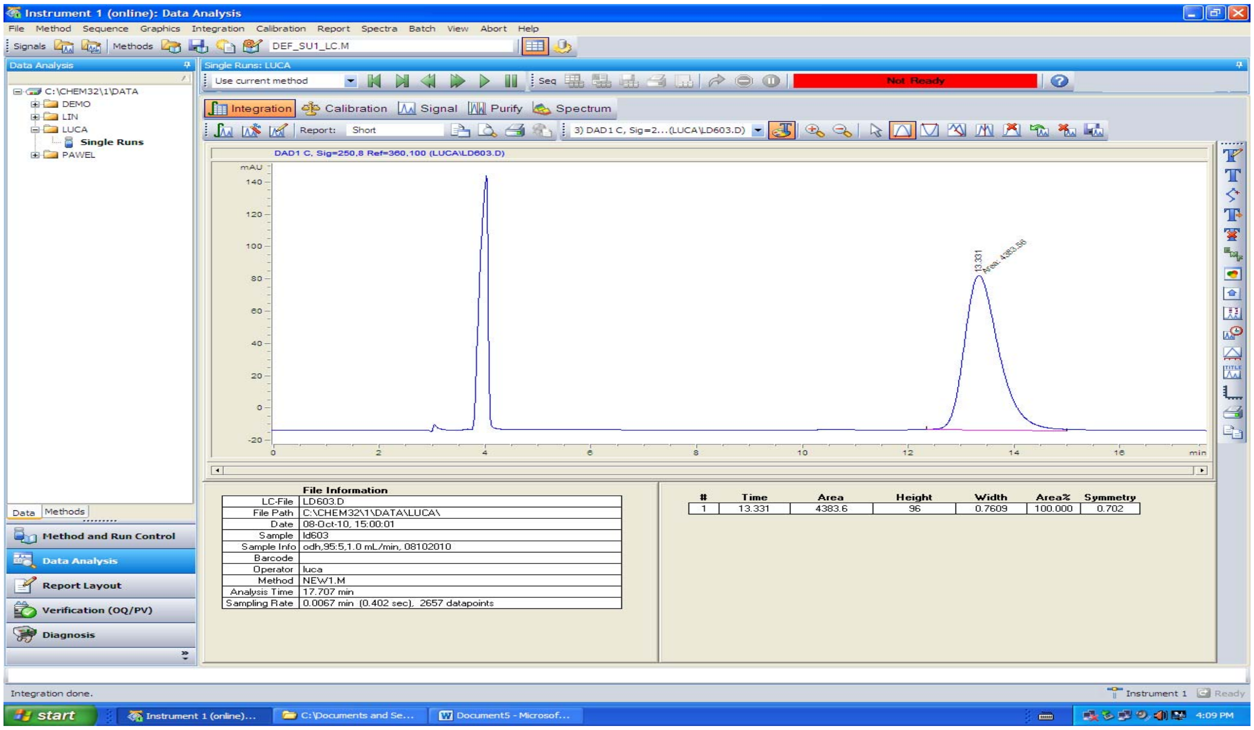
Instrument 1 (online): Data Analysis
DAD1 C, Sig=250,8 Ref=360,100 (LUCA\LD603.D)
13.331 Area: 4383.56
File Information
LC-File LD603.D
File Path C:\CHEM32\1\DATA\LUCA\
Date 08-Oct-10, 15:00:01
Sample ld603
Sample Info odh,95:5,1.0 mL/min, 08102010
Operator luca
Method NEW1.M
Analysis Time 17.707 min
Sampling Rate 0.0067 min (0.402 sec), 2657 datapoints
Time Area Height Width Area% Symmetry
1 13.331 4383.6 96 0.7609 100.000 0.702

ℓp 5P3

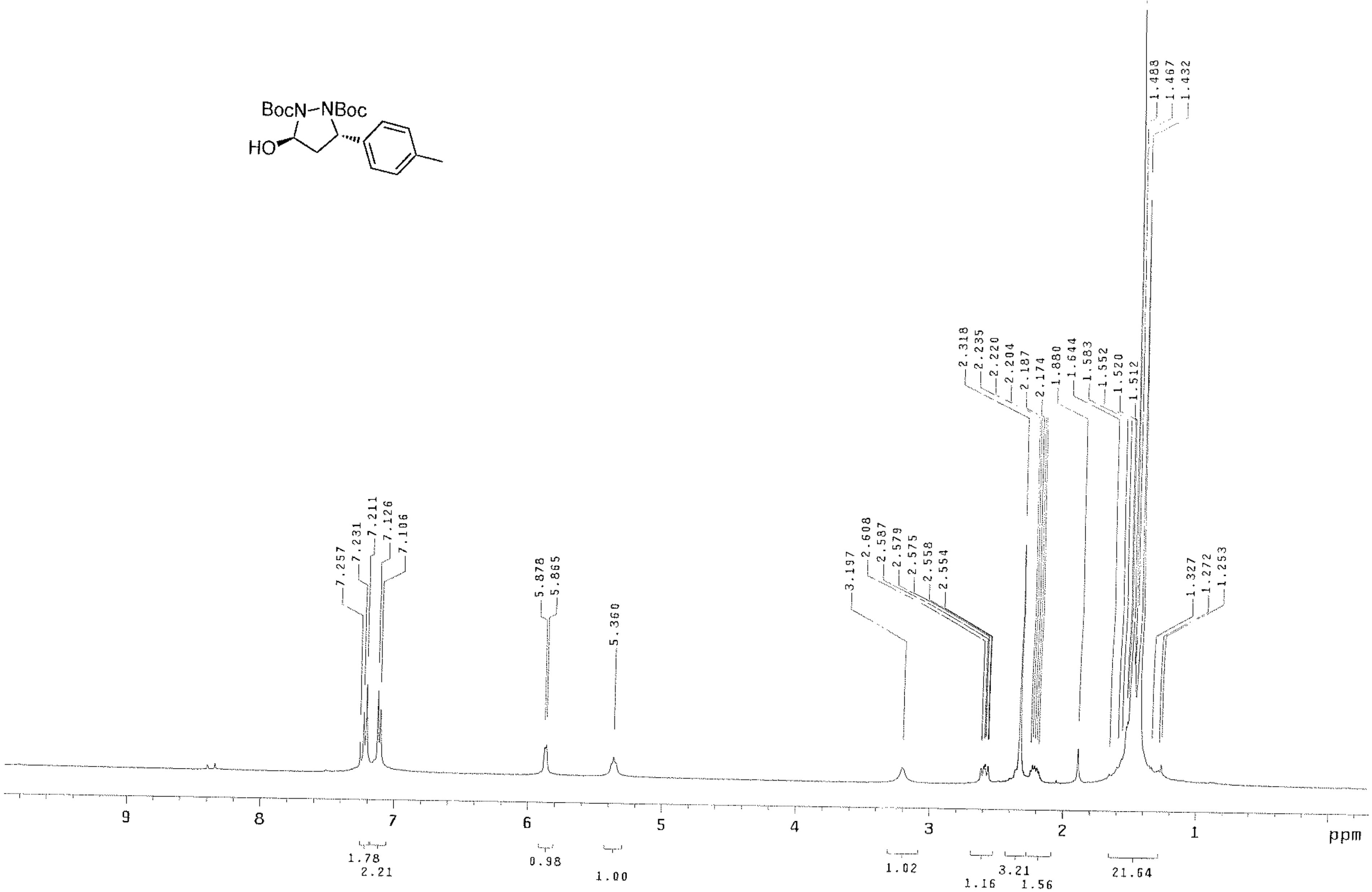
BocN
NBoc
HO
7.257
7.231
7.211
7.126
7.106
5.878
5.865
5.360
3.197
2.608
2.587
2.579
2.575
2.558
2.554
2.318
2.235
2.220
2.204
2.187
2.174
1.880
1.644
1.583
1.552
1.520
1.512
1.488
1.467
1.432
1.327
1.272
1.253
9
8
7
6
5
4
3
2
1
ppm
1.78
2.21
0.98
1.00
1.02
1.16
3.21
1.56
21.64

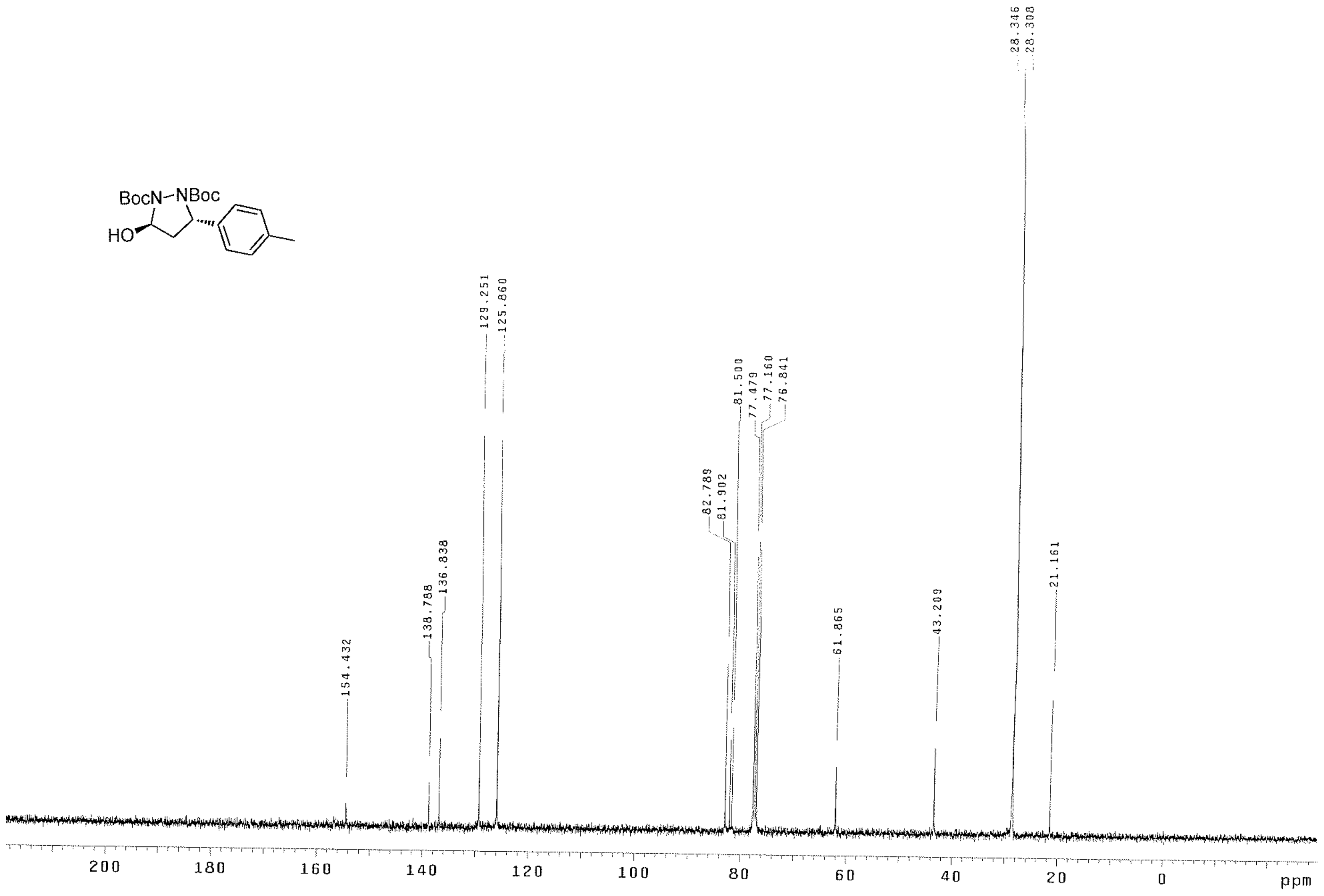
BocN–NBoc
HO
28.346
28.308
129.251
125.860
81.500
77.479
77.160
76.841
82.789
81.902
136.838
138.788
154.432
61.865
43.209
21.161
200
180
160
140
120
100
80
60
40
20
0
ppm

Display Report

Analysis Info

Analysis Name	H:\Data2\Luca\ld593b000001.d	Acquisition Date	2011-03-01 15:30:36
Method	tune_low_dirk.m	Operator	pia
Sample Name	ld593b	Instrument / Ser#	micrOTOF 125
Comment			

Acquisition Parameter

Source Type	ESI	Ion Polarity	Positive	Set Nebulizer	0.4 Bar
Focus	Not active			Set Dry Heater	170 °C
Scan Begin	50 m/z	Set Capillary	4500 V	Set Dry Gas	4.0 l/min
Scan End	3000 m/z	Set End Plate Offset	-500 V	Set Divert Valve	Source

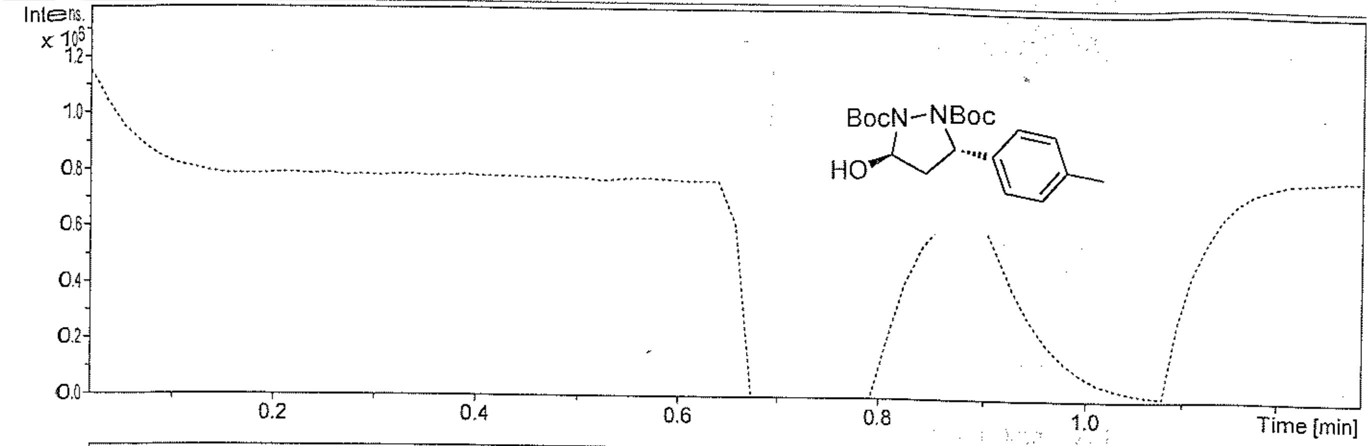

······ TIC +

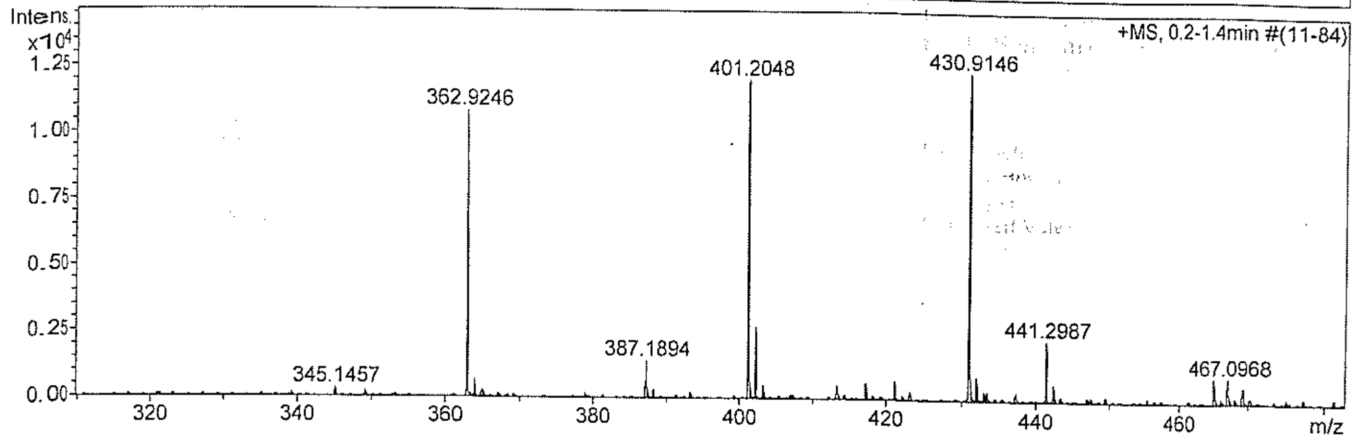

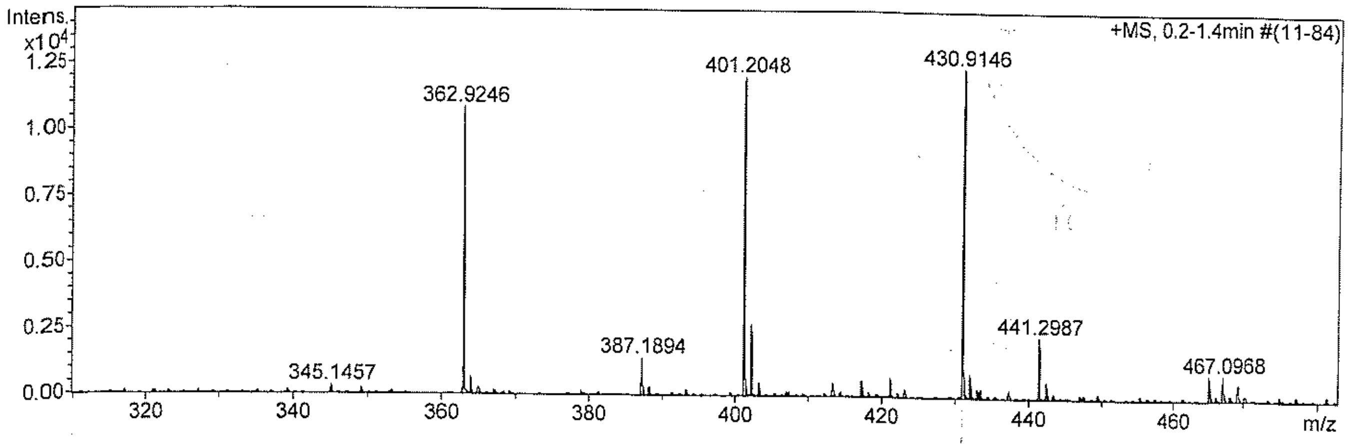

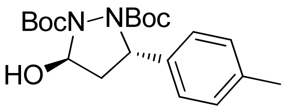
BocN
NBoc
HO

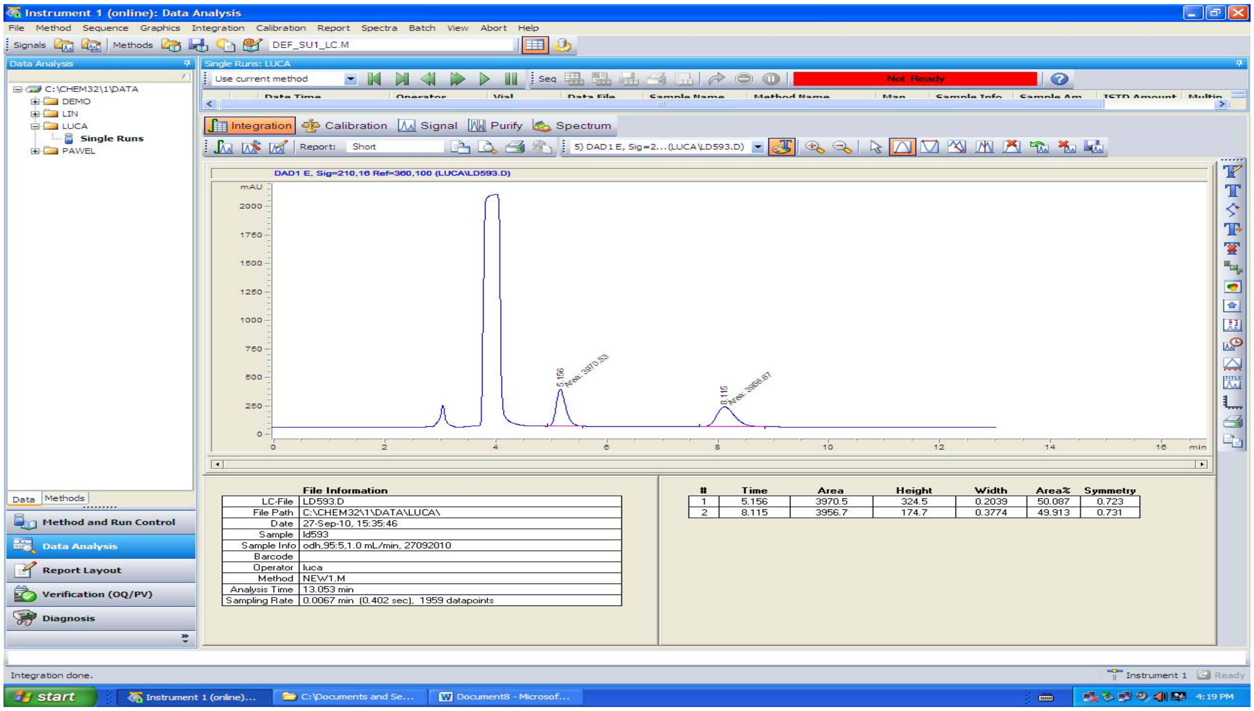
Instrument 1 (online): Data Analysis
DAD1 E, Sig=210,16 Ref=360,100 (LUCA\LD593.D)
5.156 Area: 3970.53
8.115 Area: 3956.67
File Information
LC-File LD593.D
File Path C:\CHEM32\1\DATA\LUCA\
Date 27-Sep-10, 15:35:46
Sample ld593
Sample Info odh,95:5,1.0 mL/min, 27092010
Operator luca
Method NEW1.M
Analysis Time 13.053 min
Sampling Rate 0.0067 min (0.402 sec), 1959 datapoints
Time Area Height Width Area% Symmetry
1 5.156 3970.5 324.5 0.2039 50.087 0.723
2 8.115 3956.7 174.7 0.3774 49.913 0.731
Integration done.

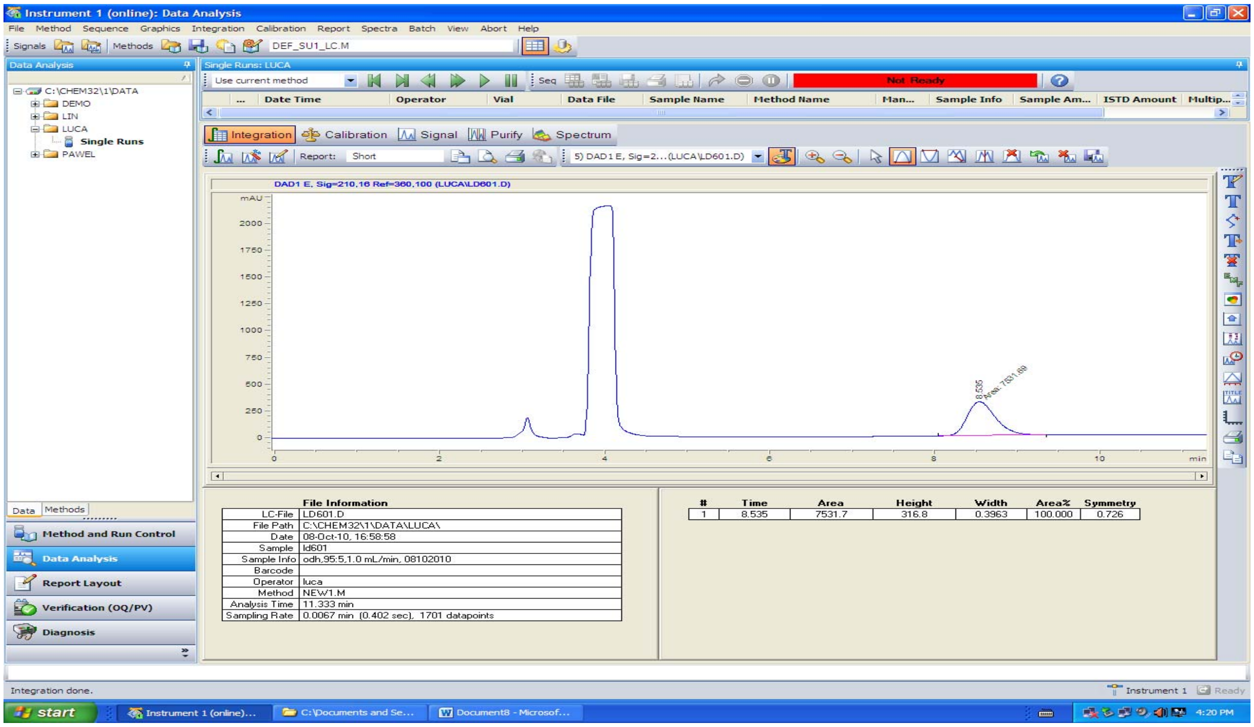
Instrument 1 (online): Data Analysis
DAD1 E, Sig=210,16 Ref=360,100 (LUCA\LD601.D)
8.535 Area: 7531.69
File Information
LC-File LD601.D
File Path C:\CHEM32\1\DATA\LUCA\
Date 08-Oct-10, 16:58:58
Sample ld601
Sample Info odh,95:5,1.0 mL/min, 08102010
Operator luca
Method NEW1.M
Analysis Time 11.333 min
Sampling Rate 0.0067 min (0.402 sec), 1701 datapoints
Time Area Height Width Area% Symmetry
1 8.535 7531.7 316.8 0.3963 100.000 0.726
Integration done.

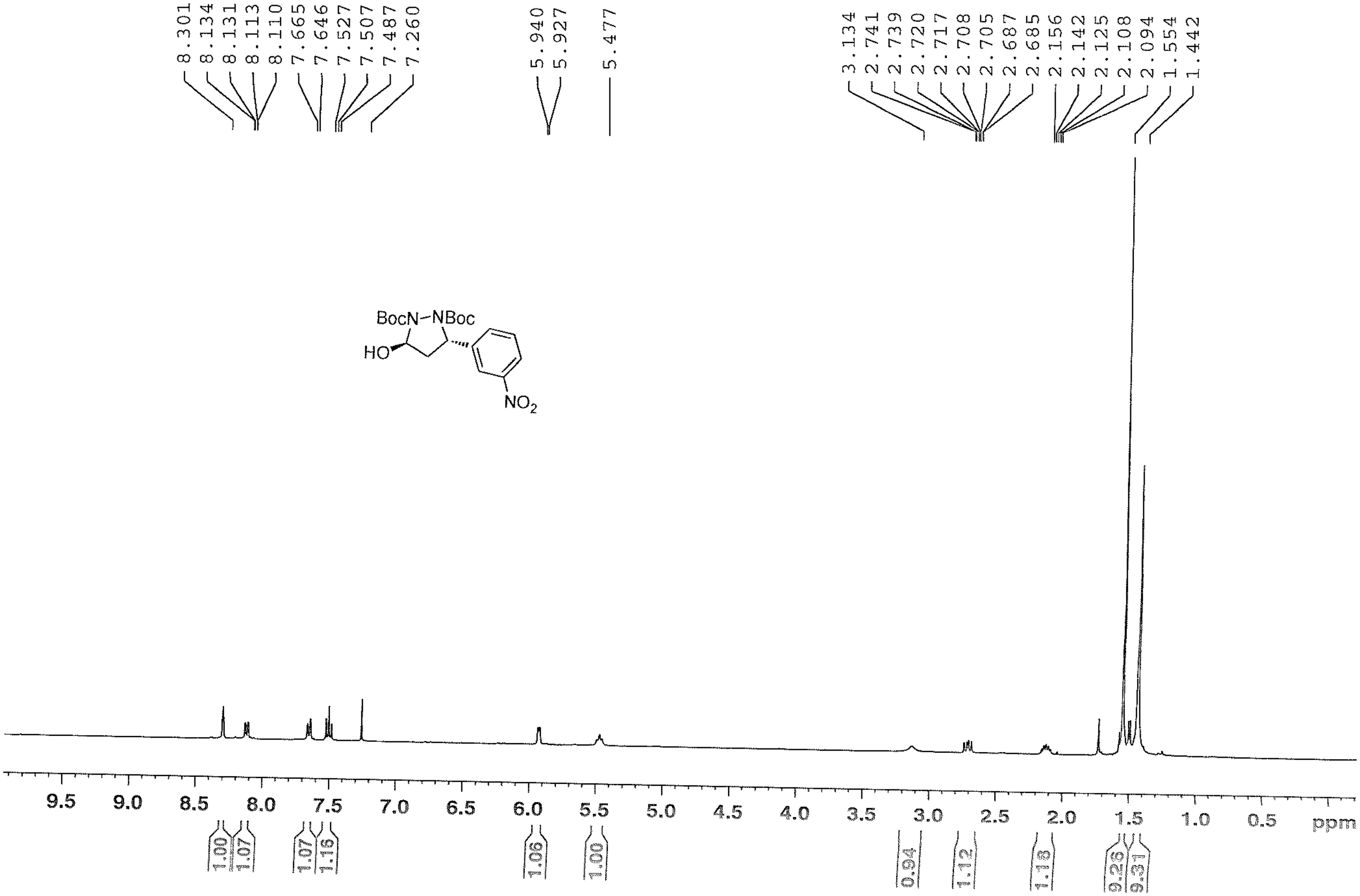
8.301
8.134
8.131
8.113
8.110
7.665
7.646
7.527
7.507
7.487
7.260
5.940
5.927
5.477
3.134
2.741
2.739
2.720
2.717
2.708
2.705
2.687
2.685
2.156
2.142
2.125
2.108
2.094
1.554
1.442
BocN–NBoc
HO
NO2
9.5
9.0
8.5
8.0
7.5
7.0
6.5
6.0
5.5
5.0
4.5
4.0
3.5
3.0
2.5
2.0
1.5
1.0
0.5
ppm
1.00
1.07
1.07
1.16
1.06
1.00
0.94
1.12
1.18
9.26
9.31

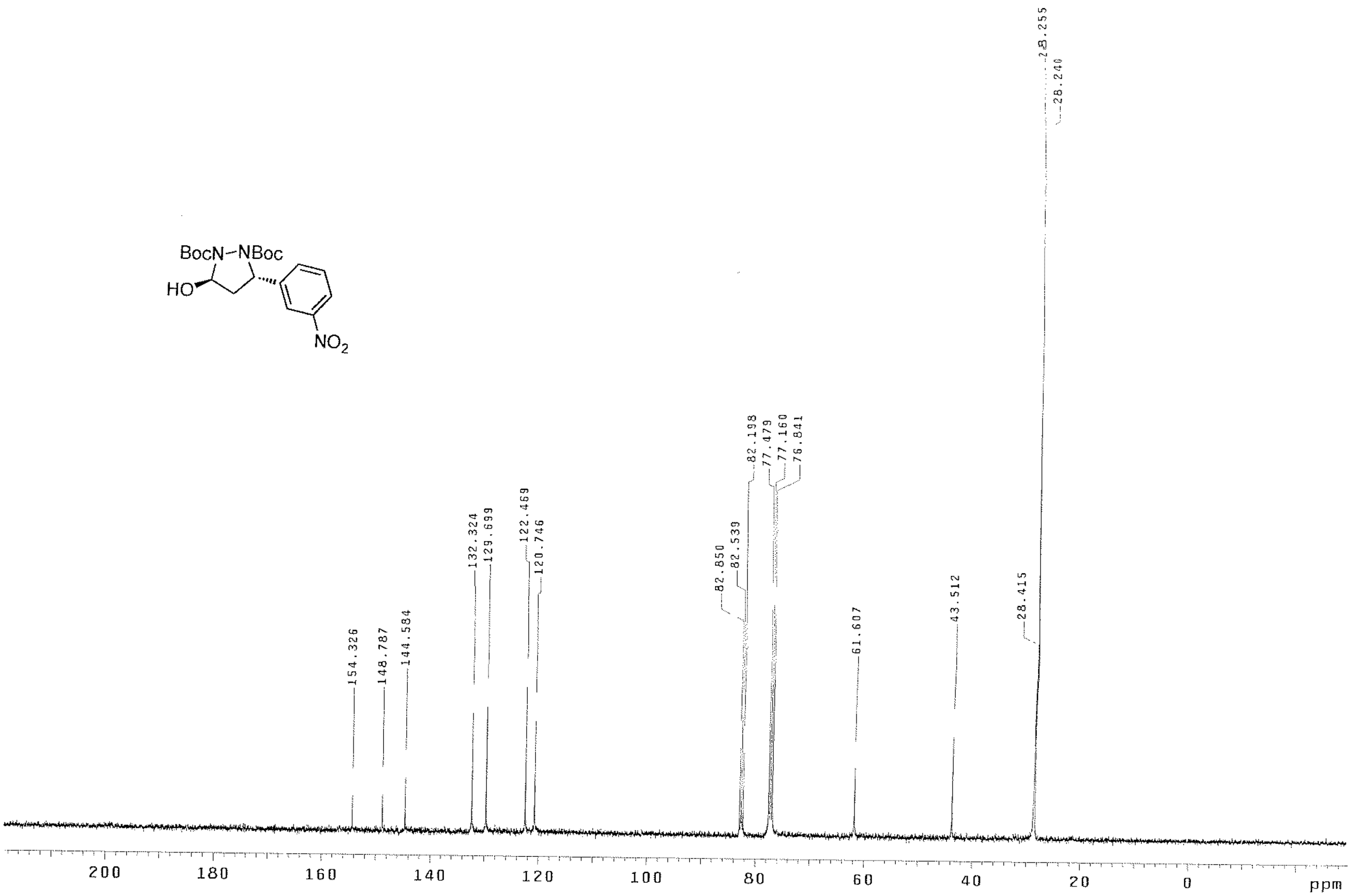

BocN–NBoc
HO
NO2
154.326
148.787
144.584
132.324
129.699
122.469
120.746
82.850
82.539
82.198
77.479
77.160
76.841
61.607
43.512
28.415
28.255
28.240
200
180
160
140
120
100
80
60
40
20
0
ppm

Display Report

Analysis Info

Analysis Name	H:\Data2\Luca\ld587000001.d	Acquisition Date	2011-03-01 13:26:55
Method	tune_low_dirk.m	Operator	pia
Sample Name	ld587	Instrument / Ser#	micrOTOF 125
Comment			

Acquisition Parameter

Source Type	ESI	Ion Polarity	Positive	Set Nebulizer	0.4 Bar
Focus	Not active			Set Dry Heater	170 °C
Scan Begin	50 m/z	Set Capillary	4500 V	Set Dry Gas	4.0 l/min
Scan End	3000 m/z	Set End Plate Offset	-500 V	Set Divert Valve	Source

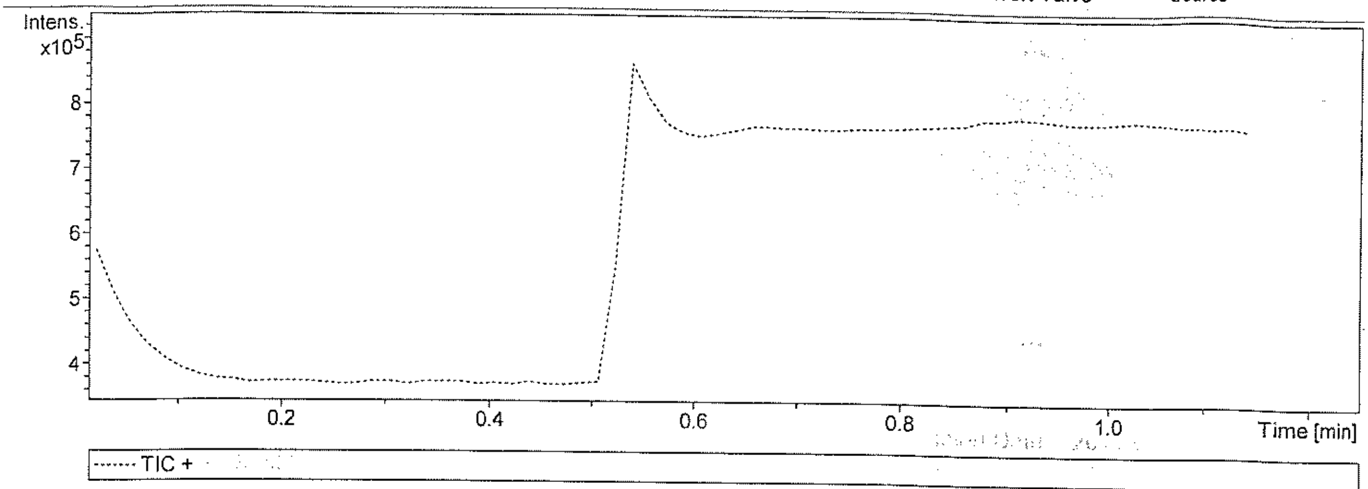

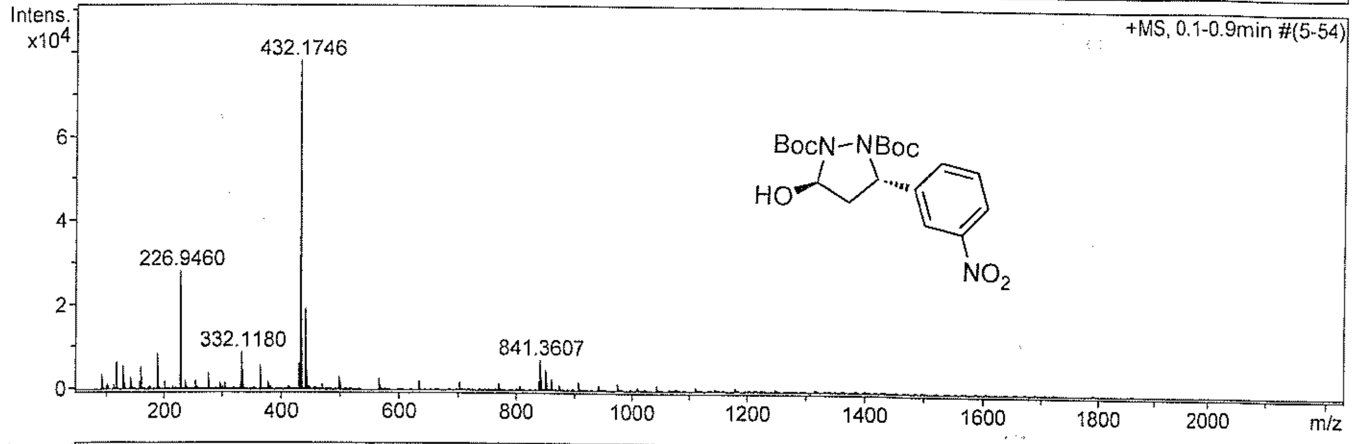

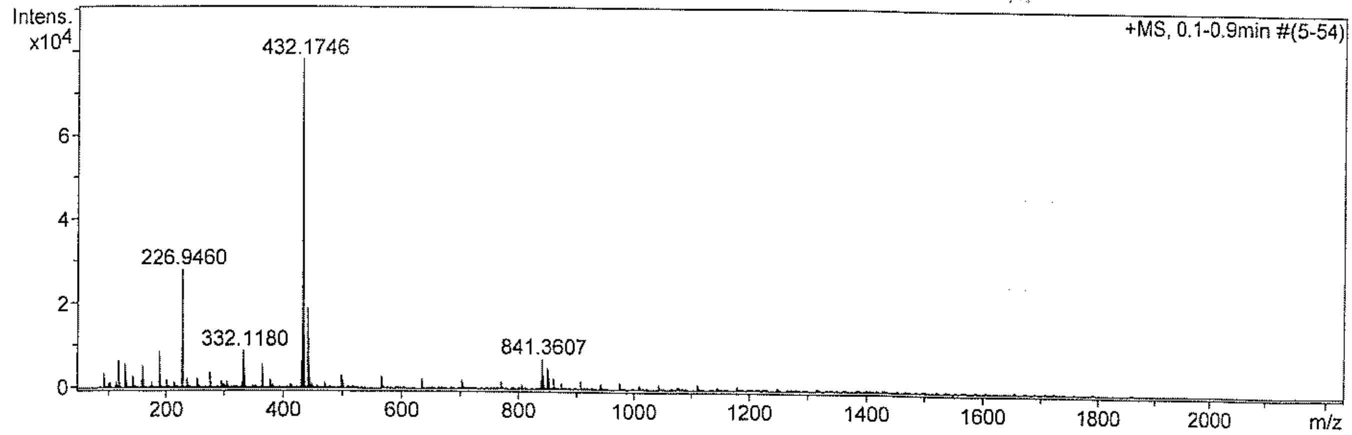

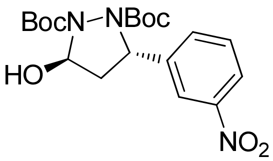
BocN
NBoc
HO
NO2

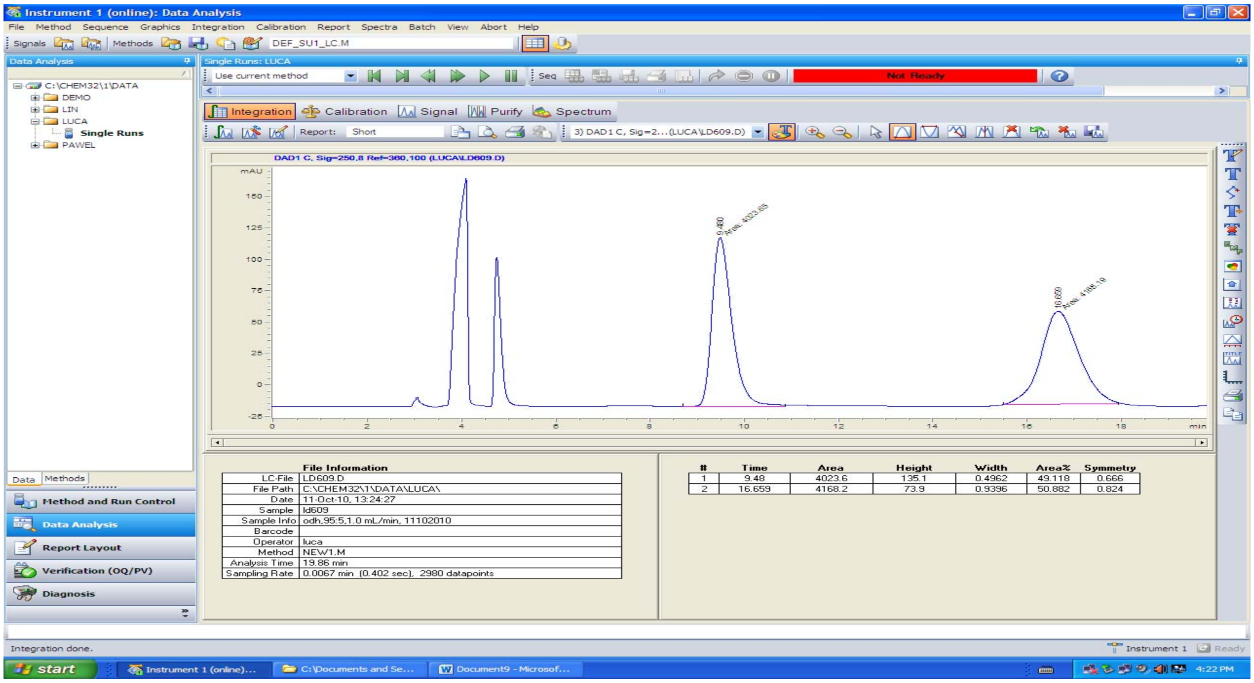
Instrument 1 (online): Data Analysis
DAD1 C, Sig=250,8 Ref=360,100 (LUCA\LD609.D)
9.480 Area: 4023.65
16.659 Area: 4168.19
File Information
LC-File LD609.D
File Path C:\CHEM32\1\DATA\LUCA\
Date 11-Oct-10, 13:24:27
Sample ld609
Sample Info odh,95:5,1.0 mL/min, 11102010
Operator luca
Method NEW1.M
Analysis Time 19.86 min
Sampling Rate 0.0067 min (0.402 sec), 2980 datapoints
Time Area Height Width Area% Symmetry
1 9.48 4023.6 135.1 0.4962 49.118 0.666
2 16.659 4168.2 73.9 0.9396 50.882 0.824

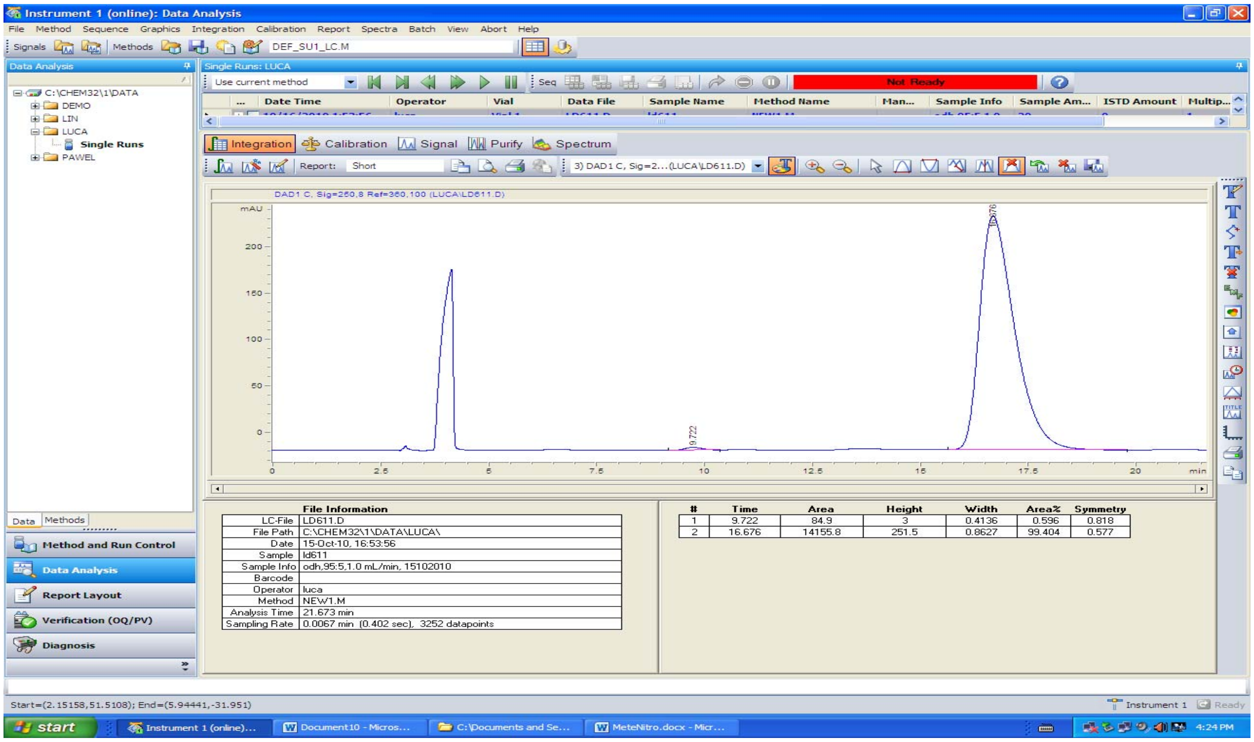
Instrument 1 (online): Data Analysis
DAD1 C, Sig=250,8 Ref=360,100 (LUCA\LD611.D)
9.722
16.676
File Information
LC-File LD611.D
File Path C:\CHEM32\1\DATA\LUCA\
Date 15-Oct-10, 16:53:56
Sample ld611
Sample Info odh,95:5,1.0 mL/min, 15102010
Operator luca
Method NEW1.M
Analysis Time 21.673 min
Sampling Rate 0.0067 min (0.402 sec), 3252 datapoints
Time Area Height Width Area% Symmetry
1 9.722 84.9 3 0.4136 0.596 0.818
2 16.676 14155.8 251.5 0.8627 99.404 0.577

LD 997
ALD

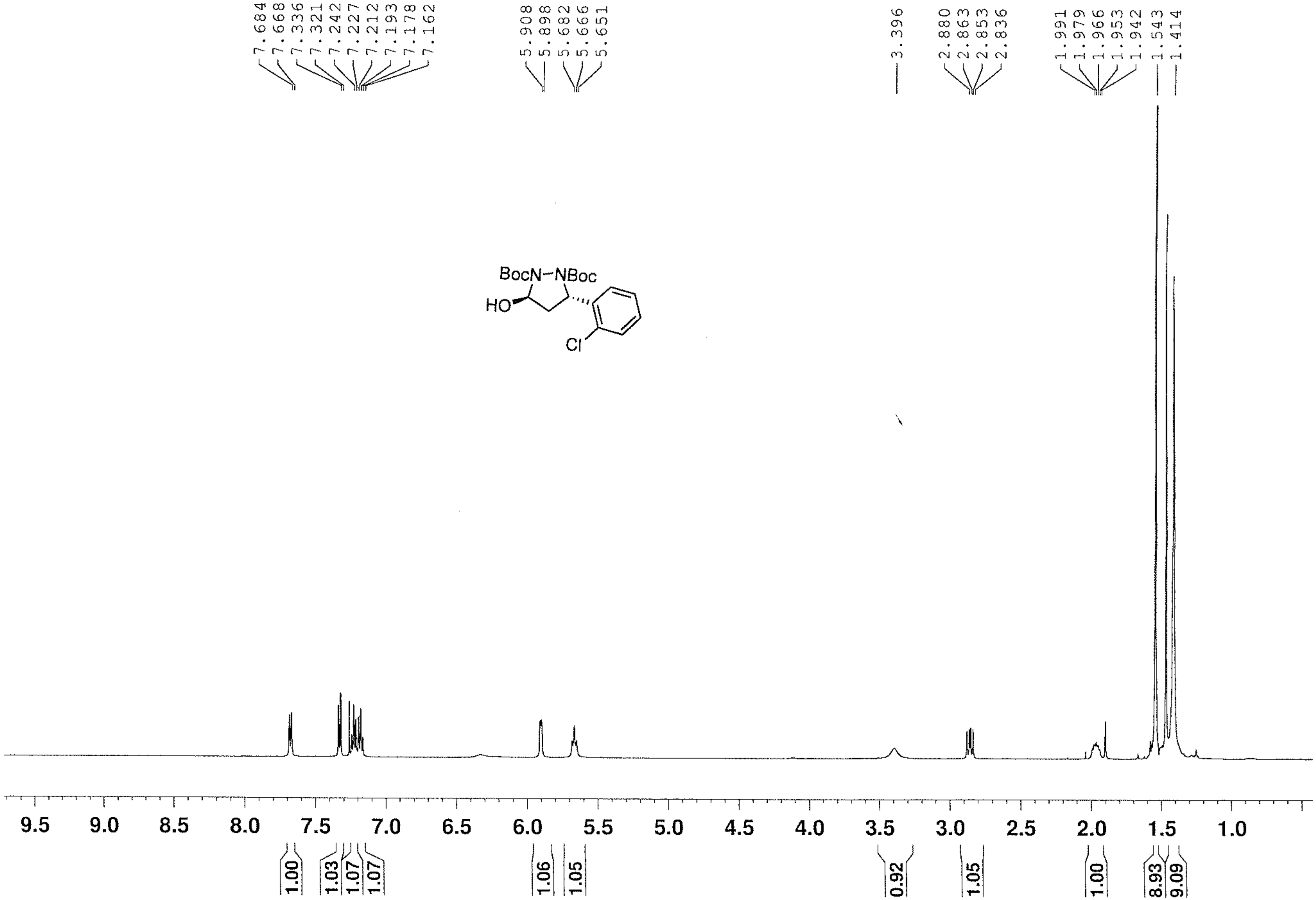

7.684
7.668
7.336
7.321
7.242
7.227
7.212
7.193
7.178
7.162
5.908
5.898
5.682
5.666
5.651
3.396
2.880
2.863
2.853
2.836
1.991
1.979
1.966
1.953
1.942
1.543
1.414
BocN–NBoc
HO
Cl
9.5
9.0
8.5
8.0
7.5
7.0
6.5
6.0
5.5
5.0
4.5
4.0
3.5
3.0
2.5
2.0
1.5
1.0
1.00
1.03
1.07
1.07
1.06
1.05
0.92
1.05
1.00
8.93
9.09

997C2

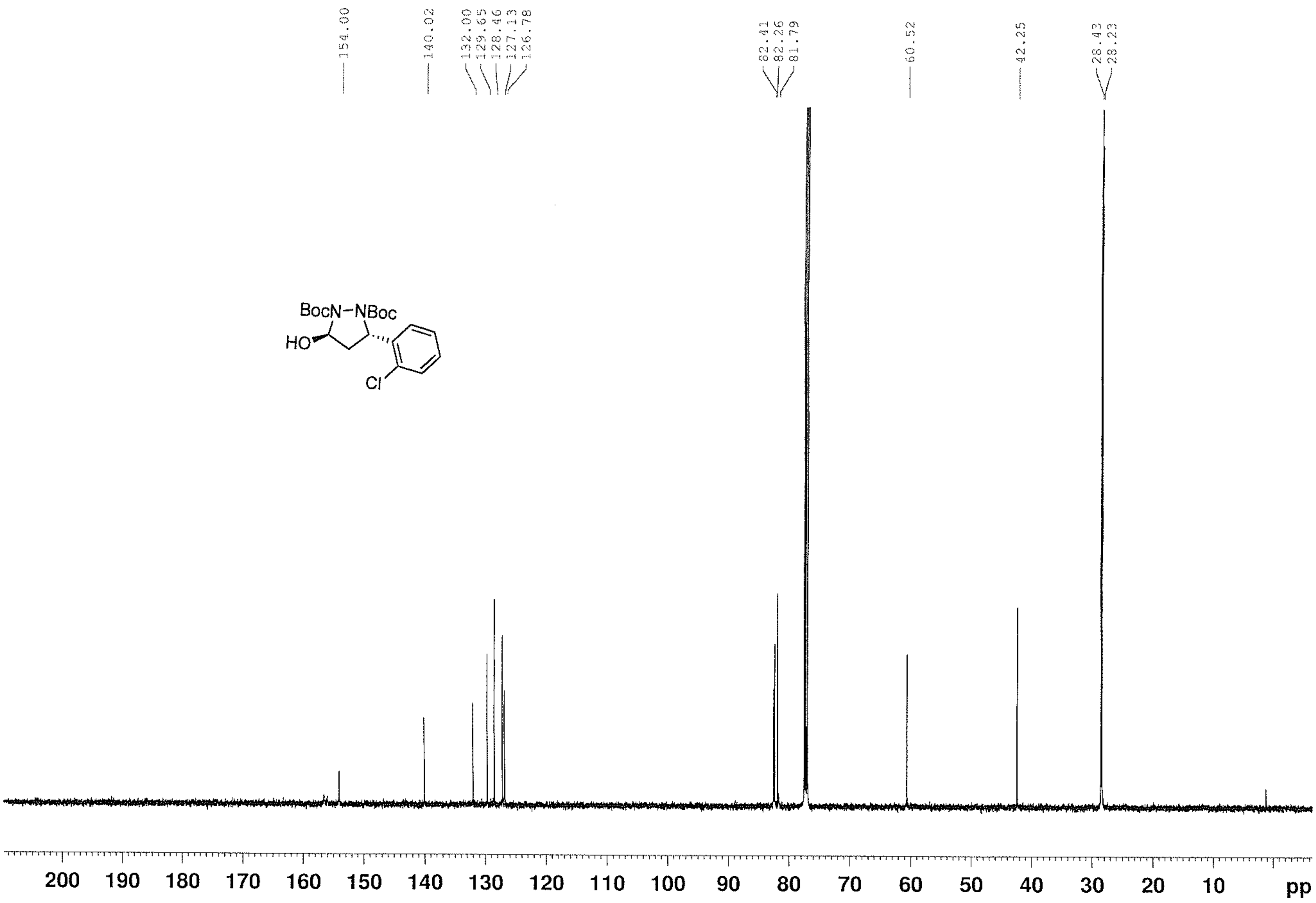
154.00
140.02
132.00
129.65
128.46
127.13
126.78
82.41
82.26
81.79
60.52
42.25
28.43
28.23
BocN–NBoc
HO
Cl
200
190
180
170
160
150
140
130
120
110
100
90
80
70
60
50
40
30
20
10
pp

Display Report

Analysis Info

Analysis Name	H:\Data2\Luca\ld995000001.d	Acquisition Date	2012-01-19 15:36:21
Method	tune_low_dirk.m	Operator	pia
Sample Name	ld995	Instrument / Ser#	micrOTOF 125
Comment			

Acquisition Parameter

Source Type	ESI	Ion Polarity	Positive	Set Nebulizer	0.4 Bar
Focus	Not active			Set Dry Heater	170 °C
Scan Begin	50 m/z	Set Capillary	4500 V	Set Dry Gas	4.0 l/min
Scan End	3000 m/z	Set End Plate Offset	-500 V	Set Divert Valve	Source

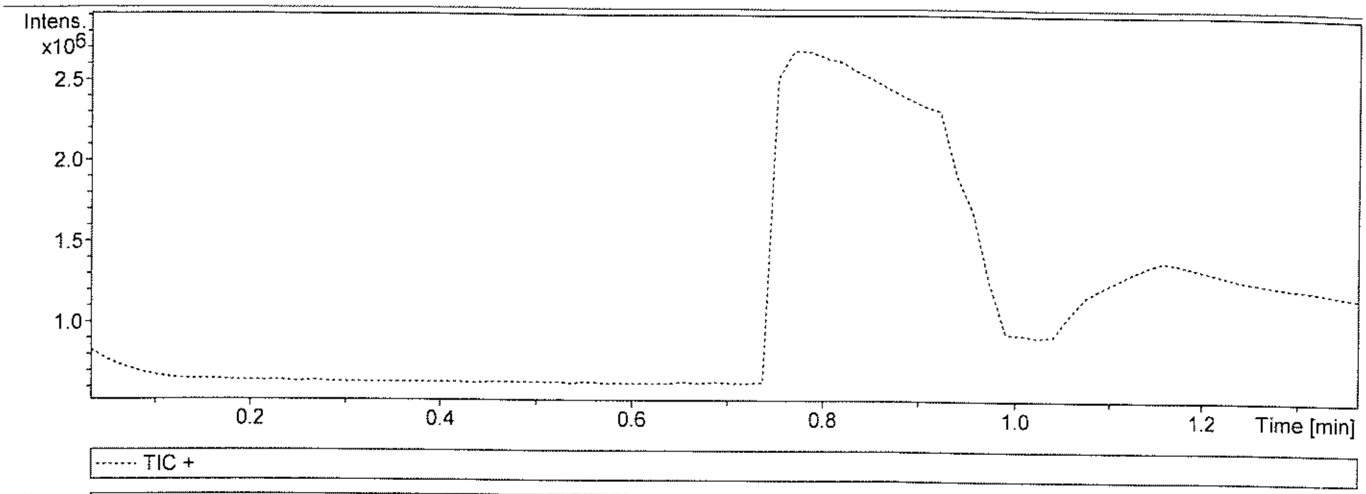

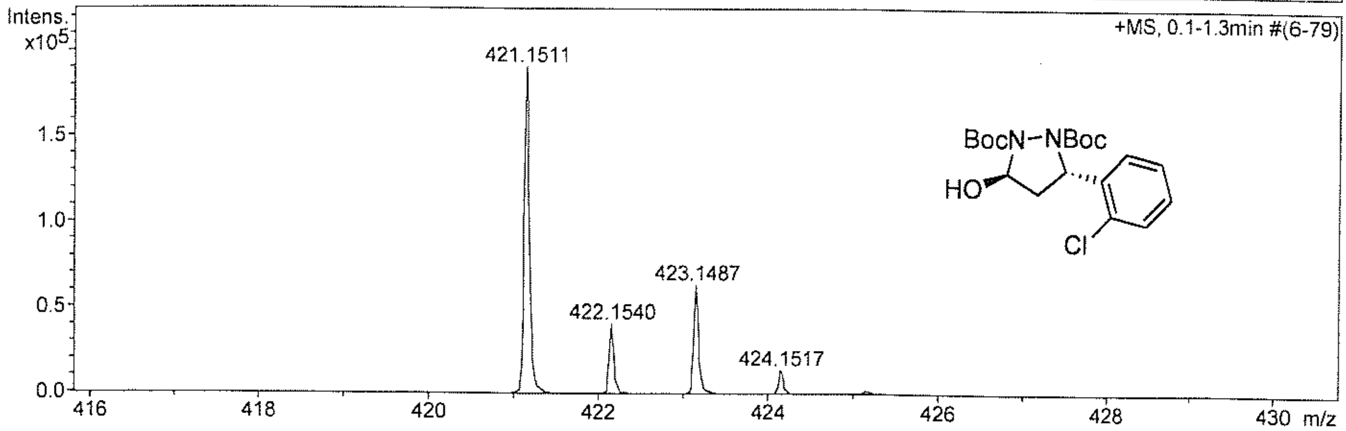

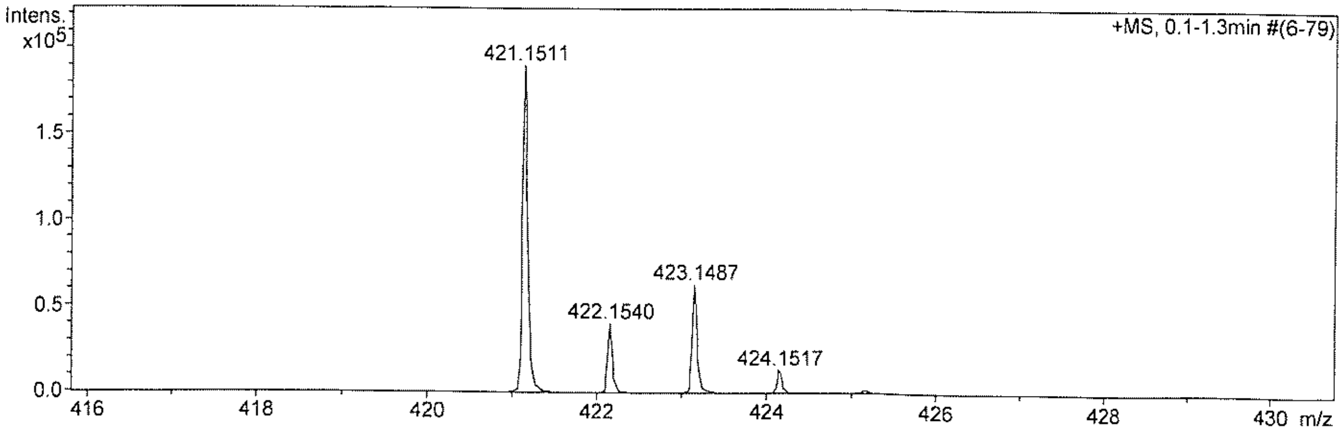

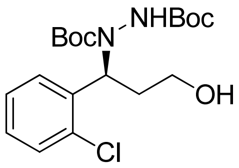
BocN
NHBoc
OH
Cl

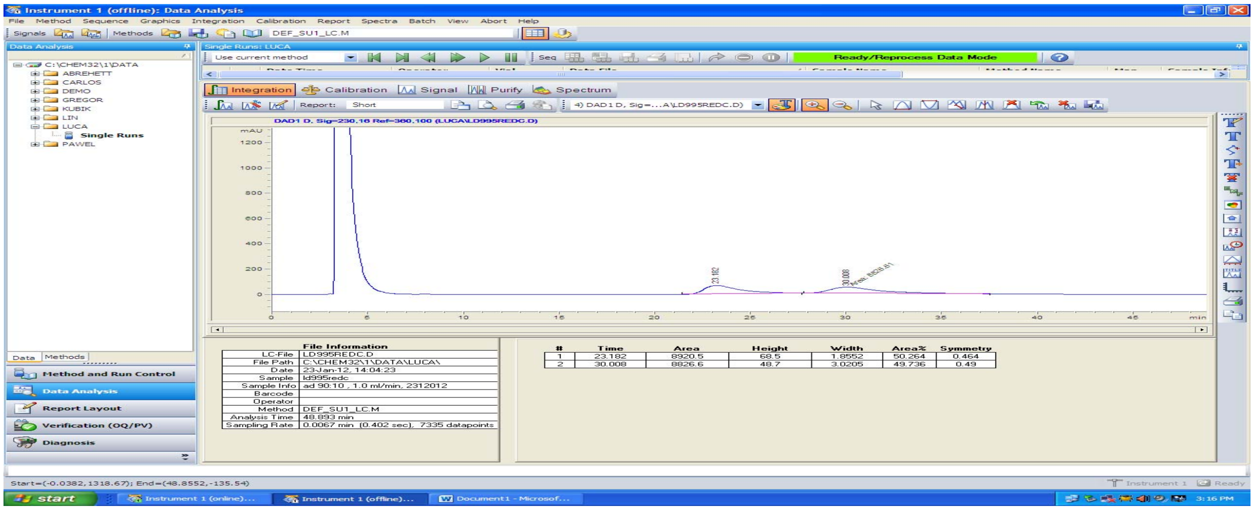
Instrument 1 (offline): Data Analysis
DAD1 D, Sig=230,16 Ref=360,100 (LUCA\LD995REDC.D)
23.182
30.008
Area: 8826.61
File Information
LC-File LD995REDC.D
File Path C:\CHEM32\1\DATA\LUCA\
Date 23-Jan-12, 14:04:23
Sample ld995redc
Sample Info ad 90:10 , 1.0 ml/min, 2312012
Method DEF_SU1_LC.M
Analysis Time 48.893 min
Sampling Rate 0.0067 min (0.402 sec), 7335 datapoints
Time Area Height Width Area% Symmetry
1 23.182 8920.5 68.5 1.8552 50.264 0.464
2 30.008 8826.6 48.7 3.0205 49.736 0.49

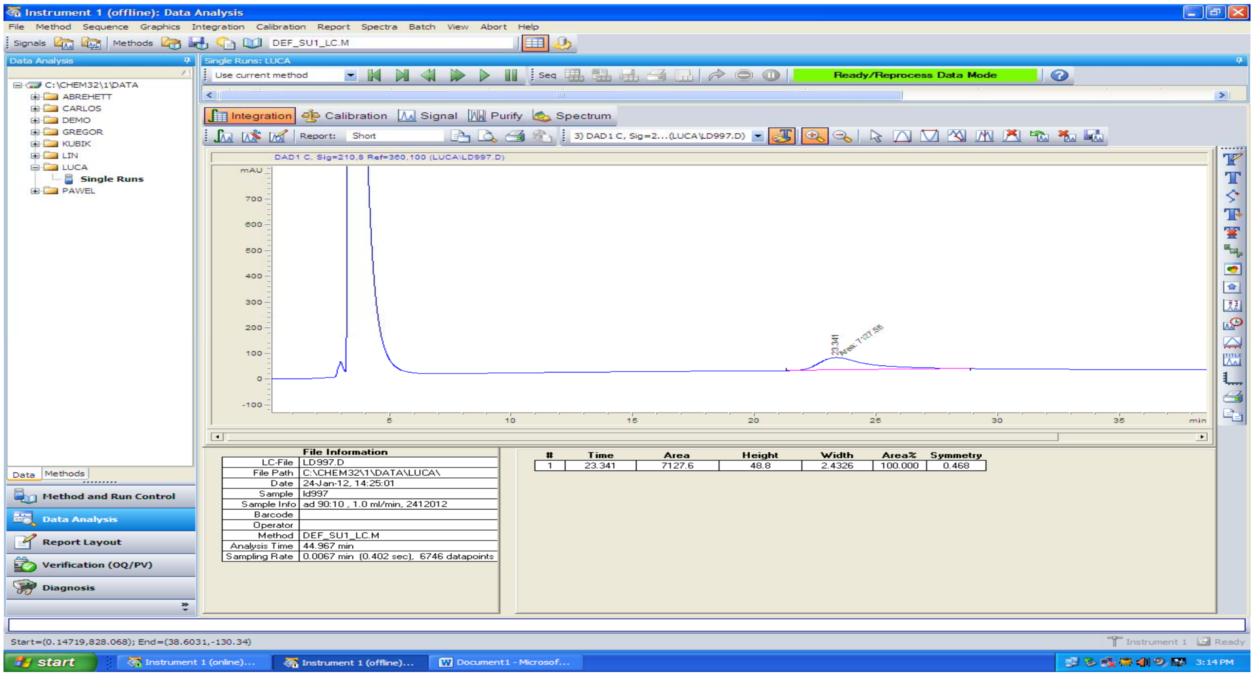
Instrument 1 (offline): Data Analysis
DAD1 C, Sig=210,8 Ref=360,100 (LUCA\LD997.D)
23.341
Area: 7127.58
File Information
LC-File LD997.D
File Path C:\CHEM32\1\DATA\LUCA\
Date 24-Jan-12, 14:25:01
Sample ld997
Sample Info ad 90:10 , 1.0 ml/min, 2412012
Method DEF_SU1_LC.M
Analysis Time 44.967 min
Sampling Rate 0.0067 min (0.402 sec), 6746 datapoints
Time Area Height Width Area% Symmetry
1 23.341 7127.6 48.8 2.4326 100.000 0.468

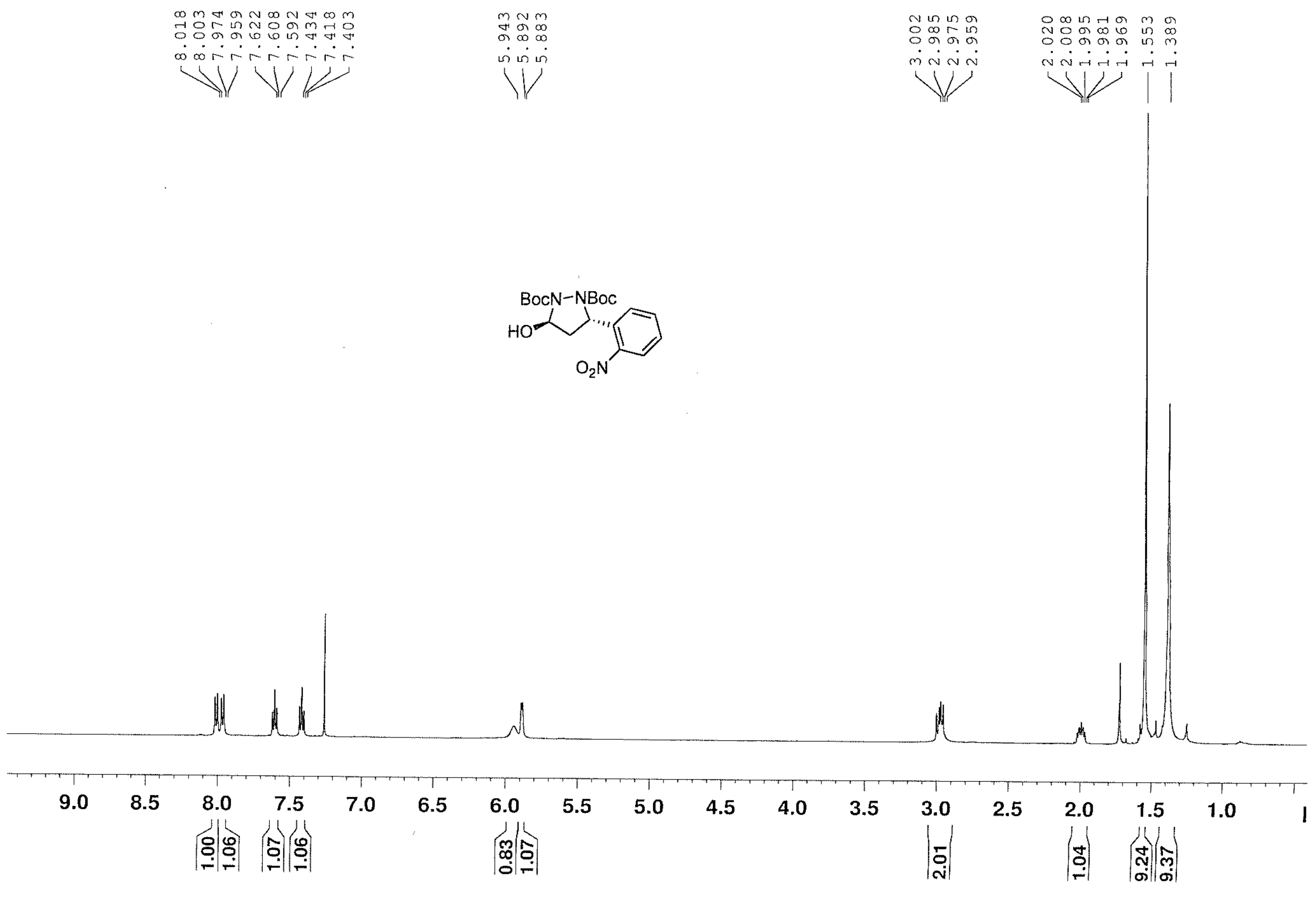
8.018
8.003
7.974
7.959
7.622
7.608
7.592
7.434
7.418
7.403
5.943
5.892
5.883
3.002
2.985
2.975
2.959
2.020
2.008
1.995
1.981
1.969
1.553
1.389
BocN–NBoc
HO
O2N
9.0
8.5
8.0
7.5
7.0
6.5
6.0
5.5
5.0
4.5
4.0
3.5
3.0
2.5
2.0
1.5
1.0
1.00
1.06
1.07
1.06
0.83
1.07
2.01
1.04
9.24
9.37

996C

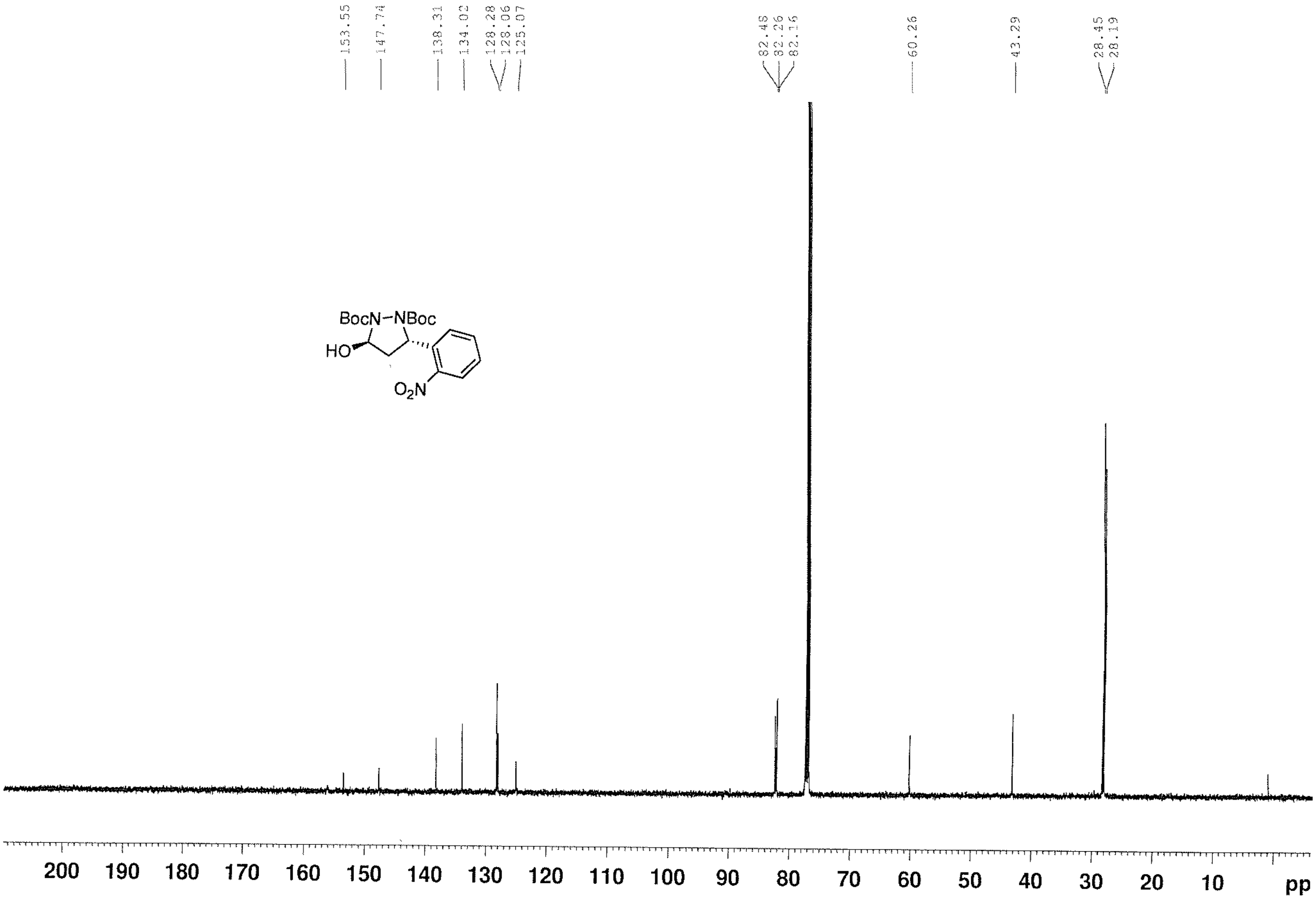

153.55
147.74
138.31
134.02
128.28
128.06
125.07
82.45
82.26
82.16
60.26
43.29
28.45
28.19
BocN–NBoc
HO
O2N
200
190
180
170
160
150
140
130
120
110
100
90
80
70
60
50
40
30
20
10
pp

Display Report

Analysis Info

Analysis Name	H:\Data2\Luca\ld996000001.d	Acquisition Date	2012-01-19 15:43:15
Method	tune_low_dirk.m	Operator	pia
Sample Name	ld996	Instrument / Ser#	micrOTOF 125
Comment			

Acquisition Parameter

Source Type	ESI	Ion Polarity	Positive	Set Nebulizer	0.4 Bar
Focus	Not active			Set Dry Heater	170 °C
Scan Begin	50 m/z	Set Capillary	4500 V	Set Dry Gas	4.0 l/min
Scan End	3000 m/z	Set End Plate Offset	-500 V	Set Divert Valve	Source

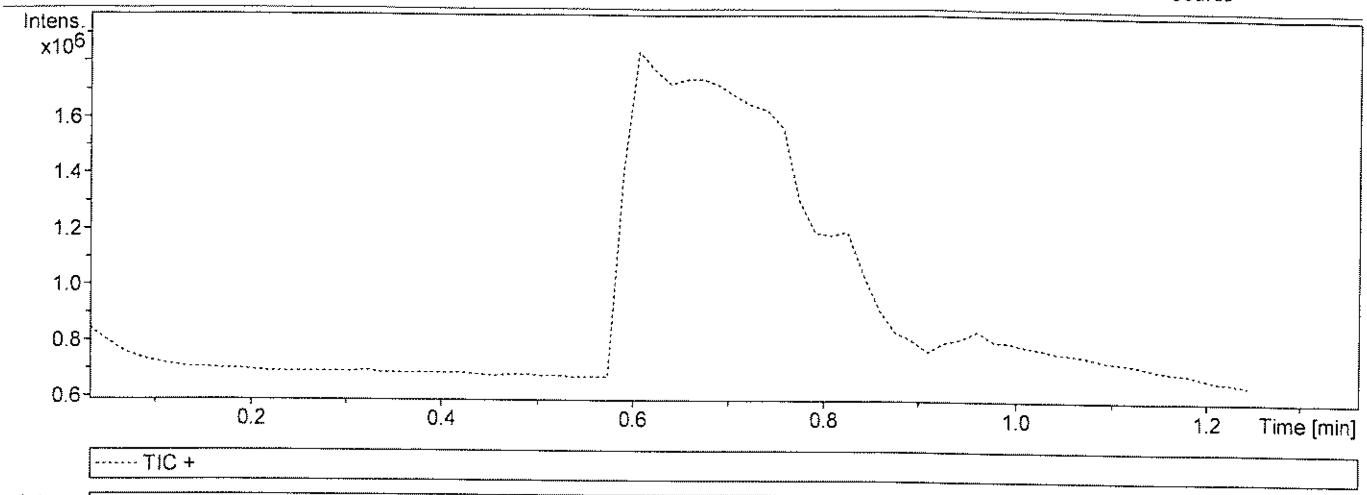

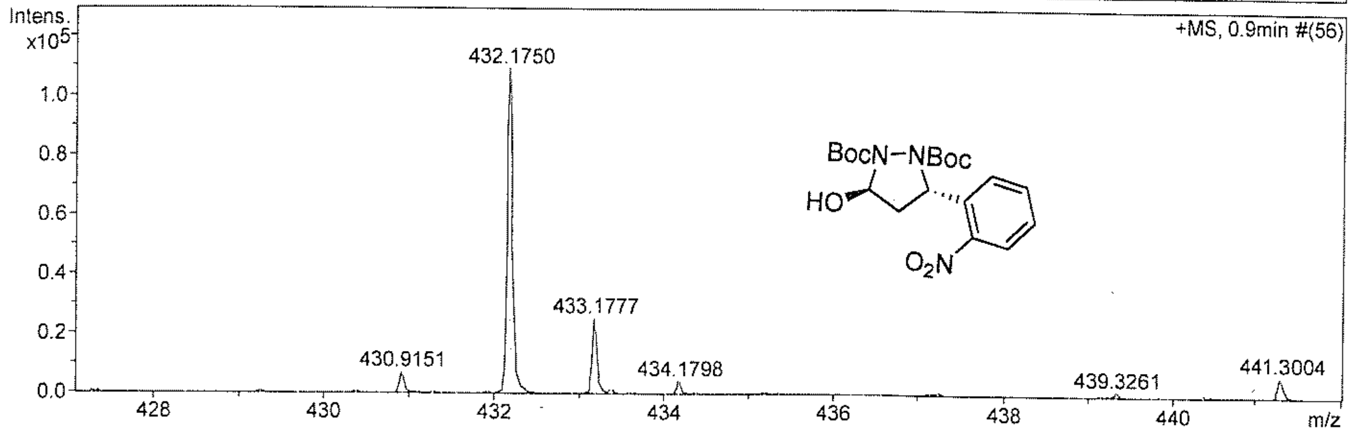

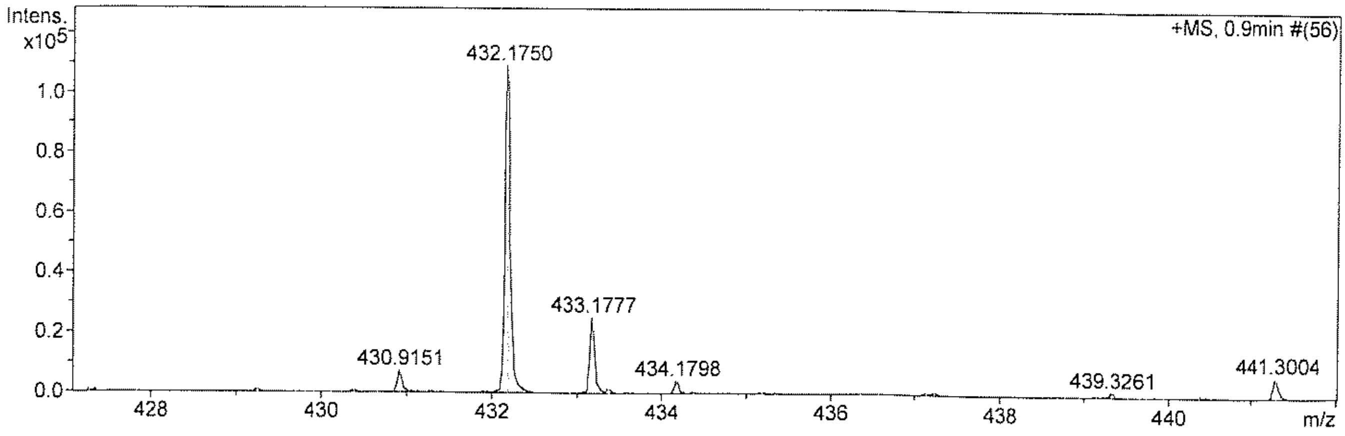

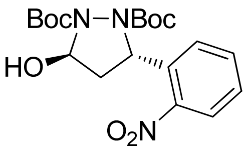

BocN−NBoc
HO
O_2N

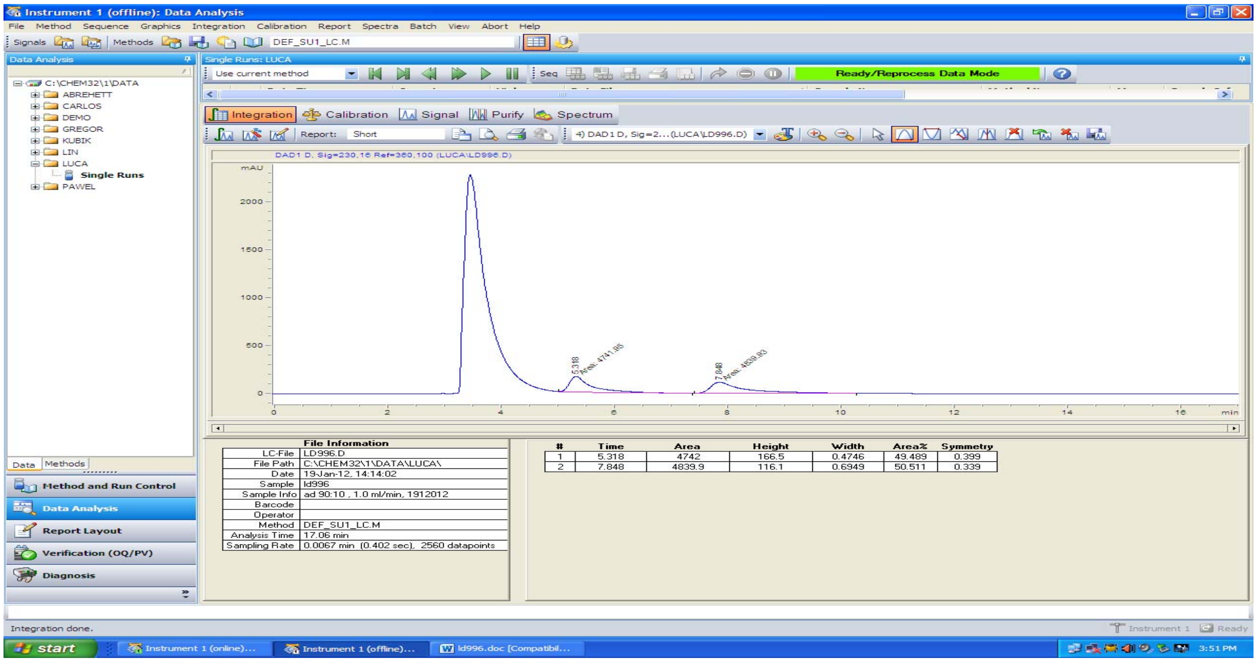

Instrument 1 (offline): Data Analysis
DAD1 D, Sig=230,16 Ref=360,100 (LUCA\LD996.D)
5.318
Area: 4741.95
7.848
Area: 4839.93
File Information
LC-File LD996.D
File Path C:\CHEM32\1\DATA\LUCA\
Date 19-Jan-12, 14:14:02
Sample ld996
Sample Info ad 90:10 , 1.0 ml/min, 1912012
Method DEF_SU1_LC.M
Analysis Time 17.06 min
Sampling Rate 0.0067 min (0.402 sec), 2560 datapoints
Time Area Height Width Area% Symmetry
1 5.318 4742 166.5 0.4746 49.489 0.399
2 7.848 4839.9 116.1 0.6949 50.511 0.339

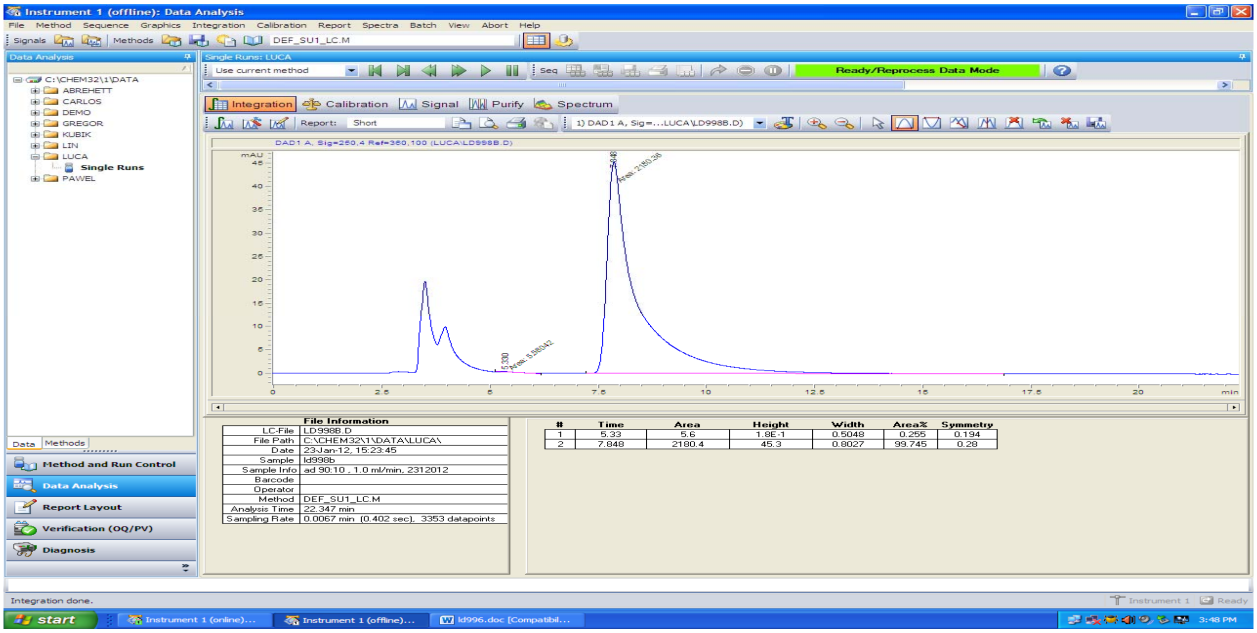

Instrument 1 (offline): Data Analysis
DAD1 A, Sig=250,4 Ref=360,100 (LUCA\LD998B.D)
5.330
Area: 5.58042
Area: 2180.38
File Information
LC-File LD998B.D
File Path C:\CHEM32\1\DATA\LUCA\
Date 23-Jan-12, 15:23:45
Sample ld998b
Sample Info ad 90:10 , 1.0 ml/min, 2312012
Method DEF_SU1_LC.M
Analysis Time 22.347 min
Sampling Rate 0.0067 min (0.402 sec), 3353 datapoints
Time Area Height Width Area% Symmetry
1 5.33 5.6 1.8E-1 0.5048 0.255 0.194
2 7.848 2180.4 45.3 0.8027 99.745 0.28

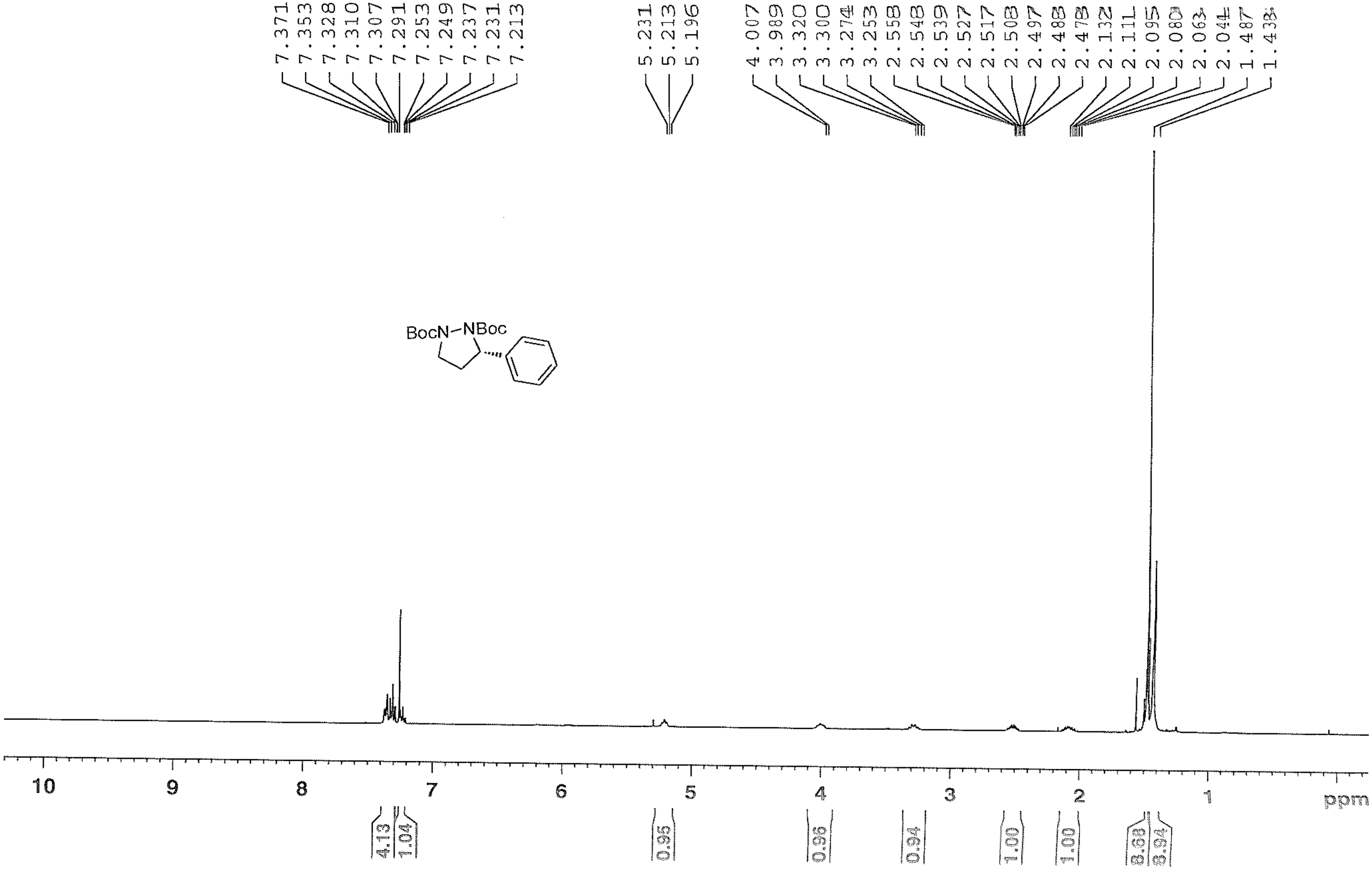
7.371
7.353
7.328
7.310
7.307
7.291
7.253
7.249
7.237
7.231
7.213
5.231
5.213
5.196
4.007
3.989
3.320
3.300
3.274
3.253
2.558
2.548
2.539
2.527
2.517
2.508
2.497
2.488
2.478
2.132
2.111
2.095
2.080
2.063
2.044
1.487
1.438
BocN–NBoc
10
9
8
7
6
5
4
3
2
1
ppm
4.13
1.04
0.95
0.96
0.94
1.00
1.00
8.68
8.94

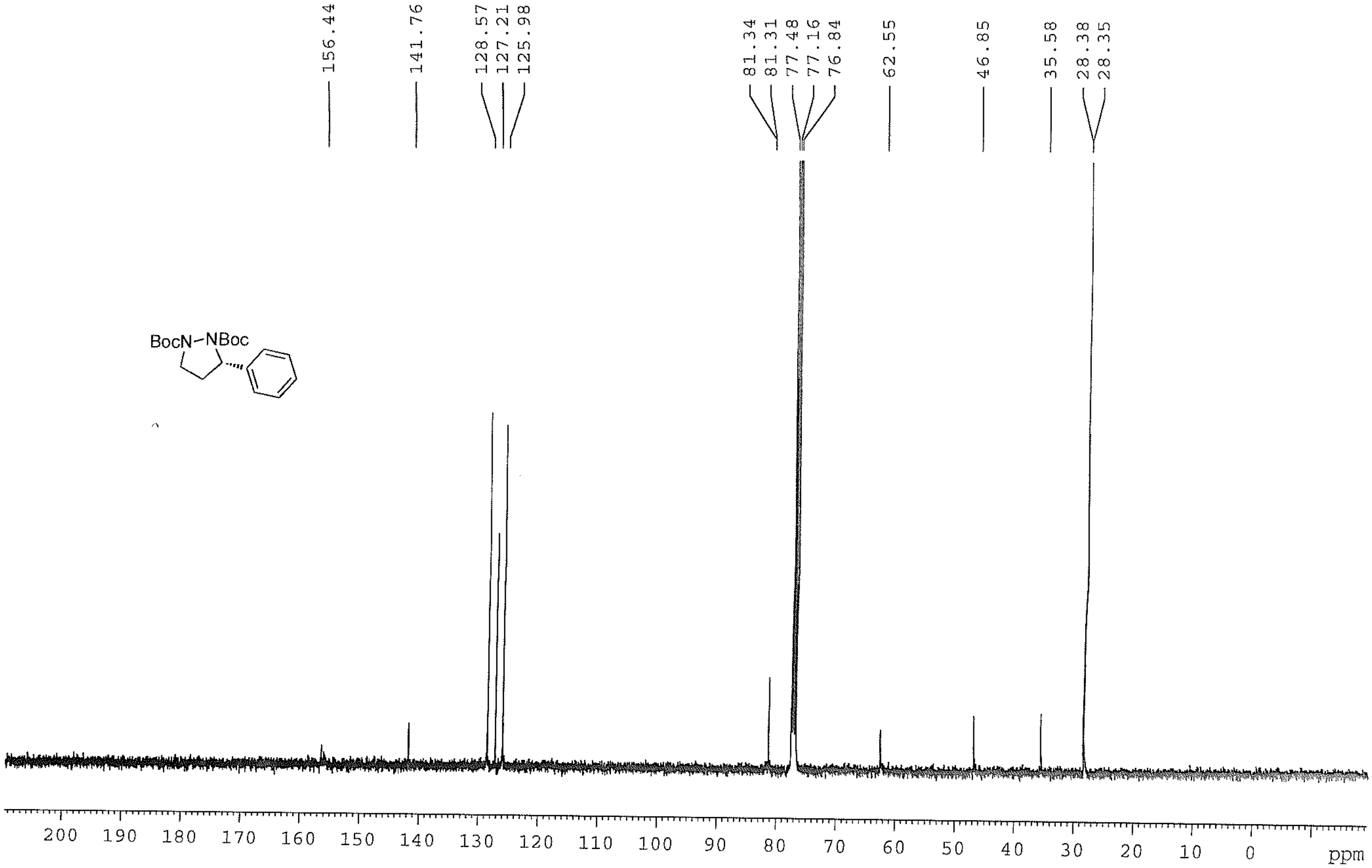
156.44
141.76
128.57
127.21
125.98
81.34
81.31
77.48
77.16
76.84
62.55
46.85
35.58
28.38
28.35
BocN–NBoc
200 190 180 170 160 150 140 130 120 110 100 90 80 70 60 50 40 30 20 10 0
ppm

Display Report

Analysis Info

Analysis Name	H:\Data2\Luca\ld734000001.d
Method	tune_low_dirk.m
Sample Name	ld734
Comment	

Acquisition Date	2011-03-03 15:49:20
Operator	pia
Instrument / Ser#	micrOTOF 125

Acquisition Parameter

Source Type	ESI	Ion Polarity	Positive	Set Nebulizer	0.4 Bar
Focus	Not active			Set Dry Heater	170 °C
Scan Begin	50 m/z	Set Capillary	4500 V	Set Dry Gas	4.0 l/min
Scan End	3000 m/z	Set End Plate Offset	-500 V	Set Divert Valve	Source

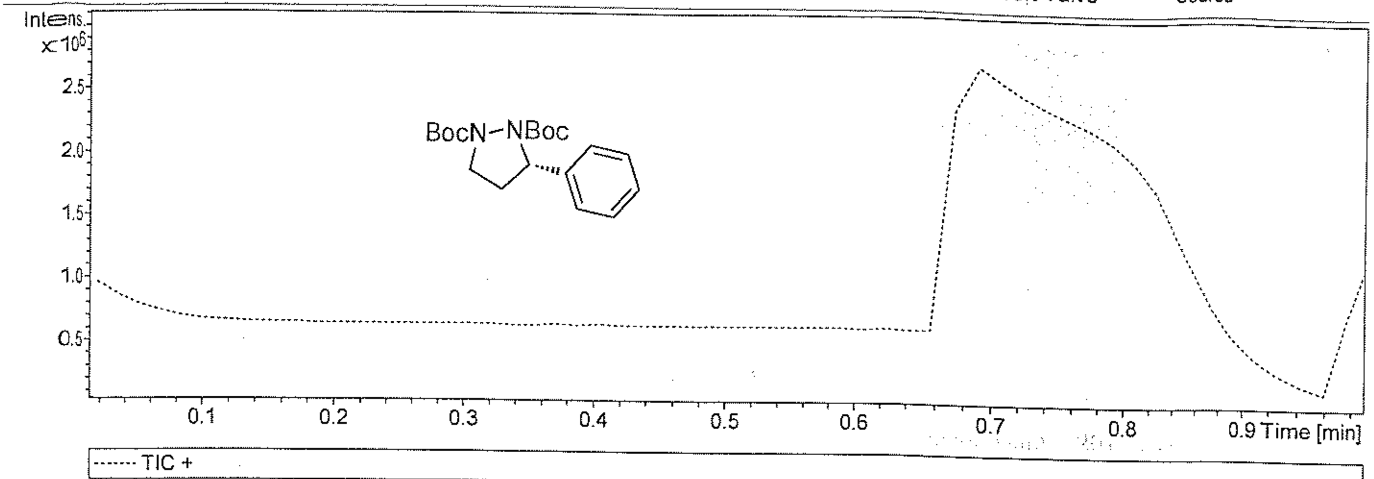

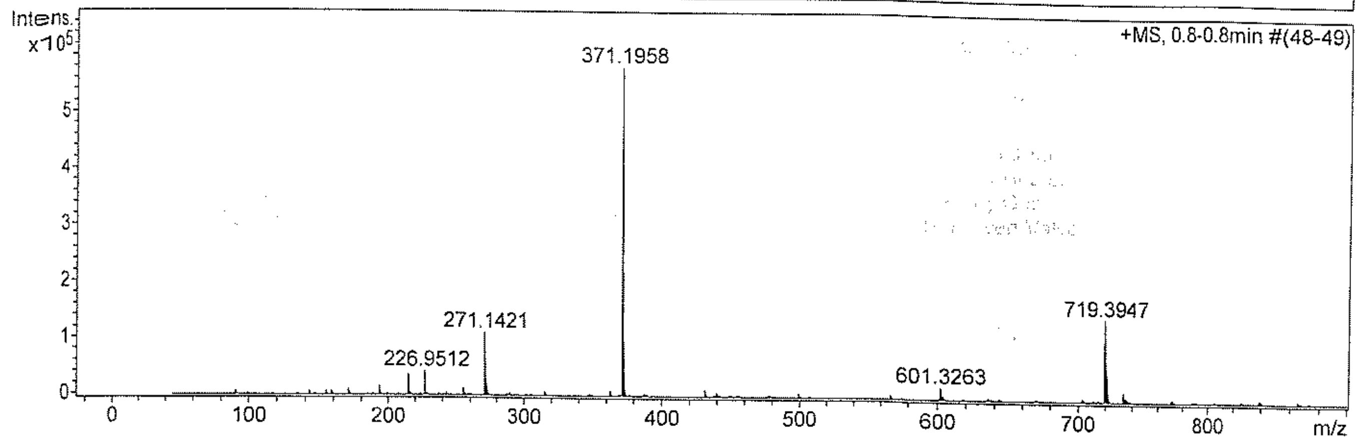

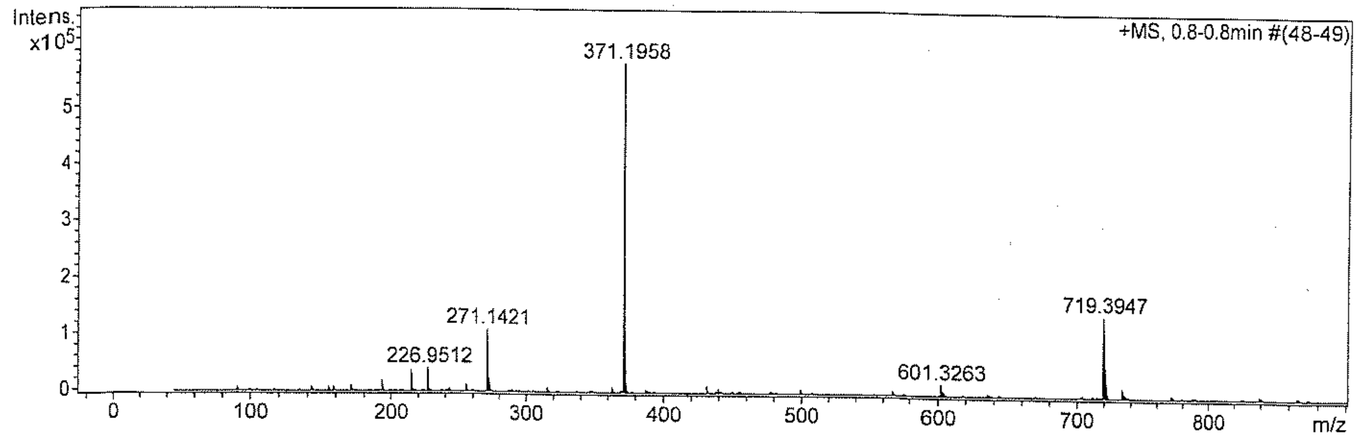

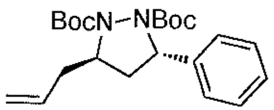
BocN–NBoc

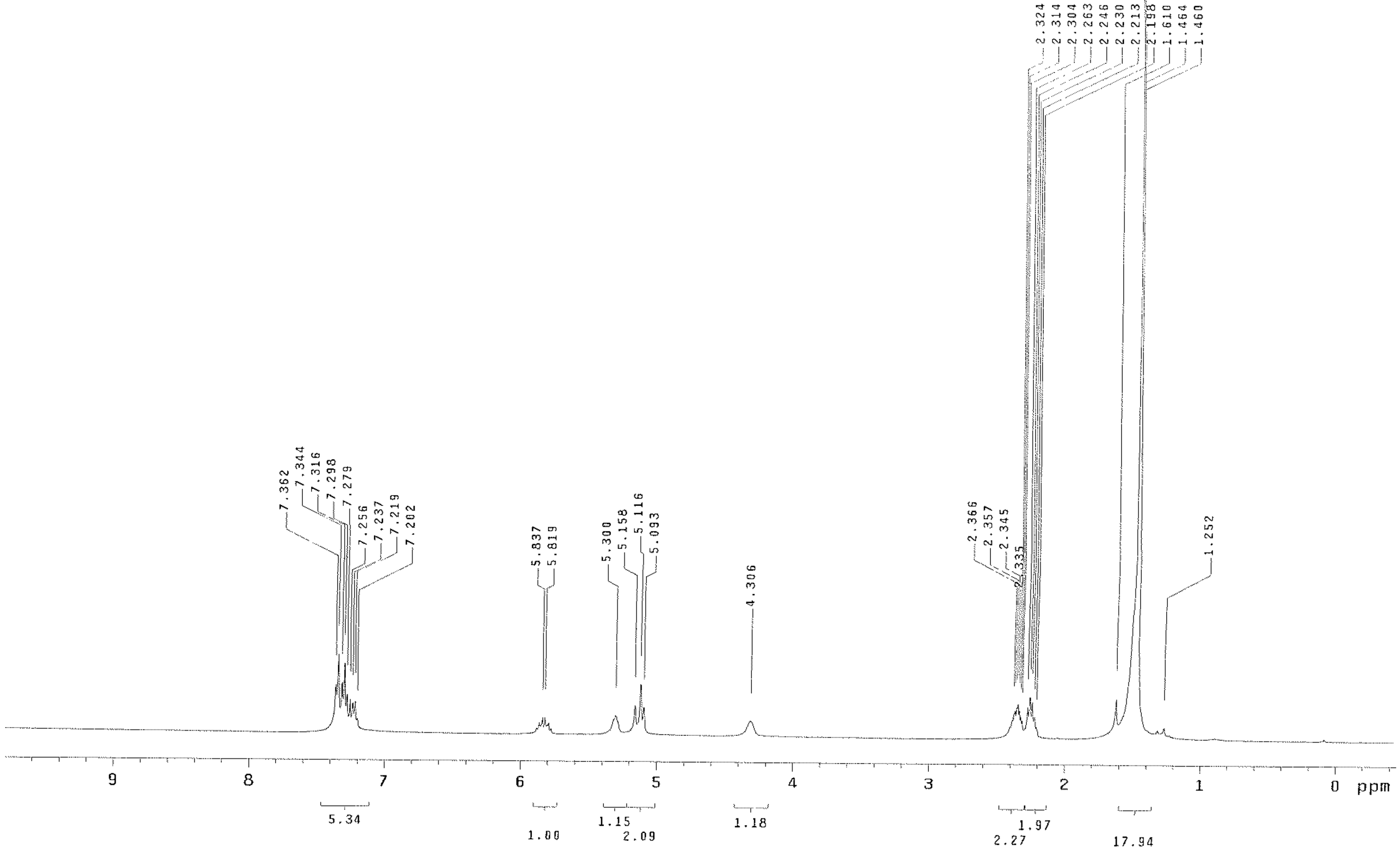
2.324
2.314
2.304
2.263
2.246
2.230
2.213
2.198
1.610
1.464
1.460
7.362
7.344
7.316
7.298
7.279
7.256
7.237
7.219
7.202
5.837
5.819
5.300
5.158
5.116
5.093
4.306
2.366
2.357
2.345
2.335
1.252
9
8
7
6
5
4
3
2
1
0
ppm
5.34
1.00
1.15
2.09
1.18
2.27
1.97
17.94

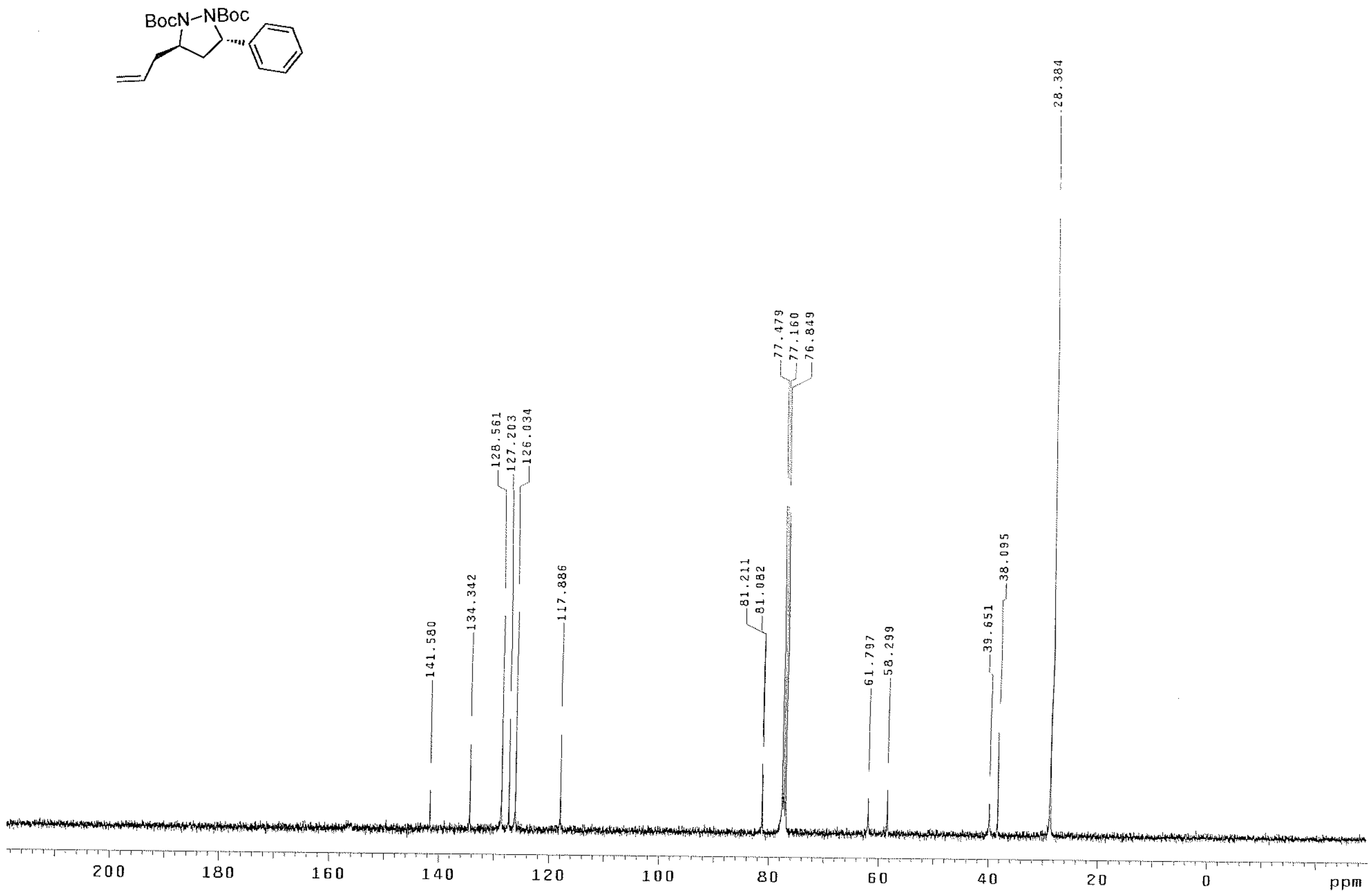
BocN–NBoc
141.580
134.342
128.561
127.203
126.034
117.886
81.211
81.082
77.479
77.160
76.849
61.797
58.299
39.651
38.095
28.384
200
180
160
140
120
100
80
60
40
20
0
ppm

Display Report

Analysis Info

Analysis Name	H:\Data2\Luca\ld745000001.d	Acquisition Date	2011-03-07 12:11:19
Method	tune_low_dirk.m	Operator	pia
Sample Name	ld745	Instrument / Ser#	micrOTOF 125
Comment			

Acquisition Parameter

Source Type	ESI	Ion Polarity	Positive	Set Nebulizer	0.4 Bar
Focus	Not active			Set Dry Heater	170 °C
Scan Begin	50 m/z	Set Capillary	4500 V	Set Dry Gas	4.0 l/min
Scan End	3000 m/z	Set End Plate Offset	-500 V	Set Divert Valve	Source

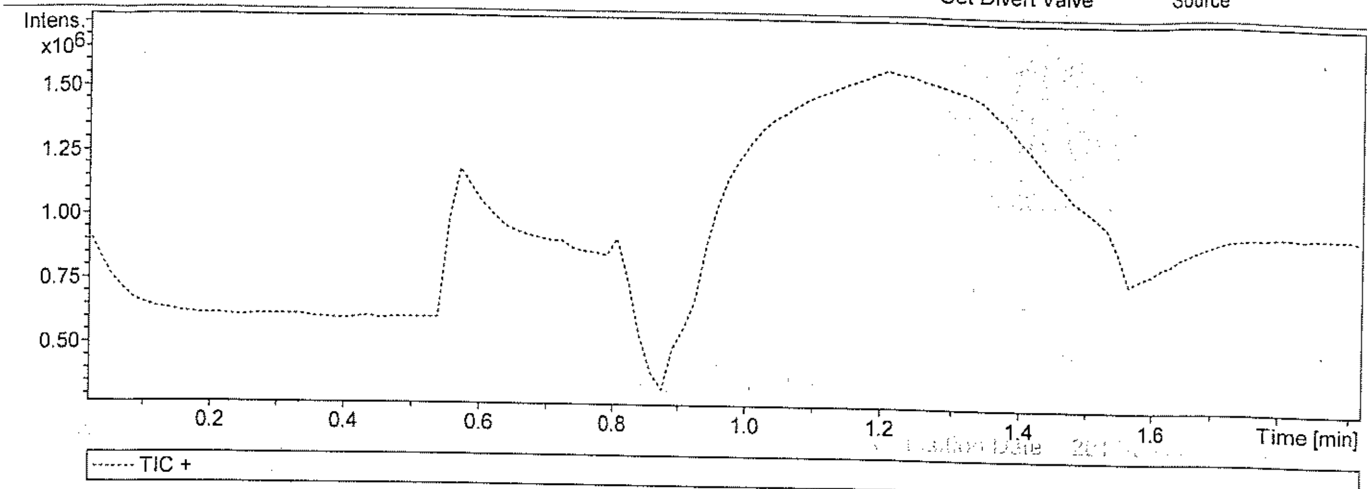

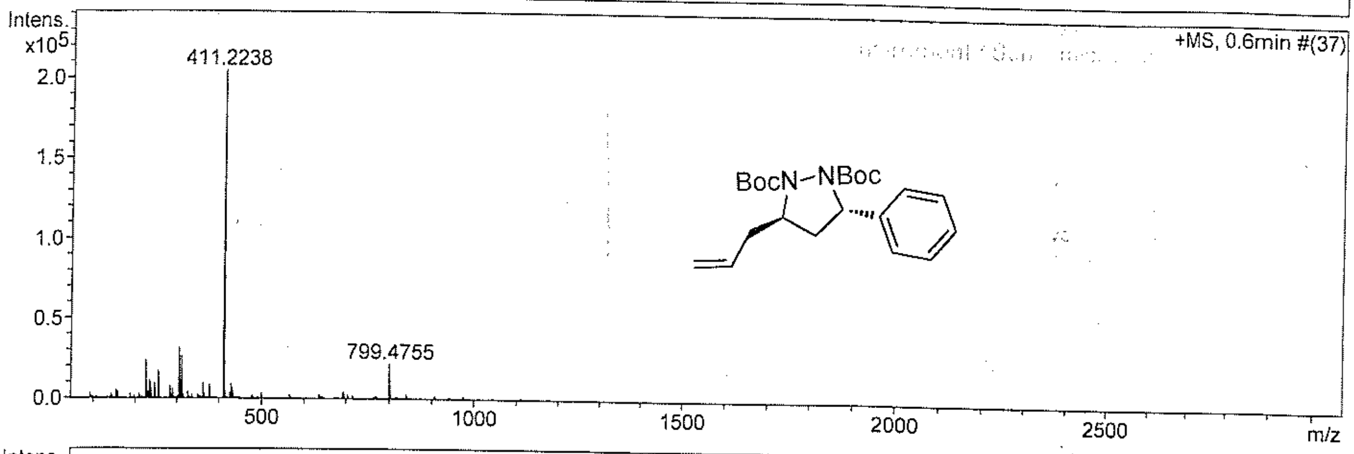

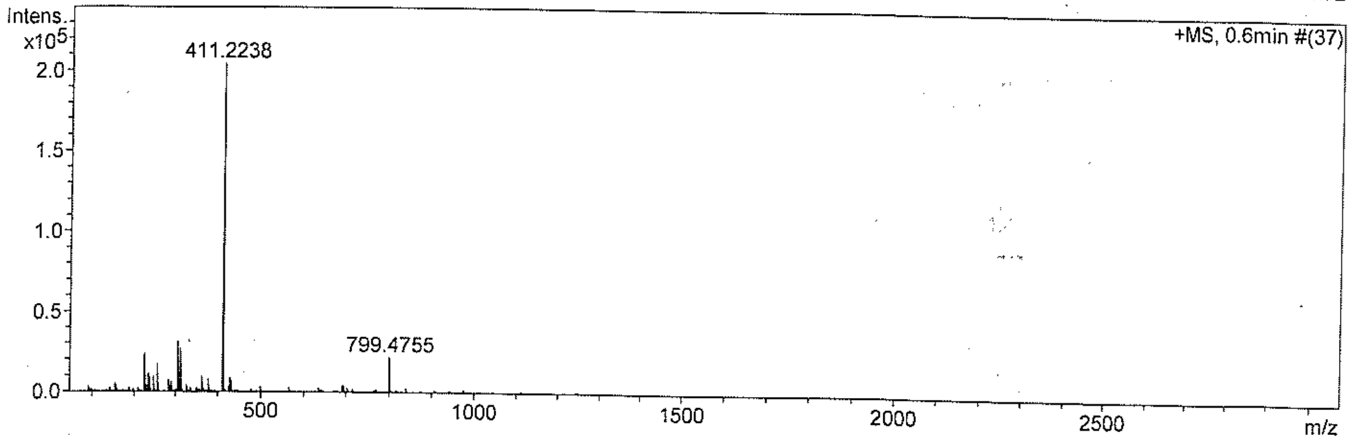

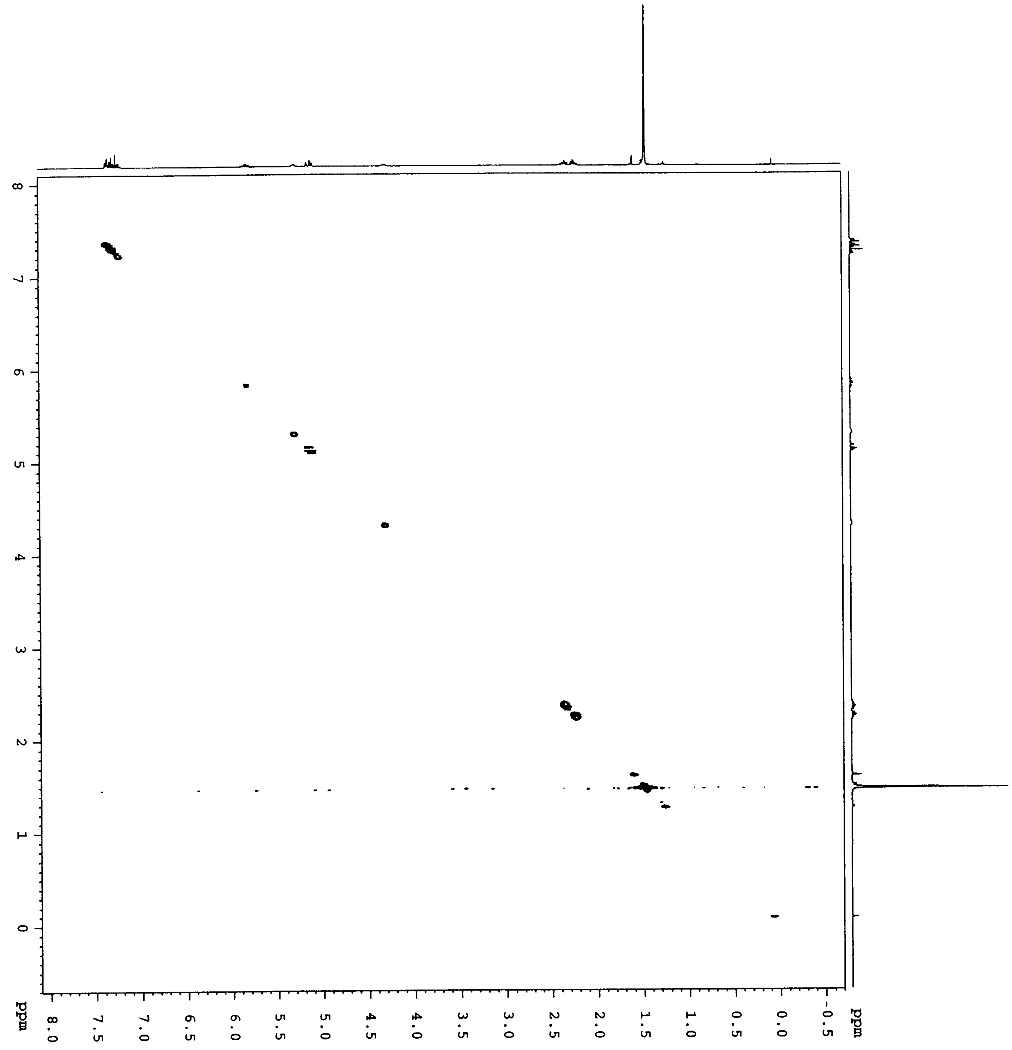
ppm
8.0
7.5
7.0
6.5
6.0
5.5
5.0
4.5
4.0
3.5
3.0
2.5
2.0
1.5
1.0
0.5
0.0
-0.5

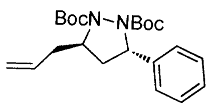
BocN
NBoc

66B-H

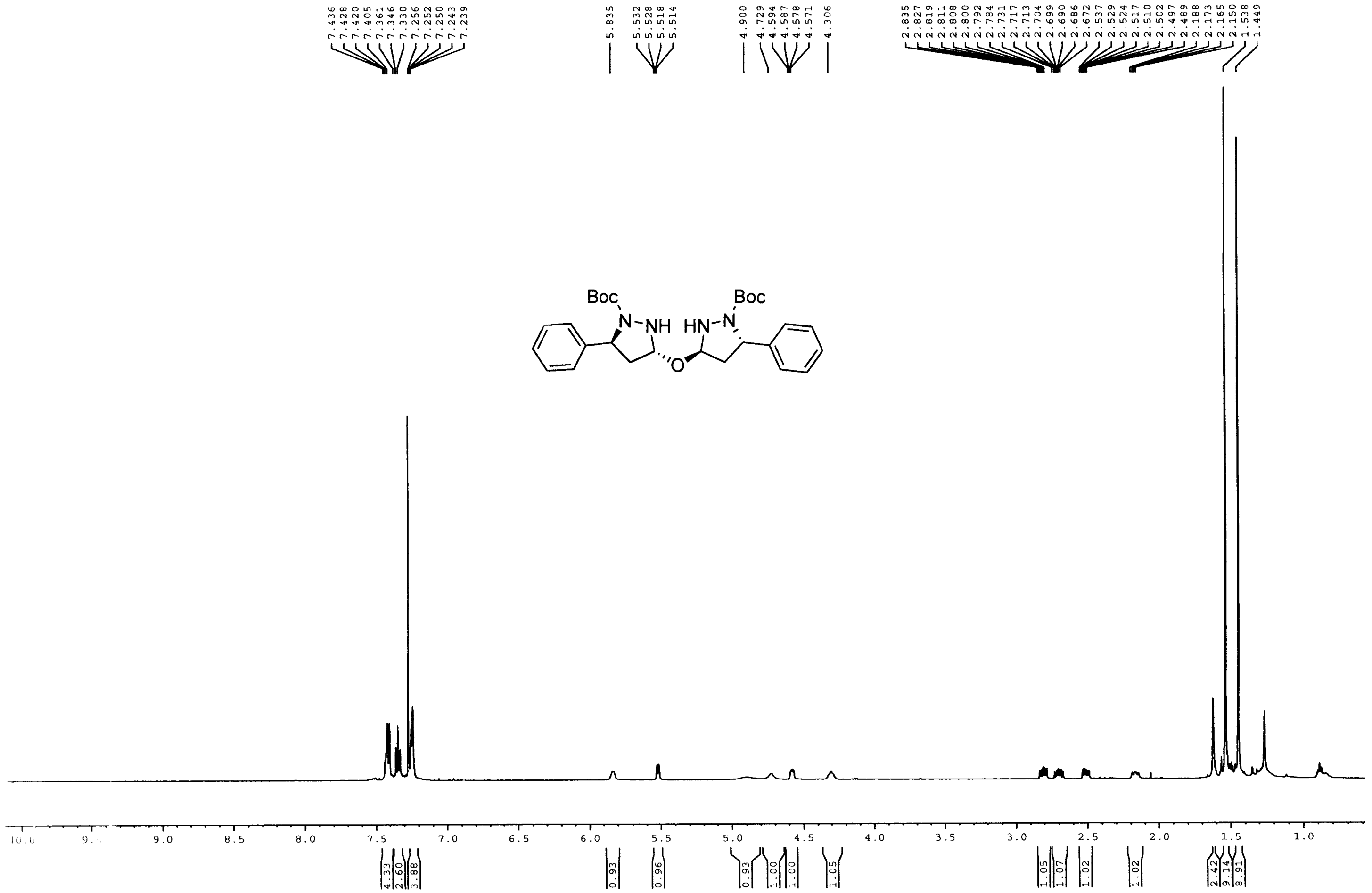
7.436
7.428
7.420
7.405
7.361
7.346
7.330
7.256
7.252
7.250
7.243
7.239
5.835
5.532
5.528
5.518
5.514
4.900
4.729
4.594
4.587
4.578
4.571
4.306
2.835
2.827
2.819
2.811
2.808
2.800
2.792
2.784
2.731
2.717
2.713
2.704
2.699
2.690
2.686
2.672
2.537
2.529
2.524
2.517
2.510
2.502
2.497
2.489
2.188
2.173
2.165
2.150
1.538
1.449
Boc
N
NH
HN
N
Boc
O
9.0
8.5
8.0
7.5
7.0
6.5
6.0
5.5
5.0
4.5
4.0
3.5
3.0
2.5
2.0
1.5
1.0
4.33
2.60
3.88
0.93
0.96
0.93
1.00
1.00
1.05
1.05
1.07
1.02
1.02
2.42
9.14
8.91

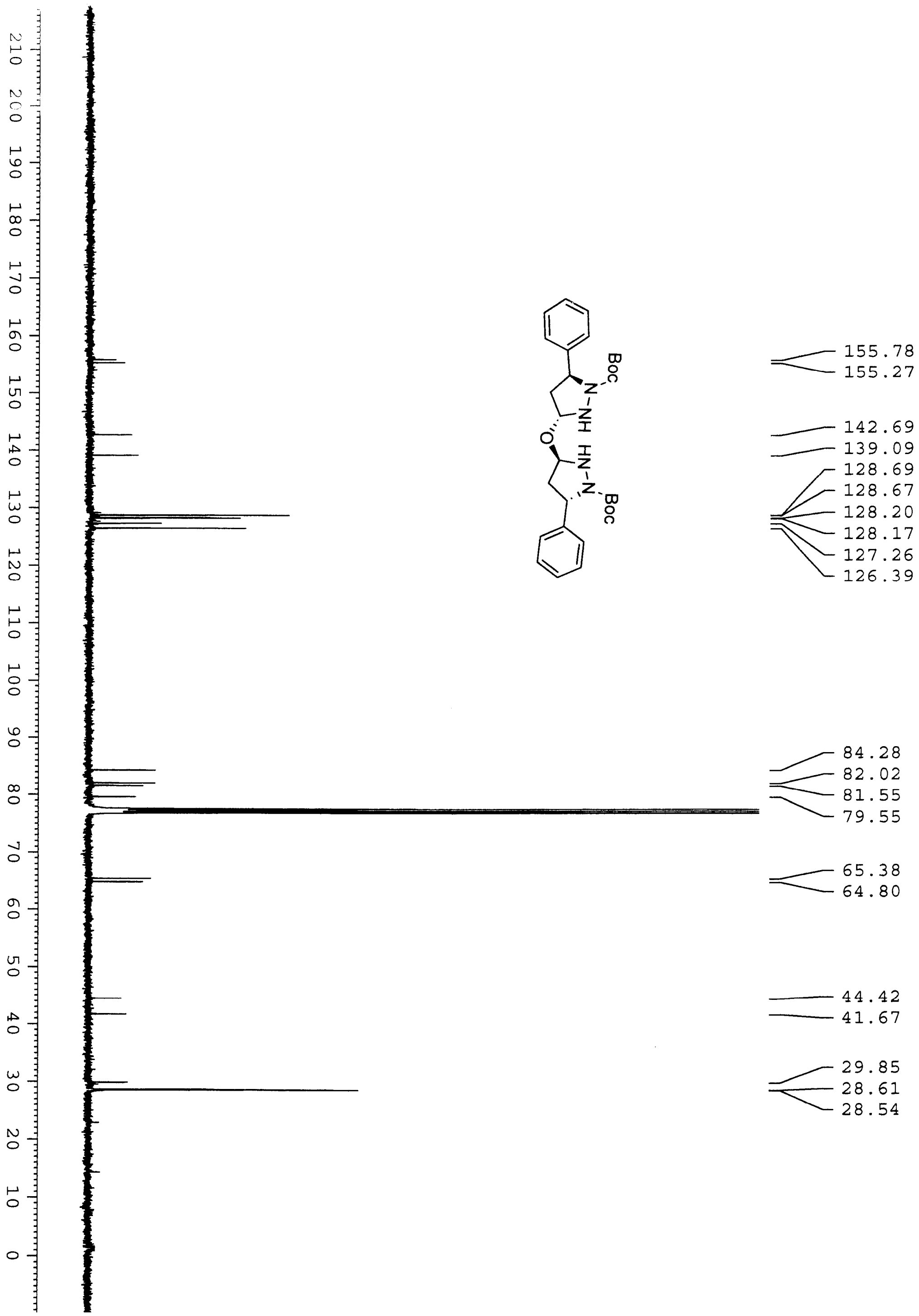

155.78
155.27
142.69
139.09
128.69
128.67
128.20
128.17
127.26
126.39
84.28
82.02
81.55
79.55
65.38
64.80
44.42
41.67
29.85
28.61
28.54
Boc
N-NH
O
HN-N
Boc
210 200 190 180 170 160 150 140 130 120 110 100 90 80 70 60 50 40 30 20 10 0

Display Report

Analysis Info

Analysis Name	H:\Data2\Luca\ld966b000001.d	Acquisition Date	2011-11-30 15:24:08
Method	tune_low_dirk.m	Operator	pia
Sample Name	ld966b	Instrument / Ser#	micrOTOF 125
Comment	2. batch + 0,2eq diisopropyl amine overnight 60oC		

Acquisition Parameter

Source Type	ESI	Ion Polarity	Positive	Set Nebulizer	0.4 Bar
Focus	Not active			Set Dry Heater	170 °C
Scan Begin	50 m/z	Set Capillary	4500 V	Set Dry Gas	4.0 l/min
Scan End	3000 m/z	Set End Plate Offset	-500 V	Set Divert Valve	Source

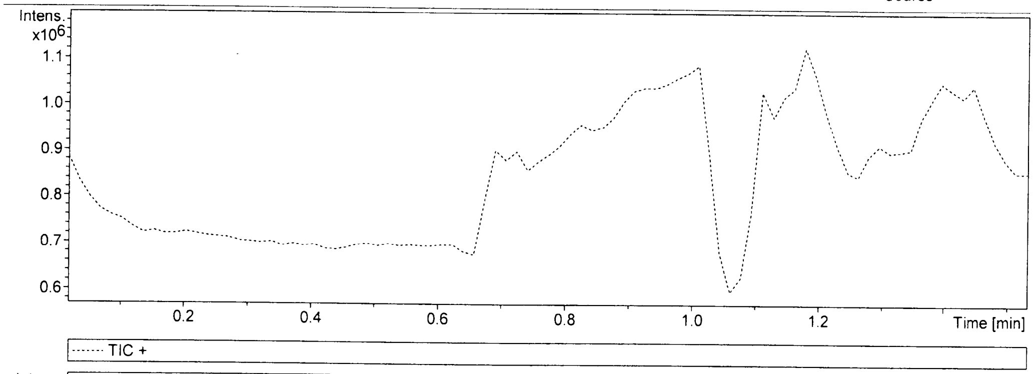

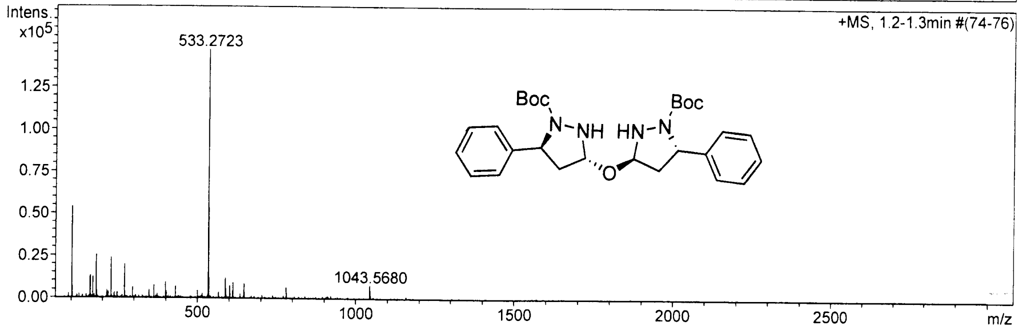

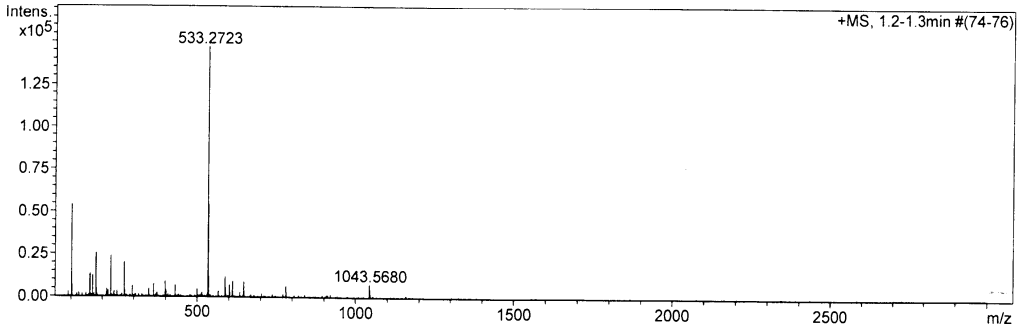

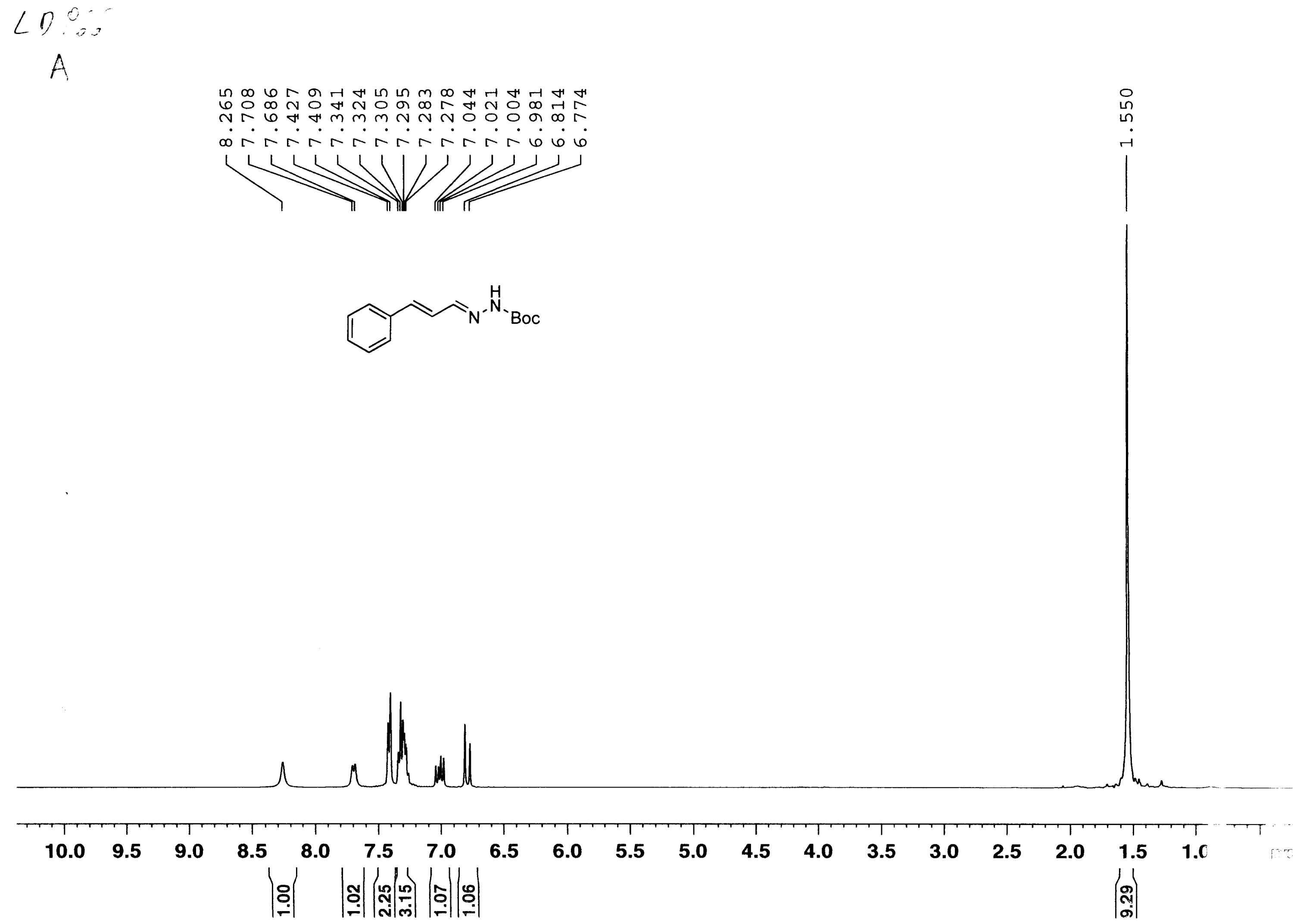

A
8.265
7.708
7.686
7.427
7.409
7.341
7.324
7.305
7.295
7.283
7.278
7.044
7.021
7.004
6.981
6.814
6.774
1.550
H
N
N
Boc
10.0
9.5
9.0
8.5
8.0
7.5
7.0
6.5
6.0
5.5
5.0
4.5
4.0
3.5
3.0
2.5
2.0
1.5
1.00
1.02
2.25
3.15
1.07
1.06
9.29

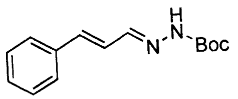
H
N
N
Boc

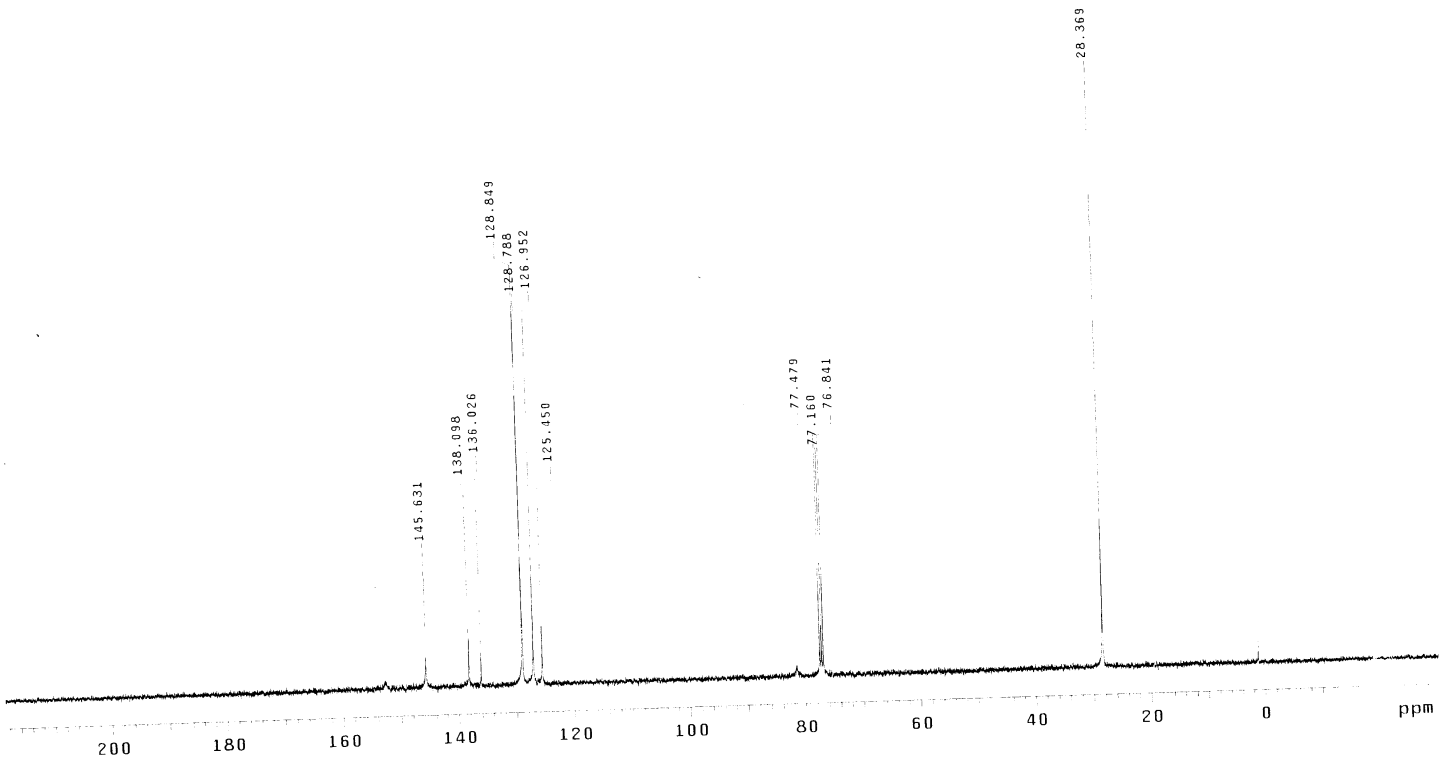
145.631
138.098
136.026
128.849
128.788
126.952
125.450
77.479
77.160
76.841
28.369
200
180
160
140
120
100
80
60
40
20
0
ppm

Display Report

Analysis Info

Analysis Name	H:\Data2\Luca\ld966a000001.d	Acquisition Date	2011-12-01 13:49:46
Method	tune_low_dirk.m	Operator	pia
Sample Name	ld966a	Instrument / Ser#	micrOTOF 125
Comment	sample from the H2O phase (without much precipitate)		

Acquisition Parameter

Source Type	ESI	Ion Polarity	Positive	Set Nebulizer	0.4 Bar
Focus	Not active			Set Dry Heater	170 °C
Scan Begin	50 m/z	Set Capillary	4500 V	Set Dry Gas	4.0 l/min
Scan End	3000 m/z	Set End Plate Offset	-500 V	Set Divert Valve	Source

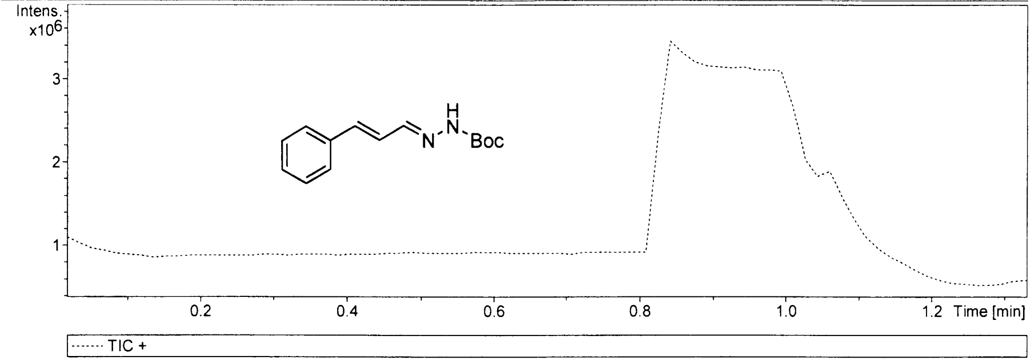

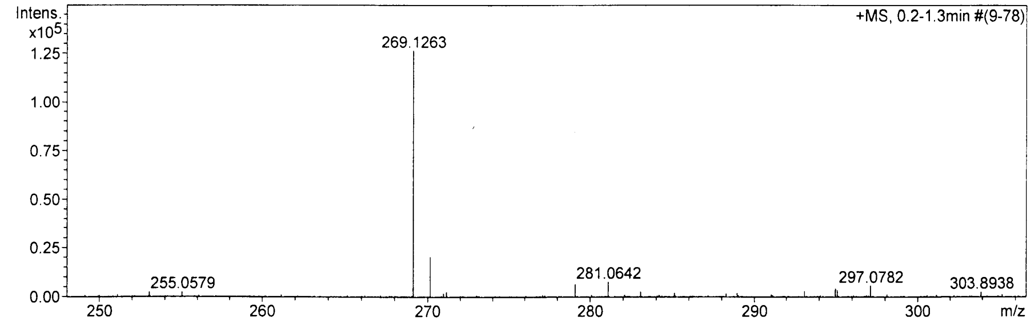

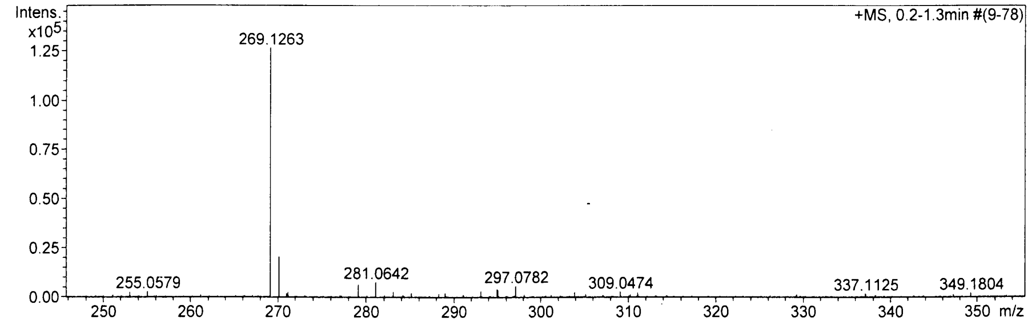

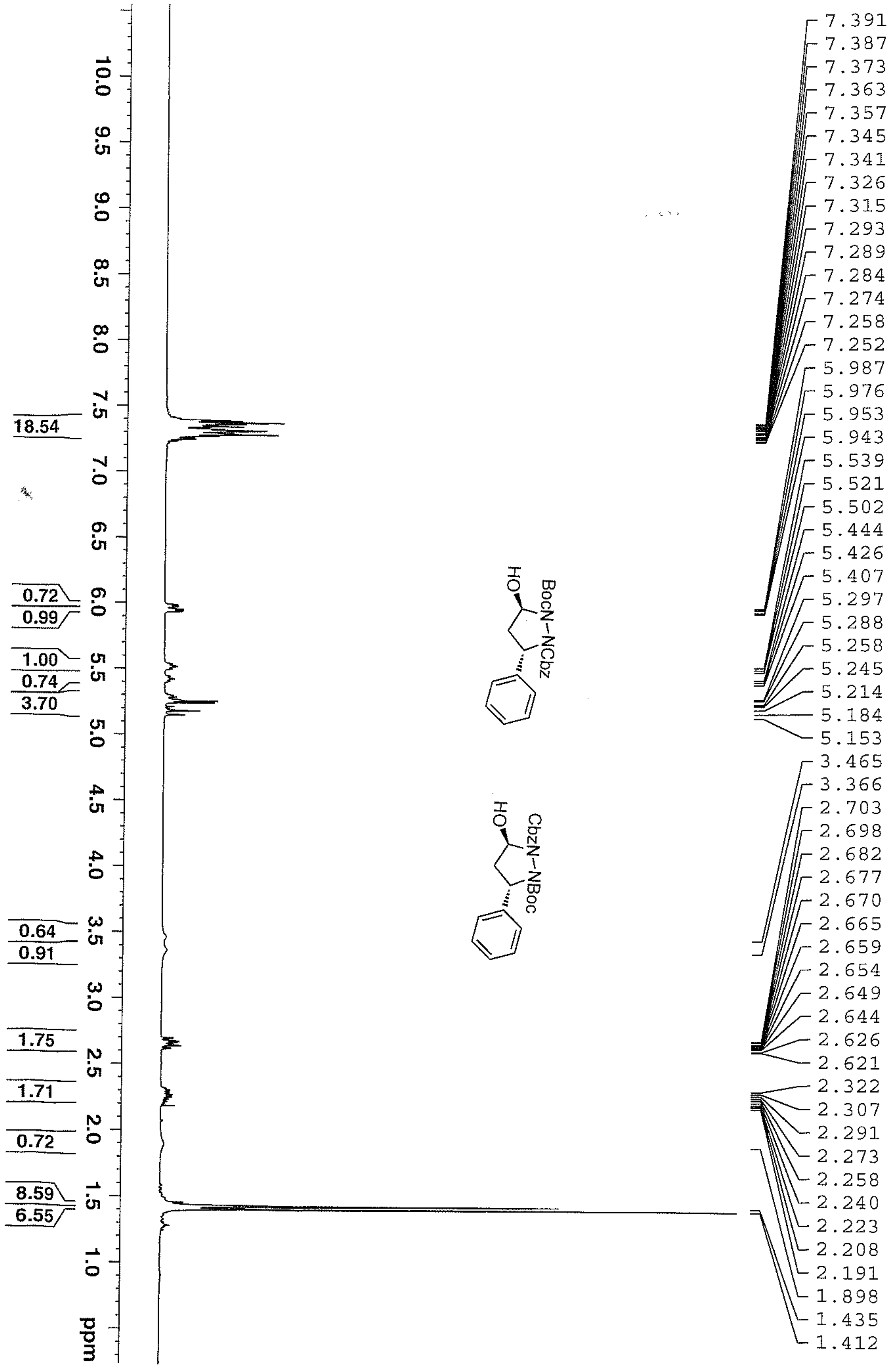
7.391
7.387
7.373
7.363
7.357
7.345
7.341
7.326
7.315
7.293
7.289
7.284
7.274
7.258
7.252
5.987
5.976
5.953
5.943
5.539
5.521
5.502
5.444
5.426
5.407
5.297
5.288
5.258
5.245
5.214
5.184
5.153
3.465
3.366
2.703
2.698
2.682
2.677
2.670
2.665
2.659
2.654
2.649
2.644
2.626
2.621
2.322
2.307
2.291
2.273
2.258
2.240
2.223
2.208
2.191
1.898
1.435
1.412
18.54
0.72
0.99
1.00
0.74
3.70
0.64
0.91
1.75
1.71
0.72
8.59
6.55
10.0
9.5
9.0
8.5
8.0
7.5
7.0
6.5
6.0
5.5
5.0
4.5
4.0
3.5
3.0
2.5
2.0
1.5
1.0
ppm
BocN–NCbz
HO
CbzN–NBoc
HO

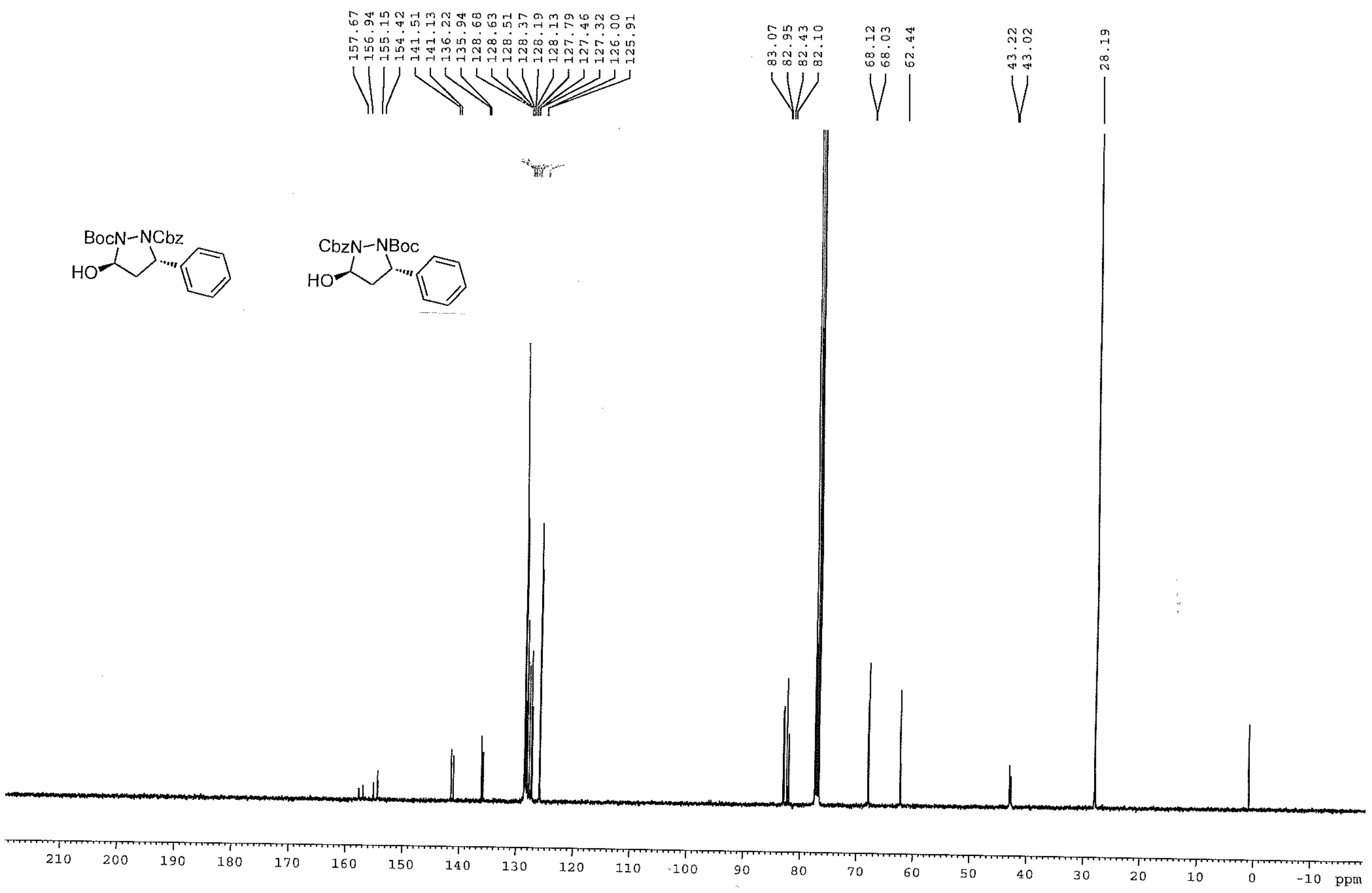

BocN–NCbz
HO
CbzN–NBoc
HO
157.67
156.94
155.15
154.42
141.51
141.13
136.22
135.94
128.68
128.63
128.51
128.37
128.19
128.13
127.79
127.46
127.32
126.00
125.91
83.07
82.95
82.43
82.10
68.12
68.03
62.44
43.22
43.02
28.19
210 200 190 180 170 160 150 140 130 120 110 100 90 80 70 60 50 40 30 20 10 0 -10 ppm

Display Report

Analysis Info

Analysis Name	H:\Data2\Luca\ld935000001.d	Acquisition Date	2011-11-17 12:38:14
Method	tune_low_dirk.m	Operator	pia
Sample Name	ld935	Instrument / Ser#	micrOTOF 125
Comment			

Acquisition Parameter

Source Type	ESI	Ion Polarity	Positive	Set Nebulizer	0.4 Bar
Focus	Not active			Set Dry Heater	170 °C
Scan Begin	50 m/z	Set Capillary	4500 V	Set Dry Gas	4.0 l/min
Scan End	3000 m/z	Set End Plate Offset	-500 V	Set Divert Valve	Source

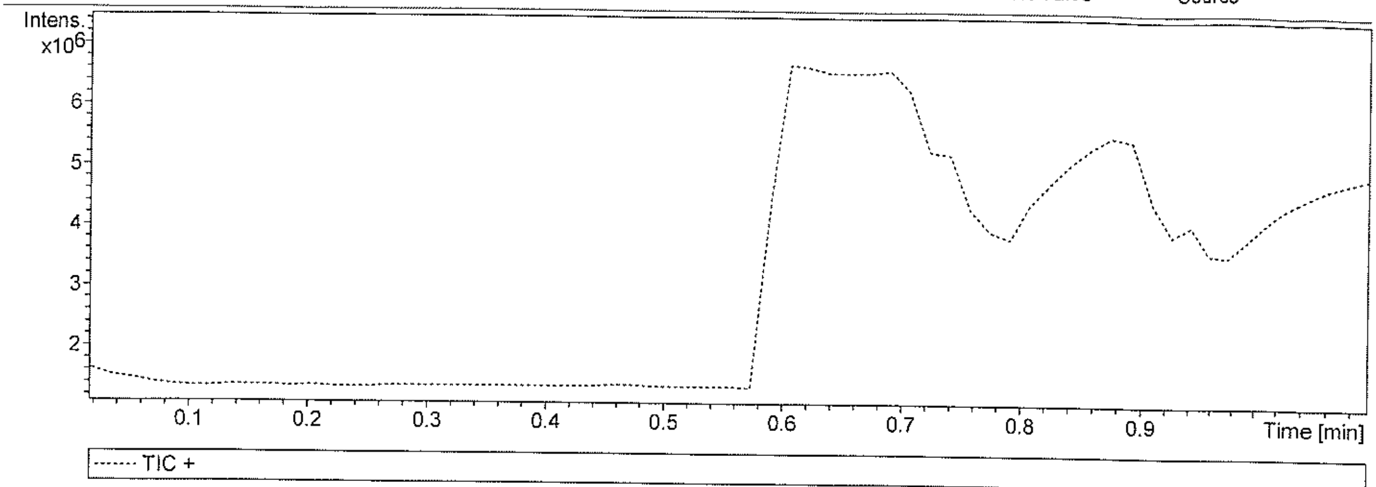

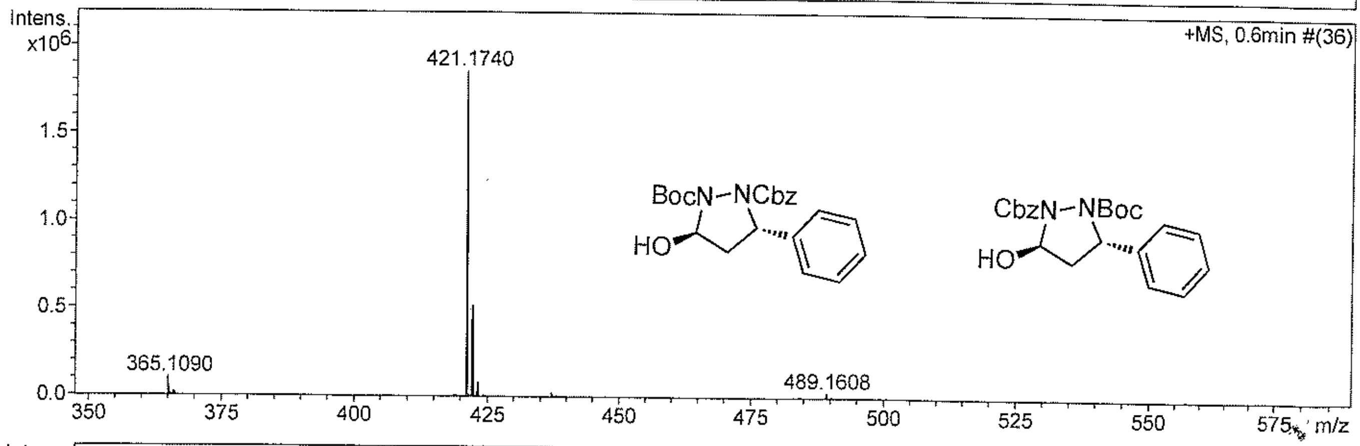

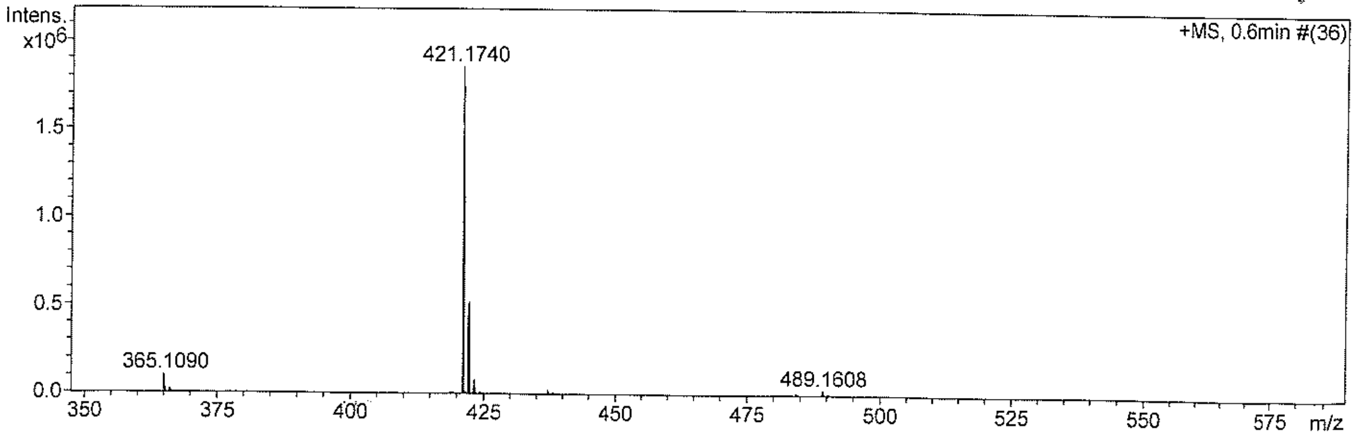

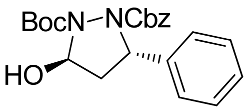

BocN
NCbz
HO

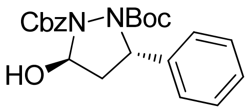

CbzN
NBoc
HO

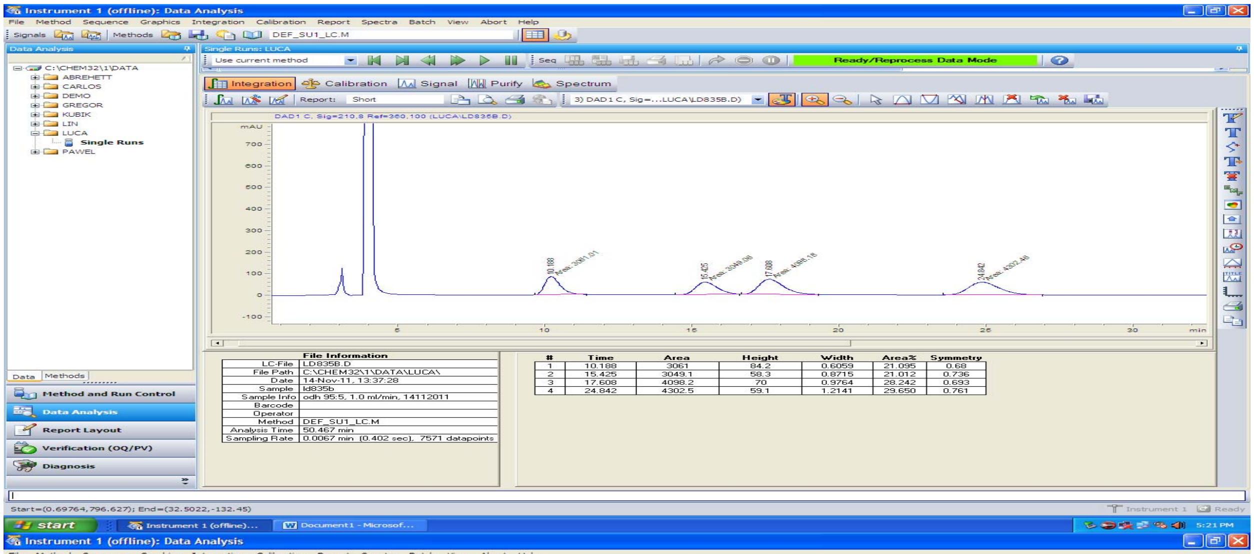

Instrument 1 (offline): Data Analysis
DAD1 C, Sig=210,8 Ref=360,100 (LUCA\LD835B.D)
File Information
LC-File LD835B.D
File Path C:\CHEM32\1\DATA\LUCA\
Date 14-Nov-11, 13:37:28
Sample ld835b
Sample Info odh 95:5, 1.0 ml/min, 14112011
Method DEF_SU1_LC.M
Analysis Time 50.467 min
Sampling Rate 0.0067 min (0.402 sec), 7571 datapoints
Time Area Height Width Area% Symmetry
1 10.188 3061 84.2 0.6059 21.095 0.68
2 15.425 3049.1 58.3 0.8715 21.012 0.736
3 17.608 4098.2 70 0.9764 28.242 0.693
4 24.842 4302.5 59.1 1.2141 29.650 0.761

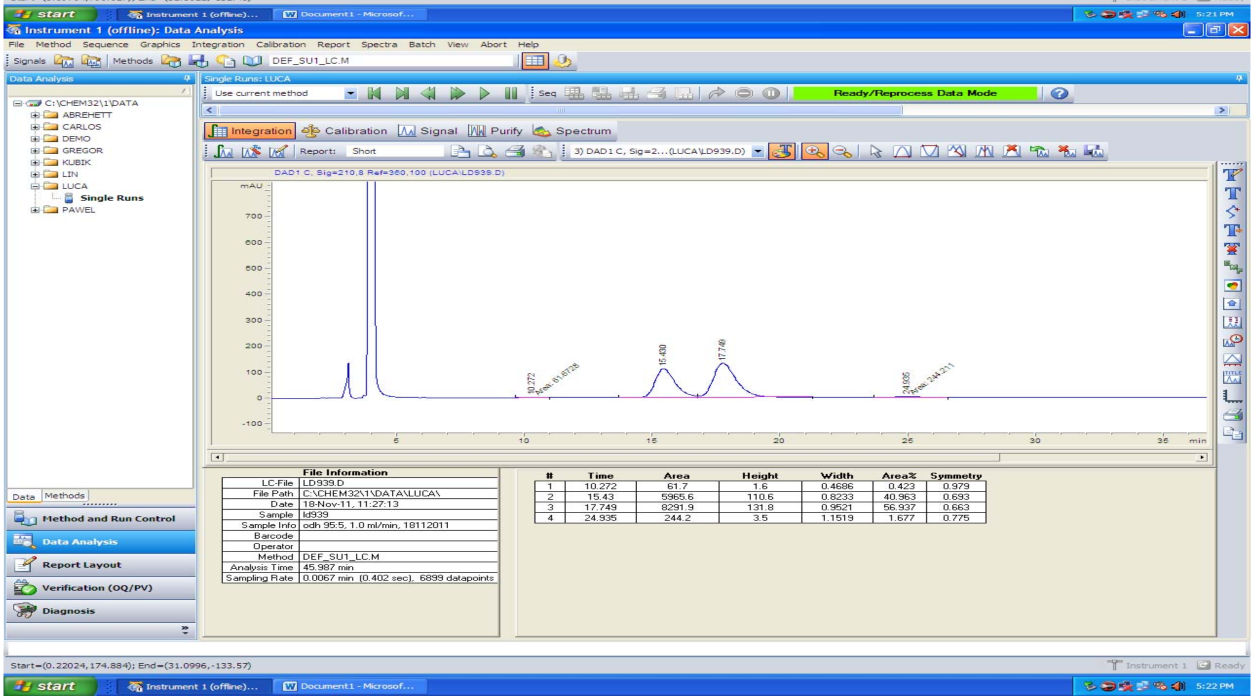

Instrument 1 (offline): Data Analysis
DAD1 C, Sig=210,8 Ref=360,100 (LUCA\LD939.D)
File Information
LC-File LD939.D
File Path C:\CHEM32\1\DATA\LUCA\
Date 18-Nov-11, 11:27:13
Sample ld939
Sample Info odh 95:5, 1.0 ml/min, 18112011
Method DEF_SU1_LC.M
Analysis Time 45.987 min
Sampling Rate 0.0067 min (0.402 sec), 6899 datapoints
Time Area Height Width Area% Symmetry
1 10.272 61.7 1.6 0.4686 0.423 0.979
2 15.43 5965.6 110.6 0.8233 40.963 0.693
3 17.749 8291.9 131.8 0.9521 56.937 0.663
4 24.935 244.2 3.5 1.1519 1.677 0.775

LD 997
ALD

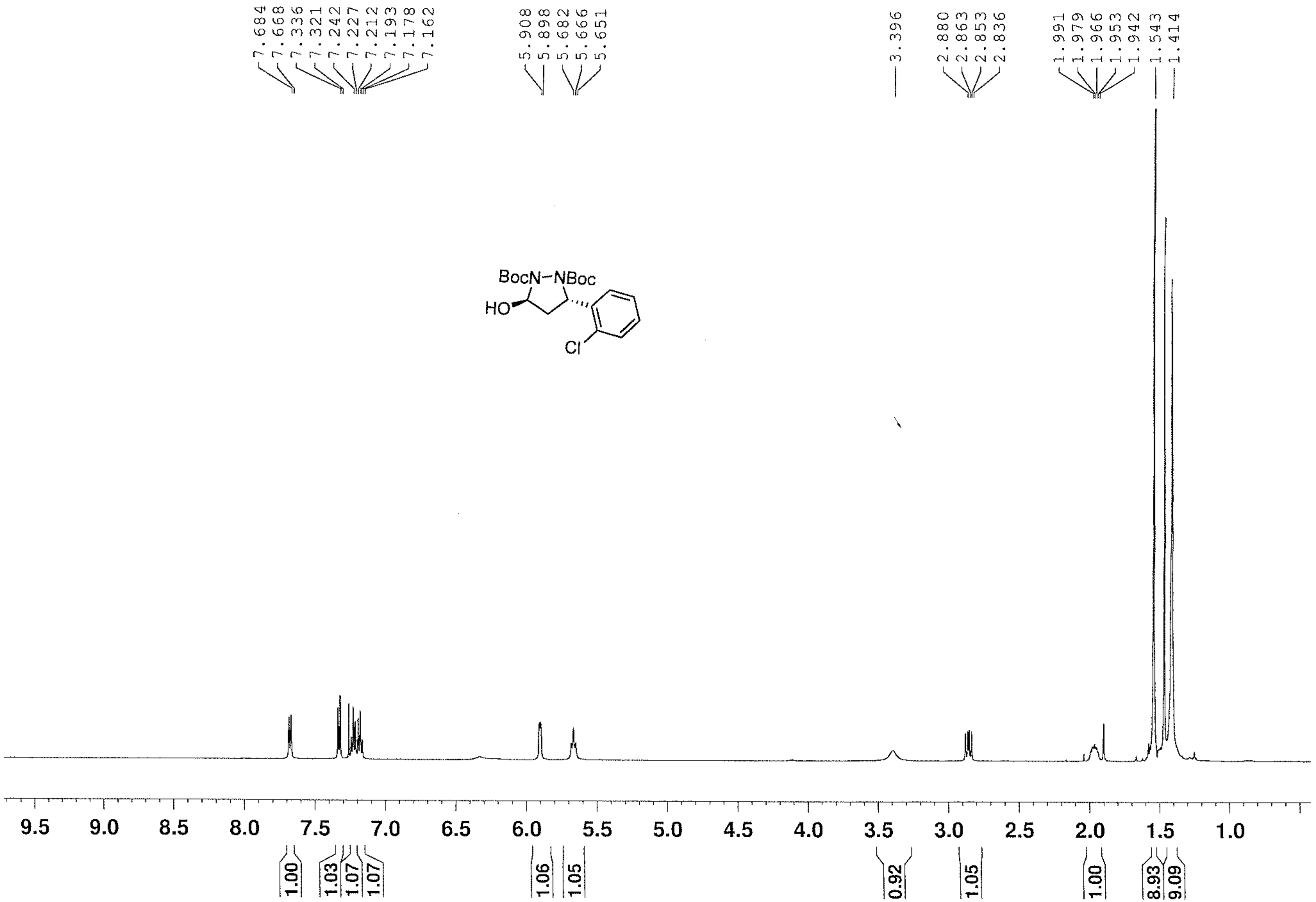

7.684
7.668
7.336
7.321
7.242
7.227
7.212
7.193
7.178
7.162
5.908
5.898
5.682
5.666
5.651
3.396
2.880
2.863
2.853
2.836
1.991
1.979
1.966
1.953
1.942
1.543
1.414
BocN–NBoc
HO
Cl
9.5
9.0
8.5
8.0
7.5
7.0
6.5
6.0
5.5
5.0
4.5
4.0
3.5
3.0
2.5
2.0
1.5
1.0
1.00
1.03
1.07
1.07
1.06
1.05
0.92
1.05
1.00
8.93
9.09

997C2

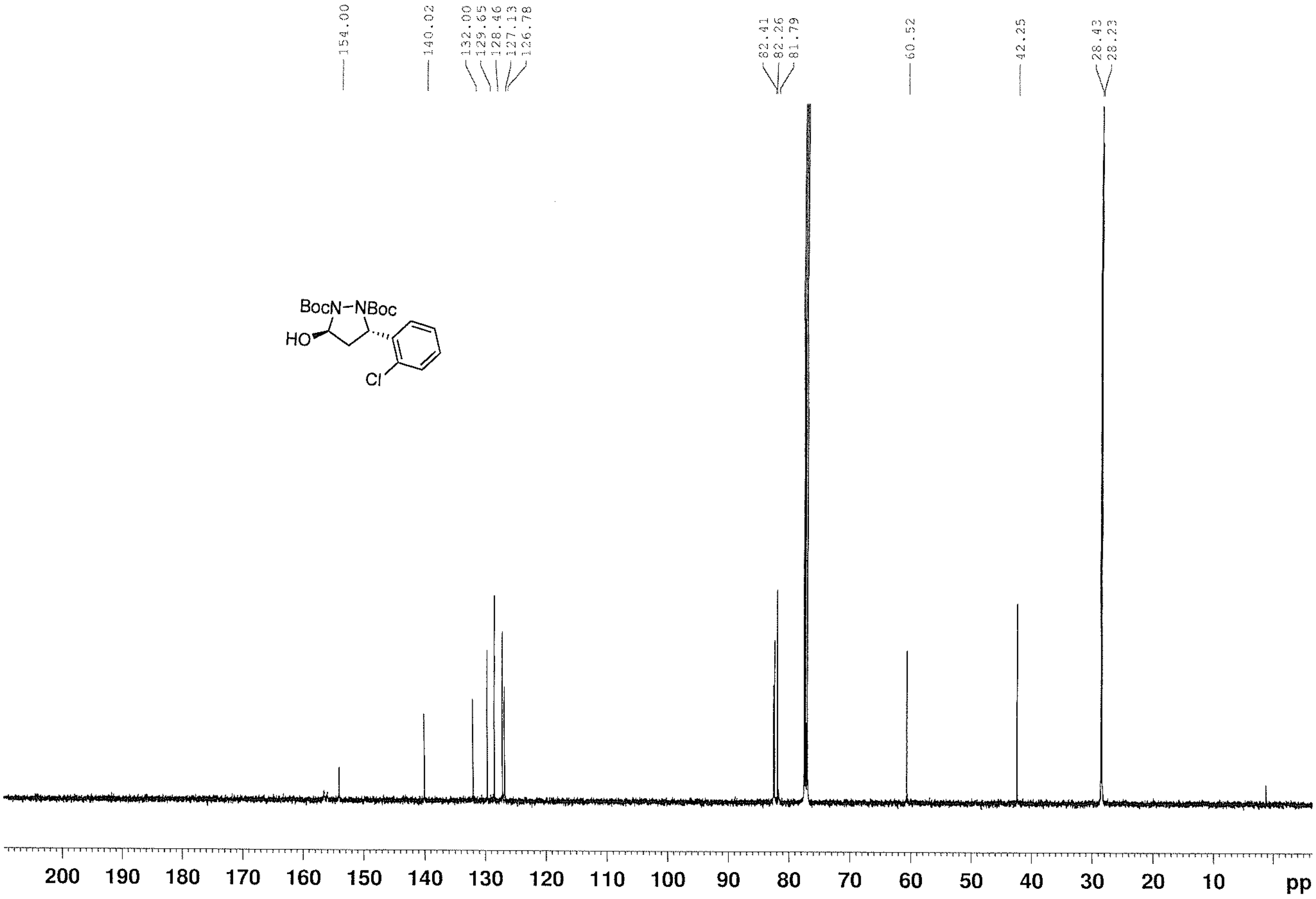

154.00
140.02
132.00
129.65
128.46
127.13
126.78
82.41
82.26
81.79
60.52
42.25
28.43
28.23
BocN–NBoc
HO
Cl
200
190
180
170
160
150
140
130
120
110
100
90
80
70
60
50
40
30
20
10
pp

Display Report

Analysis Info

Analysis Name	H:\Data2\Luca\ld995000001.d	Acquisition Date	2012-01-19 15:36:21
Method	tune_low_dirk.m	Operator	pia
Sample Name	ld995	Instrument / Ser#	micrOTOF 125
Comment			

Acquisition Parameter

Source Type	ESI	Ion Polarity	Positive	Set Nebulizer	0.4 Bar
Focus	Not active			Set Dry Heater	170 °C
Scan Begin	50 m/z	Set Capillary	4500 V	Set Dry Gas	4.0 l/min
Scan End	3000 m/z	Set End Plate Offset	-500 V	Set Divert Valve	Source

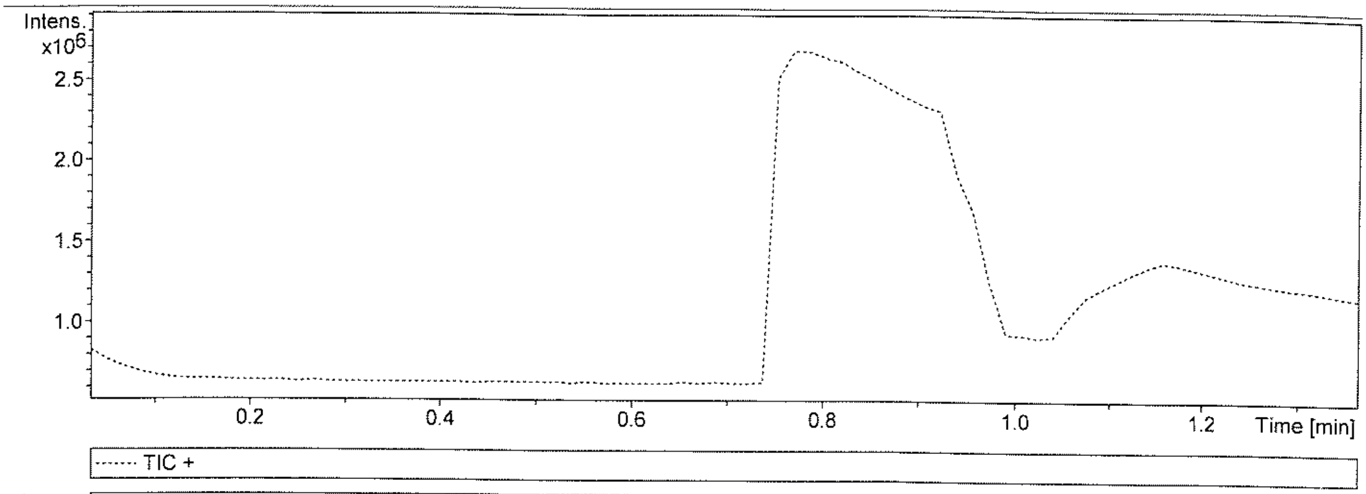

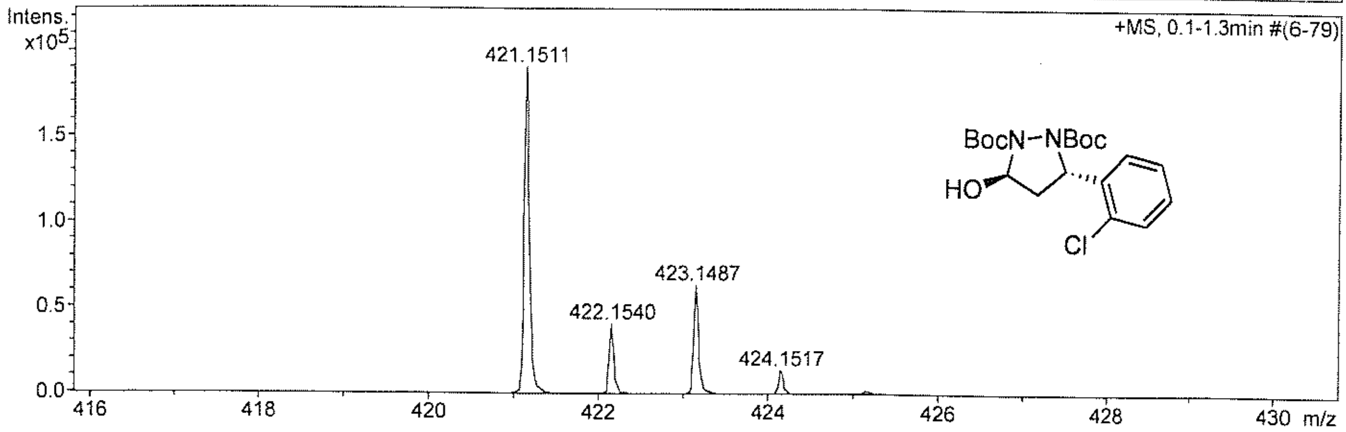

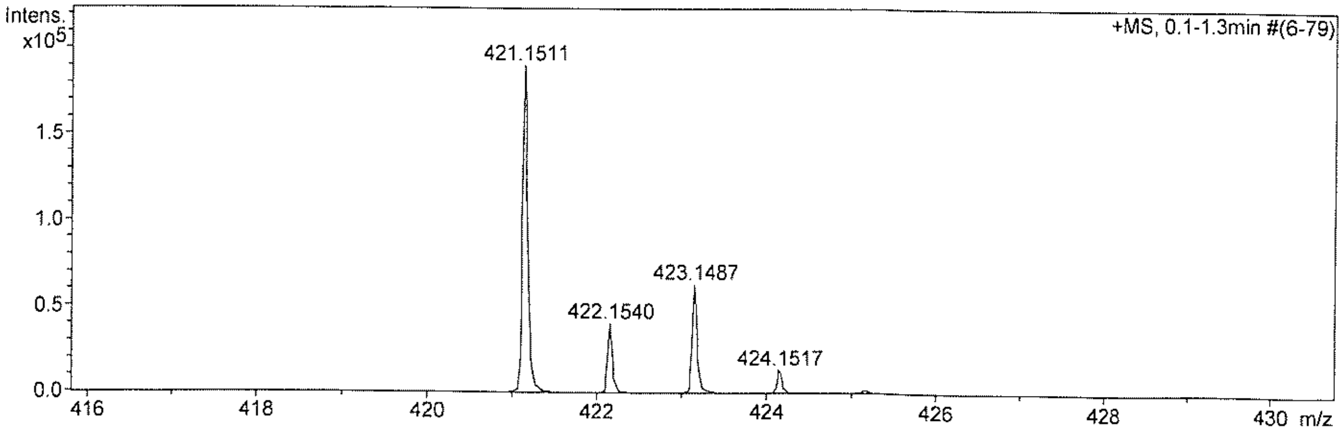

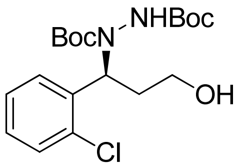
NHBoc
BocN
OH
Cl

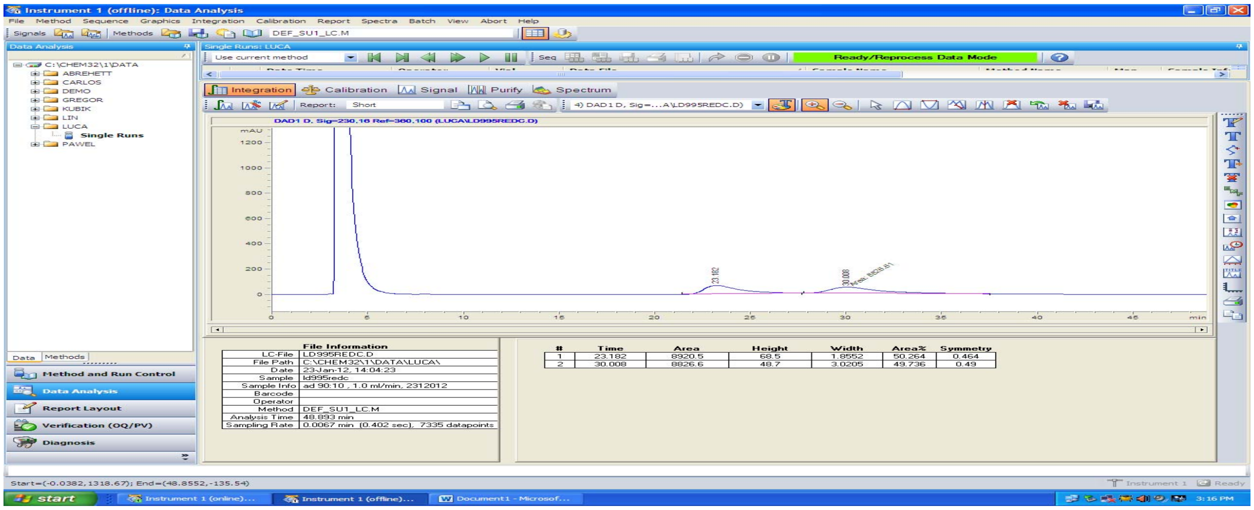
Instrument 1 (offline): Data Analysis
DAD1 D, Sig=230,16 Ref=360,100 (LUCA\LD995REDC.D)
23.182
30.008
File Information
LC-File LD995REDC.D
File Path C:\CHEM32\1\DATA\LUCA\
Date 23-Jan-12, 14:04:23
Sample ld995redc
Sample Info ad 90:10 , 1.0 ml/min, 2312012
Method DEF_SU1_LC.M
Analysis Time 48.893 min
Sampling Rate 0.0067 min (0.402 sec), 7335 datapoints
Time Area Height Width Area% Symmetry
1 23.182 8920.5 68.5 1.8552 50.264 0.464
2 30.008 8826.6 48.7 3.0205 49.736 0.49

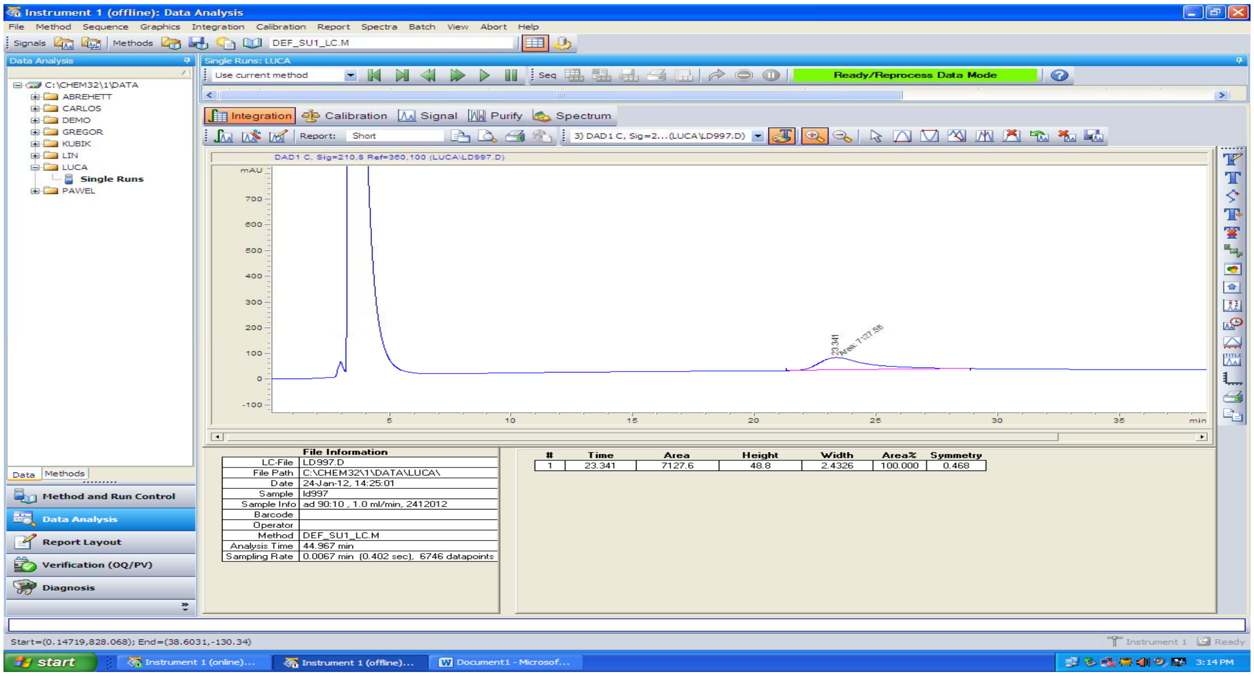
Instrument 1 (offline): Data Analysis
DAD1 C, Sig=210,8 Ref=360,100 (LUCA\LD997.D)
23.341
File Information
LC-File LD997.D
File Path C:\CHEM32\1\DATA\LUCA\
Date 24-Jan-12, 14:25:01
Sample ld997
Sample Info ad 90:10 , 1.0 ml/min, 2412012
Method DEF_SU1_LC.M
Analysis Time 44.967 min
Sampling Rate 0.0067 min (0.402 sec), 6746 datapoints
Time Area Height Width Area% Symmetry
1 23.341 7127.6 48.8 2.4326 100.000 0.468

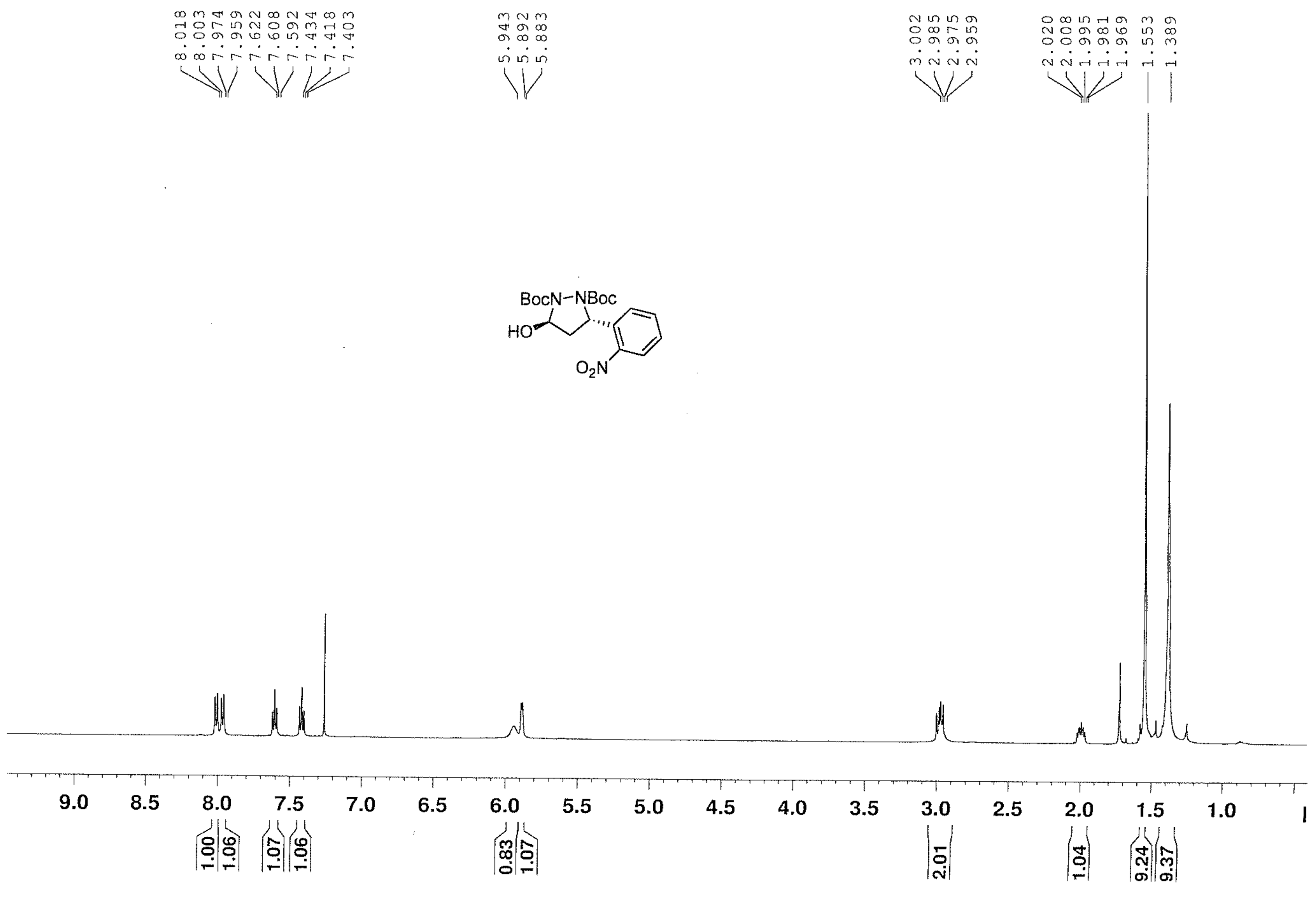
8.018
8.003
7.974
7.959
7.622
7.608
7.592
7.434
7.418
7.403
5.943
5.892
5.883
3.002
2.985
2.975
2.959
2.020
2.008
1.995
1.981
1.969
1.553
1.389
BocN–NBoc
HO
O_2N
9.0
8.5
8.0
7.5
7.0
6.5
6.0
5.5
5.0
4.5
4.0
3.5
3.0
2.5
2.0
1.5
1.0
1.00
1.06
1.07
1.06
0.83
1.07
2.01
1.04
9.24
9.37

996C

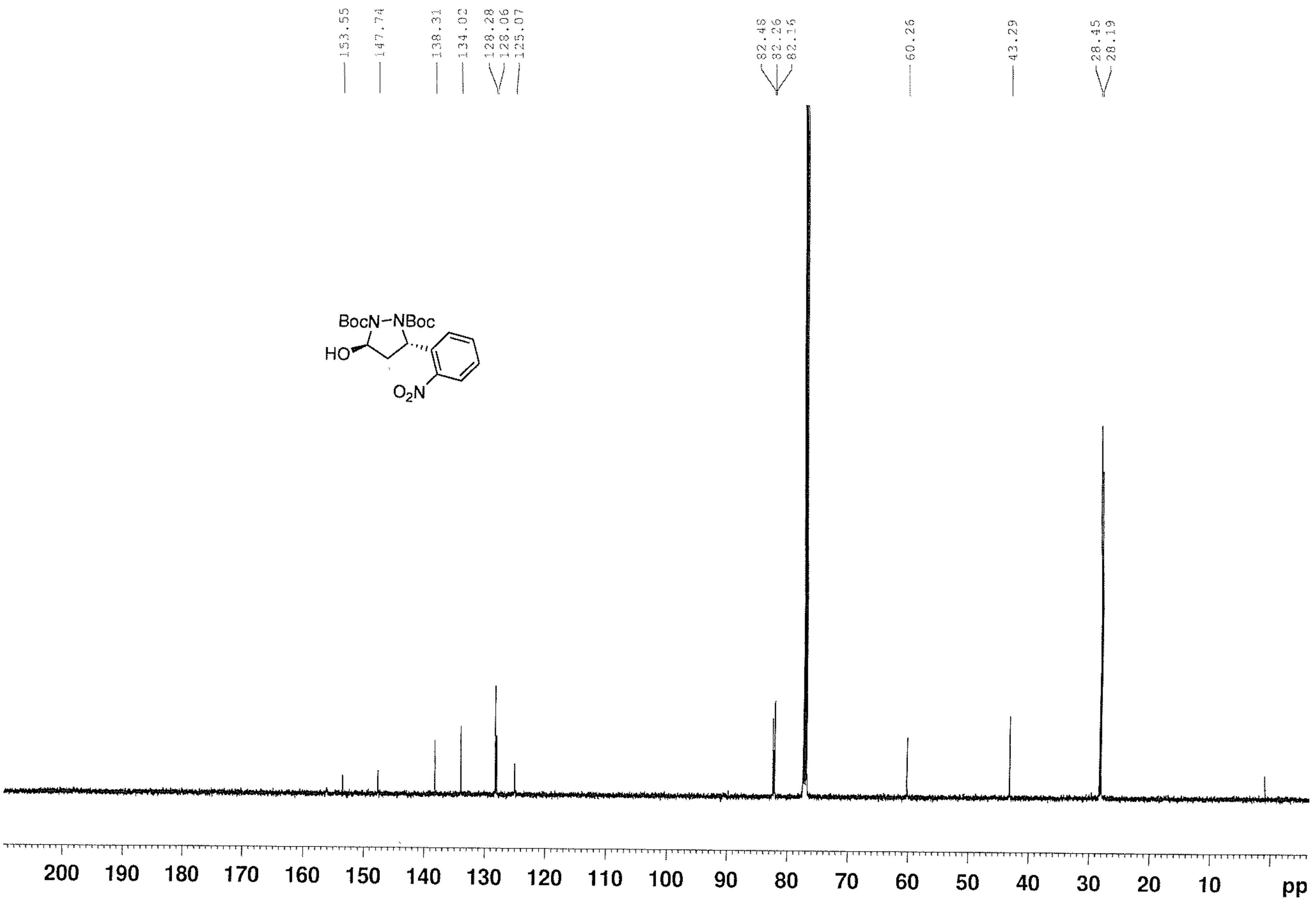
153.55
147.74
138.31
134.02
128.28
128.06
125.07
82.45
82.26
82.16
60.26
43.29
28.45
28.19
BocN–NBoc
HO
O_2N
200
190
180
170
160
150
140
130
120
110
100
90
80
70
60
50
40
30
20
10
pp

Display Report

Analysis Info

Analysis Name	H:\Data2\Luca\ld996000001.d	Acquisition Date	2012-01-19 15:43:15
Method	tune_low_dirk.m	Operator	pia
Sample Name	ld996	Instrument / Ser#	micrOTOF 125
Comment			

Acquisition Parameter

Source Type	ESI	Ion Polarity	Positive	Set Nebulizer	0.4 Bar
Focus	Not active			Set Dry Heater	170 °C
Scan Begin	50 m/z	Set Capillary	4500 V	Set Dry Gas	4.0 l/min
Scan End	3000 m/z	Set End Plate Offset	-500 V	Set Divert Valve	Source

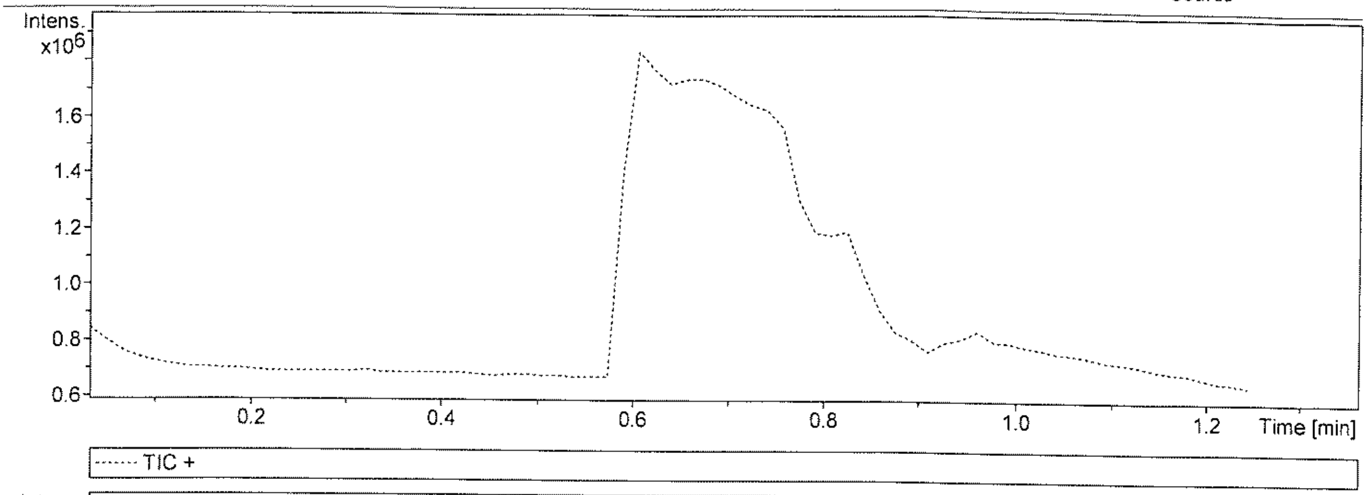

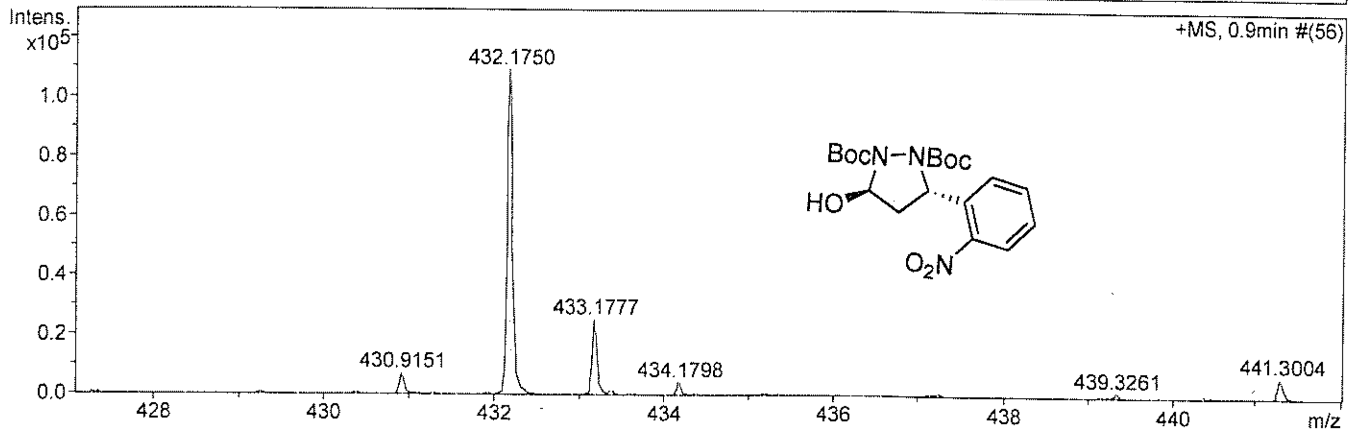

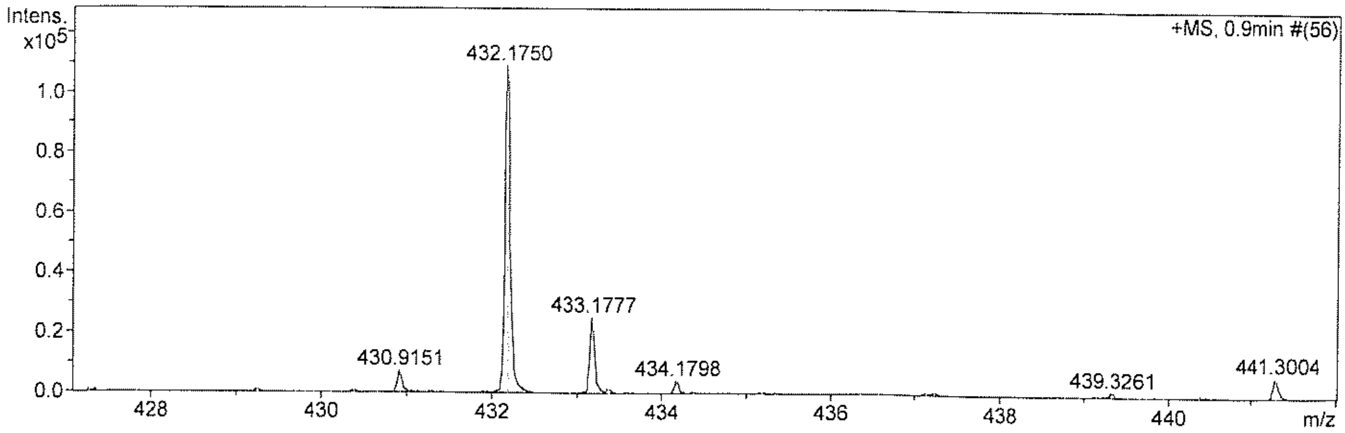

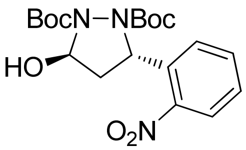
BocN−NBoc
HO
O2N

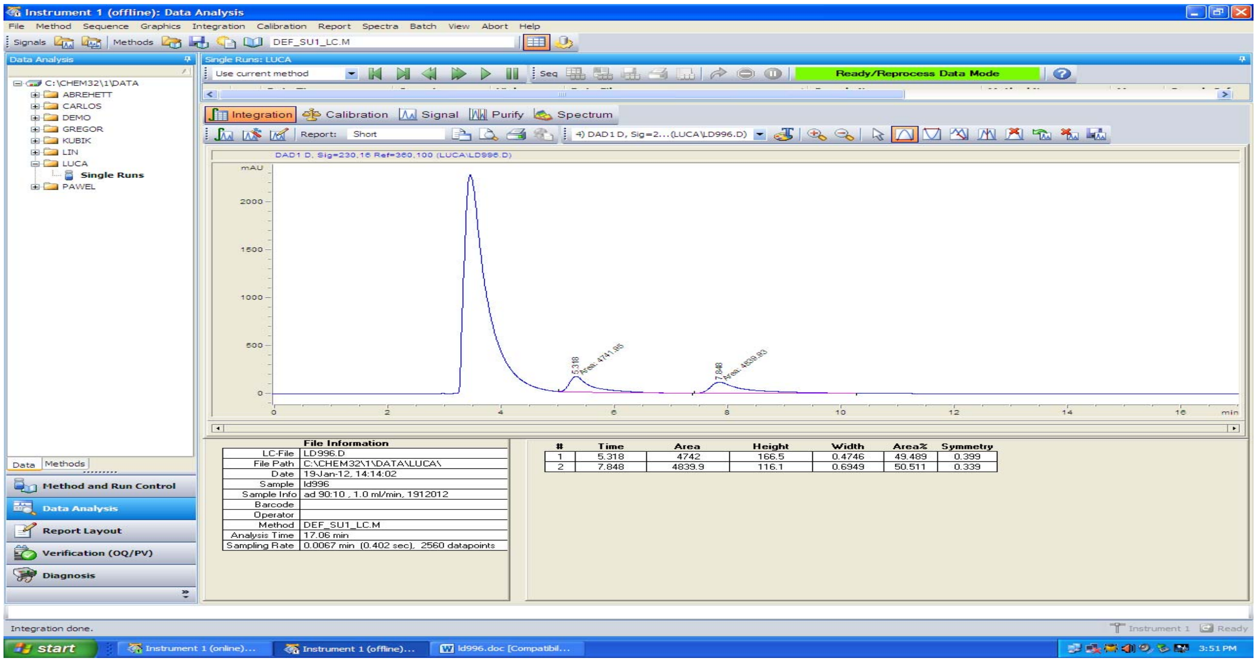
Instrument 1 (offline): Data Analysis
DAD1 D, Sig=230,16 Ref=360,100 (LUCA\LD996.D)
Ready/Reprocess Data Mode
File Information
LC-File LD996.D
File Path C:\CHEM32\1\DATA\LUCA\
Date 19Jan-12, 14:14:02
Sample ld996
Sample Info ad 90:10 , 1.0 ml/min, 1912012
Method DEF_SU1_LC.M
Analysis Time 17.06 min
Sampling Rate 0.0067 min (0.402 sec), 2560 datapoints
Time Area Height Width Area% Symmetry
1 5.318 4742 166.5 0.4746 49.489 0.399
2 7.848 4839.9 116.1 0.6949 50.511 0.339
Integration done.

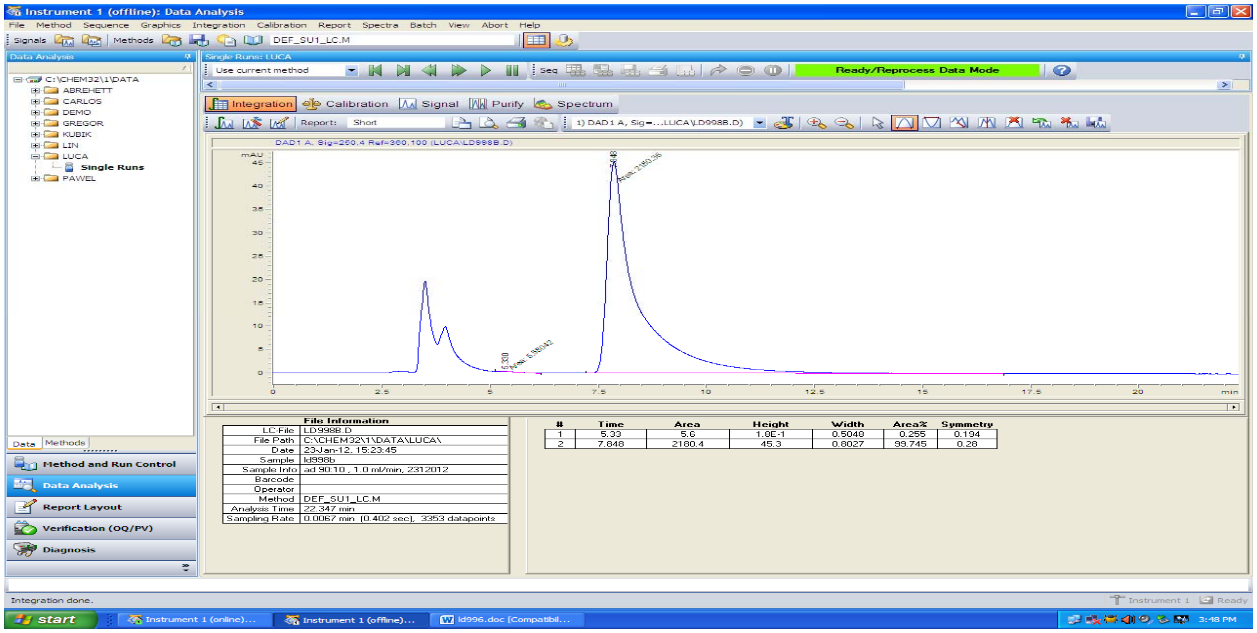
Instrument 1 (offline): Data Analysis
DAD1 A, Sig=260,4 Ref=360,100 (LUCA\LD998B.D)
Ready/Reprocess Data Mode
File Information
LC-File LD998B.D
File Path C:\CHEM32\1\DATA\LUCA\
Date 23-Jan-12, 15:23:45
Sample ld998b
Sample Info ad 90:10 , 1.0 ml/min, 2312012
Method DEF_SU1_LC.M
Analysis Time 22.347 min
Sampling Rate 0.0067 min (0.402 sec), 3353 datapoints
Time Area Height Width Area% Symmetry
1 5.33 5.6 1.8E-1 0.5048 0.255 0.194
2 7.848 2180.4 45.3 0.8027 99.745 0.28
Integration done.
